# Patterns of Element Incorporation in Calcium Carbonate Biominerals Recapitulate Phylogeny for a Diverse Range of Marine Calcifiers

**DOI:** 10.3389/feart.2021.641760

**Published:** 2021-05-04

**Authors:** Robert N. Ulrich, Maxence Guillermic, Julia Campbell, Abbas Hakim, Rachel Han, Shayleen Singh, Justin D. Stewart, Cristian Román-Palacios, Hannah M. Carroll, Ilian De Corte, Rosaleen E. Gilmore, Whitney Doss, Aradhna Tripati, Justin B. Ries, Robert A. Eagle

**Affiliations:** 1 Department of Earth, Planetary, and Space Sciences, University of California, Los Angeles, Los Angeles, CA, United States,; 2 Institute of the Environment and Sustainability, University of California, Los Angeles, Los Angeles, CA, United States,; 3 Center for Diverse Leadership in Science, University of California, Los Angeles, Los Angeles, CA, United States,; 4 Department of Atmospheric and Oceanic Sciences, University of California, Los Angeles, Los Angeles, CA, United States,; 5 Department of Microbiology, Immunology, and Molecular Genetics, University of California, Los Angeles, Los Angeles, CA, United States,; 6 Department of Ecology and Evolutionary Biology, University of California, Los Angeles, Los Angeles, CA, United States,; 7 Department of Integrative Biology and Physiology, University of California, Los Angeles, Los Angeles, CA, United States,; 8 Department of Ecological Science, Vrije Universiteit Amsterdam, Amsterdam, Netherlands,; 9 Department of Ecology and Evolutionary Biology, The University of Arizona, Tucson, AZ, United States,; 10 Institut Universitaire Européen de la Mer, Plouzané, France,; 11 American Indian Studies Center, University of California, Los Angeles, Los Angeles, CA, United States,; 12 Department of Marine and Environmental Sciences, Marine Science Center, Northeastern University, Boston, MA, United States

**Keywords:** marine calcification, calcite, aragonite, trace elements, ocean acidification, biomineralization, phylogeny

## Abstract

Elemental ratios in biogenic marine calcium carbonates are widely used in geobiology, environmental science, and paleoenvironmental reconstructions. It is generally accepted that the elemental abundance of biogenic marine carbonates reflects a combination of the abundance of that ion in seawater, the physical properties of seawater, the mineralogy of the biomineral, and the pathways and mechanisms of biomineralization. Here we report measurements of a suite of nine elemental ratios (Li/Ca, B/Ca, Na/Ca, Mg/Ca, Zn/Ca, Sr/Ca, Cd/Ca, Ba/Ca, and U/Ca) in 18 species of benthic marine invertebrates spanning a range of biogenic carbonate polymorph mineralogies (low-Mg calcite, high-Mg calcite, aragonite, mixed mineralogy) and of phyla (including Mollusca, Echinodermata, Arthropoda, Annelida, Cnidaria, Chlorophyta, and Rhodophyta) cultured at a single temperature (25°C) and a range of *p*CO_2_ treatments (ca. 409, 606, 903, and 2856 ppm). This dataset was used to explore various controls over elemental partitioning in biogenic marine carbonates, including species-level and biomineralization-pathway-level controls, the influence of internal pH regulation compared to external pH changes, and biocalcification responses to changes in seawater carbonate chemistry. The dataset also enables exploration of broad scale phylogenetic patterns of elemental partitioning across calcifying species, exhibiting high phylogenetic signals estimated from both uni- and multivariate analyses of the elemental ratio data (univariate: λ = 0–0.889; multivariate: λ = 0.895–0.99). Comparing partial *R*^2^ values returned from non-phylogenetic and phylogenetic regression analyses echo the importance of and show that phylogeny explains the elemental ratio data 1.4–59 times better than mineralogy in five out of nine of the elements analyzed. Therefore, the strong associations between biomineral elemental chemistry and species relatedness suggests mechanistic controls over element incorporation rooted in the evolution of biomineralization mechanisms.

## INTRODUCTION

Calcareous biominerals can possess chemical compositions and morphologies as diverse as the organisms that form them (Lowenstam, 1981; Addadi et al., 2003; Weiner and Dove, 2003). Biominerals form within localized regions, isolated to varying degrees from the external environment. In the marine realm, where seawater is generally the source of the ions for biomineralization, the parent fluid for mineralization is chemically modified from seawater. It is thought that passive leakage of seawater and/or ions to the site of calcification, or active engulfment and vacuolization of seawater and/or transport of ions, may play a role in some organisms, such as corals and foraminifera (Erez, 2003; Gagnon et al., 2012; Venn et al., 2020). To control the composition of this fluid, biomineral constituents (typically cations) are transported into and out of the calcifying fluid by active (ATP-consuming) transport processes or by passive diffusion across membranes and chemical gradients (Simkiss et al., 1986). Organisms also vary widely with respect to the location of biomineral formation, with some such as coccolithophores and echinoderms beginning the process intracellularly, while others, such as molluscs, mineralizing extracellularly onto organic templates (Simkiss et al., 1986; Addadi et al., 2003; Weiner and Dove, 2003). The complex nature of biomineral chemistry, such as the inclusion of organic molecules and metastable mineral phases, has also been suggested to contribute to the variation in the dissolution kinetics of biogenic marine carbonates (Ries et al., 2016).

Marine calcifying organisms typically form either calcite or aragonite, or a mixture of the two calcium carbonate polymorphs. Elemental substitutions for Ca^2+^ in the CaCO_3_ crystal lattice can be predicted by ionic radius and thermodynamic equilibrium (Brand and Veizer, 1980; Menadakis et al., 2009). Elements larger than Ca^2+^ are predicted to be discriminated against in the tight rhombohedral lattice of calcite relative to the orthorhombic lattice of aragonite (e.g., Shen et al., 1991; Gabitov et al., 2011, 2019; DeCarlo et al., 2015; Mavromatis et al., 2018). For calcite, there is also considerable variation in the abundance of Mg^2+^ cations substituting for Ca^2+^ in the crystal lattice. Magnesium is the most abundant secondary element incorporated into marine biogenic CaCO_3_, and secular variation in the Mg/Ca ratio of seawater is hypothesized to be linked to shifts in the dominant CaCO_3_ polymorph found in the geologic record of marine calcifiers (Chavé, 1954; Stanley and Hardie, 1999; Ries, 2010; Drake et al., 2020). Studies on the inorganic precipitation of CaCO_3_ have shown that solution Mg^2+^/Ca^2+^ ratio influences CaCO_3_ polymorph mineralogy, with molar Mg/Ca ratios > 2 favoring precipitation of high-Mg calcite and aragonite and Mg/Ca < 2 favoring low-Mg calcite (Mucci and Morse, 1983; Park et al., 2008; De Choudens-Sánchez and González, 2009; Konrad et al., 2018; Purgstaller et al., 2019; Mergelsberg et al., 2020). Physiological regulation of [Mg^2+^] in the parent fluids for calcification has also been observed within some types of marine calcifiers. For example, *Mytilus edulis* have been observed to actively exclude Mg from their extrapallial fluid relative to seawater (e.g., Lorens and Bender, 1977). Furthermore, the extent of Mg incorporation into calcareous shells appears to influence the incorporation of other elements that substitute for Ca^2+^, such as Sr (Mucci and Morse, 1983). Additionally, some dissolved anions in seawater may substitute for CO_3_^2−^ in the CaCO_3_ lattice (e.g., SO_4_^2−^; Burdett et al., 1989) or may be incorporated by occlusion in the crystal lattice (e.g., HCO_3_^−^; Feng et al., 2006). Cation substitution for Ca^2+^ may also promote these latter two mechanisms of anion incorporation by distorting the crystal lattice (Mucci and Morse, 1983).

In addition to the paleobiological study of the evolution of marine biomineralization, a principal driver of the study of the chemistry of biogenic marine carbonates is the development of tracers for past changes in the ocean. The extensive literature on this topic notably includes the study of the incorporation of Mg and Sr as proxies for ocean temperature, the use of B and Li as proxies for the oceanic carbonate system, the use of Cd and Zn as links to nutrient availability, Ba as a tracer of freshwater input, Na as a tracer of salinity, and the use of Mo, Mn, and U as seawater redox proxies (e.g., Lorens and Bender, 1980; Boyle et al., 1981; Broecker and Peng, 1983; Delaney and Boyle, 1985; Delaney et al., 1989; Rosenthal et al., 1997; Elderfield and Rickaby, 2000; Marchitto, et al., 2002; Russell et al., 2004; Gaetani et al., 2011; Lea, 2013). In addition to these studies exploring relationships between elemental ratios and seawater physicochemical conditions of interest, some also explore drivers underlying so-called “vital effects,” or deviations in biogenic samples from apparent abiogenic equilibrium values (e.g., Weiner and Dove, 2003; Kunioka et al., 2006; Sinclair and Risk, 2006). Each element considered here has its own proposed mechanism of incorporation into CaCO_3_, and we do not attempt to thoroughly review this large and evolving body of work.

Two notable examples are B and U incorporation, as both elements in marine carbonates can be used as tracers for the ocean pH and/or carbonate system (Yu et al., 2007, 2010; Tripati et al., 2009, 2011; Dawber and Tripati, 2012; DeCarlo et al., 2015; Haynes et al., 2019; Guillermic et al., 2020). U and B speciation in seawater are pH dependent; however, for the pH range of the present experiment, the relative abundances of uranium forms does not change (Endrizzi and Rao, 2014), and borate ion (thought to be the main form incorporated within the carbonate) ranges from 24% (highest-pH treatment) to 6% of the total boron (lowest-pH treatment). According to the canonical systematics of B and U, both are incorporated into CaCO_3_ by substitution of CO_3_^2−^ within the crystal lattice, allowing a relatively simple relationship to be derived that relates B/Ca, U/Ca, and δ^11^B in carbonates to ocean pH or DIC species (Hemming and Hanson, 1992; DeCarlo et al., 2015; McCulloch et al., 2017). Although the δ^11^B-based proxy of seawater pH appears to work well in aragonite because aragonite incorporates most B as BO_4_^−^, some recent studies–mainly on experimentally precipitated inorganic CaCO_3_–suggest that the δ^11^B pH proxy may be more complicated to interpret in calcite due to the apparent incorporation of some B as BO_3_^2−^ in that polymorph (e.g., Branson, 2018; Farmer et al., 2019). As the δ^11^B composition of BO_4_^−^ and BO_3_^2−^ in solution is markedly different, this could complicate the use of boron proxies in calcite shells in paleoceanography. In contrast, a recent study found that a diverse range of calcite-precipitating organisms, including high-Mg calcite-producing urchins, yielded δ^11^B values incompatible with significant BO_4_^−^ incorporation, with the possible exception of a coralline red alga species (Sutton et al., 2018; Liu et al., 2020). Furthermore, as many of the investigated species appeared to regulate the pH of the parent fluid for calcification, as indicated by independent measurements (e.g., pH microelectrodes, pH-sensitive dyes), deviations from ideal relationships between B shell chemistry (δ^11^B, B/Ca) and seawater pH are not surprising (Sutton et al., 2018; Liu et al., 2020). These examples highlight that the diverse mechanisms used by marine calcifiers for biomineralization can cause deviations from relationships observed in inorganic precipitation experiments.

Attempts to model the mechanisms driving trace element fractionation between CaCO_3_ and solution have focused on crystal growth rate and processes at the surface of the growing crystal (e.g., Watson, 2004; DePaolo, 2011; Gabitov et al., 2014). Several studies have highlighted the possible importance of Rayleigh fractionation (e.g., Elderfield et al., 1996; Gaetani et al., 2011). However, additional complexity is emerging with the appreciation that many types of organisms, such as molluscs and urchins, produce amorphous precursor phases from which the biomineral ultimately forms via transient and/or metastable phases (Addadi et al., 2003; De Yoreo et al., 2015). The transformation from the amorphous to the crystalline phase may also involve intermediate metastable CaCO_3_ minerals, such as vaterite (e.g., Jacob et al., 2017). Indeed, evidence has emerged that [Mg^2+^], as well as other trace and minor elements, may modulate the transformation of these precursor or and/or metastable phases to crystalline phases (Loste et al., 2003; Purgstaller et al., 2019; Mergelsberg et al., 2020), but with different sensitivities for different trace/minor elements and for different crystalline CaCO_3_ end-products (Evans et al., 2020). Furthermore, it is often assumed that trace elements are hosted in the CaCO_3_ crystal structure, but recent studies of some organisms indicate that this is not always the case. For example, Mg^2+^ in a coral skeletal aragonite was reported to be present in a disordered non-crystalline phase of potential organic affinity (Finch and Allison, 2008). Likewise, a study on ostracods found that Mg^2+^ identified in the shell was not incorporated into the calcite lattice of the shell (Branson et al., 2018).

There is much yet unknown about the controls of trace and minor element incorporation in biominerals, and the literature is strongly focused on exploring this process in taxa that are typically used in palaeoceanographic reconstruction, such as foraminifera, corals, and molluscs. Additionally, previous studies tend to focus on only one to a few species and elemental ratios at a time and use species collected from different geographic locations, impeding direct comparisons between species and their respective geochemistry (e.g., Rosenheim et al., 2005; Gillikin et al., 2006; Dellinger et al., 2018; Piwoni-Piórewicz et al., 2021), emphasizing the need for systematic studies surveying geochemical data across diverse species. Here, we use univariate, multivariate, and phylogenetic analyses to explore a diverse array of 18 species of marine calcifying organisms, including Mollusca, Echinodermata, Arthropoda, Annelida, Chlorophyta, Rhodophyta, and Cnidaria, that were cultured in a controlled laboratory experiment using a single seawater source, a single temperature, and four controlled *p*CO_2_ conditions (Ries, 2009). The calcification responses (Ries et al., 2009), polymorph mineralogies (Ries, 2011), and δ^11^B compositions (Liu et al., 2020) of these cultured specimens has been previously characterized and reported. This sample set represents a unique opportunity to examine broad scale patterns of trace and minor element incorporation in a diverse array of ecologically and economically important marine calcifying organisms.

## MATERIALS AND METHODS

### Culturing Experiments

A detailed description of the culturing experiment and accompanying seawater carbonate chemistry measurements are described elsewhere (Ries et al., 2009), but we briefly summarize key details here. Eighteen species of calcifying marine organisms were cultured for 60 days at 25°C and at four controlled *p*CO_2_ conditions (409, 606, 903, and 2856 ppm) (Table 1). Carbonate system parameters, including salinity, pH_*sw*_, dissolved inorganic carbon (DIC), total alkalinity, and calcite/aragonite saturation state were monitored and the net calcification rates of each specimen was calculated as the percent-change in buoyant weight of the specimen (Supplementary Tables 1, 2). Polymorph mineralogy of the shells (Table 1) was quantified via powder X-ray diffraction as described in Ries (2011).

### Sample Selection

For this study, Li/Ca, B/Ca, Mg/Ca, Zn/Ca, Sr/Ca, Cd/Ca, Ba/Ca, U/Ca, and Na/Ca was quantified in the shells of the 18 calcifying species cultured under four *p*CO_2_ conditions (409, 606, 903, and 2856 ppm), as described Ries et al. (2009). The organisms sampled include the American lobster (*Homarus americanus*), blue crab (*Callinectes sapidus*), gulf shrimp (*Penaeus plebejus*), conch (*Strombus alatus*), limpet (*Crepidula fornicata*), periwinkle (*Littorina littorea*), whelk (*Urosalpinx cinerea*), coralline red alga (*Neogoniolithon* sp.), *Halimeda* green alga (*Halimeda incrassata*), temperate purple urchin (*Arbacia punctulata*), tropical pencil urchin (*Eucidaris tribuloides*), serpulid worm (*Hydroides crucigera*), temperate coral (*Oculina arbuscula*), American oyster (*Crassostrea virginica*), bay scallop (*Argopecten irradians*), blue mussel (*M. edulis*), hard clam (*Mercenaria mercenaria*), and soft clam (*Mya arenaria*; see Table 1 for details). Calcium carbonate powders were collected by scraping the growing edge of shells or skeletons (e.g., molluscs, corals, algae, and serpulid worms), by sampling the tip of individual spines (e.g., urchins), or by pulverizing and homogenizing the entire carapace if it was formed entirely under the experimental conditions (e.g., crabs, lobster, and shrimp). Shell or skeleton formed exclusively under the experimental conditions was identified relative to a ^137^Ba spike that was emplaced in the shells/skeletons at the start of the experiment (Ries, 2011).

### Measurement of Element/Calcium Ratios

Element/calcium ratios were determined for all taxa (Supplementary Table 3) via inductively coupled plasma atomic emission spectrophotometry (ICP-AES). Samples were first cleaned to remove organic matter using an oxidative procedure described in Barker et al. (2003) for the analysis of foraminiferal samples. The sample set contains a number of biomaterials from taxa that have seldom or never been analyzed for X/Ca ratios, including the crustacean carapace, which is a complex biomineral containing chitin and with variable carbonate content. Given the compositional complexity and diversity of the sample set, it was not practical to optimize the cleaning and sample preparation protocol for each species, including assessing potential matrix effects for every species, as this would have been prohibitively costly in terms of time and financial resources. Instead, we used the established protocol of Barker et al. (2003) that is widely used for trace element determinations within biogenic carbonates (e.g., Yu et al., 2005).

Calcium concentrations and X/Ca ratios were determined using a Varian Vista ICP-AES at the University of Cambridge, following the methods of de Villiers et al. (2002) and Barker et al. (2003). Pursuant to the procedures outlined in these studies, samples were diluted to be matrix-matched with standards at a Ca^2+^ concentration of 60 ppm and analyzed in duplicate for consistency. The relative standard deviation (%RSD) of repeated analyses of standards and samples was better than 0.2% for Mg/Ca, consistent with Barker et al. (2003).

All samples were then analyzed on a PerkinElmer Elan DRC II quadrupole inductively coupled plasma mass spectrometer at the University of Cambridge to determine Li/Ca, B/Ca, Zn/Ca, Sr/Ca, Cd/Ca, Ba/Ca, U/Ca, and Na/Ca ratios, with the exception of the crustacea (crabs, lobsters, and shrimp) and *Halimeda* algae due to issues with nebulizer blockage or because elemental ratios were outside of the working range established by the standards. An intensity-ratio calibration was used for determinations, following the procedure of Yu et al. (2005). The relative standard deviation (%RSD) of repeat analyses of standards and samples is better than 1.2% for all elemental ratios, consistent with Yu et al. (2005).

### Apparent Partition Coefficient and Inorganic Partition Coefficient Selection

Apparent partition coefficients (Table 2) of the carbonate biominerals were calculated by dividing the measured element-to-calcium ratios by established corresponding element-to-calcium ratios of seawater (Bruland, 1983), such that:

(1) $$K_{X}=\frac{{\left(X/\text{Ca}\right)}_{\text{mineral}}}{{\left(X/\text{Ca}\right)}_{\text{seawater}}}$$

Inorganic elemental partition coefficients for comparison to biominerals were selected from inorganic calcite or aragonite precipitation studies (See Table 2 and Supplementary Table 6) that employed experimental conditions similar to the culturing experiment of Ries et al. (2009). The inorganic precipitation experiments included in this comparison generally used filtered or artificial seawater and were conducted at or near 25°C and within a pH_*sw*_ range of 7.2–8.5. A summary of the selected partition coefficients for synthetic aragonite, calcite, and amorphous calcium carbonate are presented in Table 2 (see more detailed descriptions of experimental conditions in Supplementary Table 6 and a more detailed review of the literature on element partitioning between inorganic CaCO_3_ and fluid in Supplementary Material section “Description of Inorganic Partition Coefficient Selection”).

### Statistics

All statistical analyses were conducted in R version 3.6.2 (R Core Team, 2019). Outlier testing was conducted using Rosner’s Test as implemented in the *EnviroStat* R package (v0.4–2, Le et al., 2015). This test was used because it was designed to avoid masking effects, which are losses in test power due to larger numbers of outliers being present than being tested for. Outliers were not considered in the analyses and are not illustrated in plots. A complete spreadsheet of the raw data is provided as Supplementary Data Sheet 2.

#### Models and Akaike Information Criterion (AIC)

Models relating X/Ca ratios to seawater carbonate parameters (pH_*sw*_, [CO_3_^2−^]_*sw*_), net calcification rates (Ries et al., 2009), and boron isotope derived pH_*cf*_ (Liu et al., 2020) were conducted by fitting both linear and quadratic models to the data. Both the linear and quadratic models only include sums of terms; thus, both types of models can be considered linear regressions, evaluated, and compared. The regressions were conducted using the glm function in base R, which returned *R*^2^ values and *F*-test statistics. Since both linear and quadratic models were considered linear regressions, *R*^2^ values associated with the regression analyses were comparable and used to model the relationship between the tested variables. The *F*-test within the glm function is a test of overall significance that compares the specified model to a model with no predictors and subsequently generates a *p*-value. Regression models that returned a *p*-value < 0.05 were considered to indicate a statistically significant relationship between the variables of interest. Scatterplots were generated using the *ggplot2*, *ggtext*, and *Rmisc* packages in R and are displayed in Supplementary Figures 10–45 (v3.32, Wickham, 2016; v0.1.1, Wilke, 2020; v1.5, Hope, 2013). Modeled relationships with a *p*-value < 0.05 were outlined in red (Supplementary Figures 10–45). The code used to generate the plots is available on Github with links provided in Supplementary Material section “Phylogenetic Analysis.”

Akaike information criterion (AIC), which ranks models based on their fit and parsimony (Anderson and Burnham, 2004), was used to select between linear or quadratic models for the data. The AIC model that returned the smallest AIC score was selected as the optimal model for describing the relationship between the X/Ca ratio and carbonate chemistry or other measured parameter. The results of the AIC tests are summarized in Tables 3–5, which show the adjusted *R*^2^ and *p*-values for all species exhibiting statistically significant relationships.

#### Generalized Additive Models (GAMs)

We modeled the relationships between eight X/Ca ratios and Mg/Ca ratios using generalized additive models (GAMs) from the *mgcv* package (v1.8–31, Marra and Wood, 2011; Wood, 2017). GAMs are an extension of the generalized linear model family, with the benefit that they have no assumption of a linear relationship between the predictor and response variables, and no assumption of a specific underlying distribution in the data (Hastie and Tibshirani, 1990). Smoothing parameters (penalized regression splines) are introduced and automatically calculated via generalized cross validation (GCV) in the GAM implementation in *mgcv* (Wood, 2011). GAMs are frequently employed in ecological and environmental contexts where the nature of the relationship between predictors and responses is not known *a priori*, and/or a flexible nonparametric model is required.

We tested models with and without the presence of the variable “Phylum” as both an additive and interactive effect to better explore the influence of taxonomy on mineralogy. It should be noted that these analyses are distinct from the phylogenetic analyses described below in section “Phylogenetic Analyses,” as they are not informed by the phylogenetic relationships between species. Models were fit using maximum likelihood, and a likelihood ratio test from package *lmtest* (v0.9–38, Zeileis and Hothorn, 2002) was used to select from among candidate models. In cases where the likelihood ratio test indicated that two models were equally likely, we selected the less complex model. Each final model fit was bootstrapped using 1,000 iterations in package *boot* (v1.3–25, Davison and Hinkley, 1997; Canty and Ripley, 2020) to produce a more robust estimate of the *R*^2^ and is accompanied by a 95% confidence interval around the bootstrapped *R*^2^ (Supplementary Tables 4, 5; see Table 6 for a summary of the best-performing models).

#### Imputation, Hierarchical Clustering, and Ordination

A K-Nearest-Neighbors (KNN) machine learning algorithm was employed to estimate missing values (with a pseudocount of +1 added to place the values on a log-10 scale) in the X/Ca ratio dataset (14.21% of the dataset) using the *impute* R package (v1.64.0, Beretta and Santaniello, 2016; Hastie et al., 2020). For this model, KNN selects an *a priori* neighborhood (3 in this study to get a range of values and not overfit value estimation) and imputes them using a weighted mean of the neighbors where those with the smallest Euclidean distance have more impact on the final estimation. Each “object” represents a species, and its location in the space is determined by each of the log-10 transformed X/Ca ratios. The distance between objects is their straight-line distance in the space. The analysis begins by treating each organism as a separate cluster. Then, the algorithm iteratively merges the two closest (i.e., most similar) clusters until all are merged. The main output of the hierarchical clustering analysis is the dendrogram on the left of Figure 4, which shows the results of the iterative clustering and reveals relationships in X/Ca ratios separated by mineralogy (Figure 4). The resulting dataset was used for ordination and cluster analysis.

Clustering and ordination of elemental ratios by taxon and annotation of phylum was conducted using the *vegan* and *ggplot2* packages (v3.32, Oksanen et al., 2008; v2.5–7, Wickham, 2009). Bray-Curtis dissimilarity (a statistic used to quantify the compositional dissimilarity between two different sites) was calculated for each pairwise imputed elemental ratio by species and subject to complete linkage hierarchical clustering, where the maximum distance between two clusters is identified and the new clusters are merged. This step is repeated until the dataset has agglomerated into a complete dendrogram. The same values were used to model species dissimilarity in ordination space using Non-metric Multidimensional Scaling (NMDS), which was selected because it is rank-based and detects non-linear relationships well. The model collapses information from multiple dimensions and projects this into a lower state (2 dimensions) and iteratively (999 iterations) places objects in ordination space while minimizing distance in projected dimensions as compared to the original dissimilarity matrix.

### Phylogenetic Analyses

Initial phylogenetic trees reflecting the taxonomy for the invertebrate species studied here was constructed with the phyloT application^1^ based on the NCBI taxonomic backbone (Figures 4B–D). NCBI taxonomy IDs used for the studied species were 6706 (*H. americanus*), 6763 (*C. sapidus*), 161926 (*P. plebejus*), 6550 (*M. edulis*), 6565 (*C. virginica*), 6596 (*M. mercenaria*), 6604 (*M. arenaria*), 31199 (*A. irradians*), 387448 (*S. alatus*), 31216 (*L. littorea*), 399971 (*U. cinerea*), 176853 (*C. fornicata*), 7641 (*A. punctulata*), 7632 (*E. tribuloides*), 2071691 (*Neogoniolithon* sp.), 170419 (*H. incrassata*), 1196087 (*H. crucigera*), and 1282862 (*O. arbuscula*). This tree constructed using the NCBI taxonomy was used to initially examine the correlation of species’ geochemistry and mineralogy against taxonomy.

Next, two mitochondrial (COI, cytb) gene regions and a single nuclear (18S) gene region were used to infer the phylogenetic relationships and times of divergence for all the 18 focal species. Supplementary Material section “Methods and Results of Phylogenetic Trees” includes details on tree assembly in BEAST 1.10 (Suchard et al., 2018). The resulting phylogeny is provided in Supplementary Material section “Link to Nexus File for Time-Calibrated Phylogenetic Tree.” The newly assembled time-calibrated phylogeny was first used to test for phylogenetic signal in geochemistry. Phylogenetic signal, defined as the tendency for closely related species to resemble each other more than species selected at random from the tree, is here used as a test for phylogenetic conservatism or inertia in geochemistry (Pagel, 1999). However, the coupling between evolutionary history and patterns of X/Ca divergence between species can also be confounded by alternative variables such as mineralogy (Table 7). Thus, regression models incorporating the newly inferred phylogeny were used to estimate the relative contributions of mineralogy and evolutionary history to differences in geochemistry among species (Figure 5).

Phylogenetic signals in the elemental geochemistry were estimated using both uni- and multivariate approaches. The univariate phylogenetic signal (i.e., for each elemental ratio) was computed using the lambda statistics in the phylosig function in the *phytools* R package (v0.7–70, Revell, 2012). Next, multivariate phylogenetic signals (i.e., across all elemental ratios) were estimated with Pagel’s lambda (Pagel, 1999) using the phyl.pca in *phytools* (Revell, 2009, 2012). For both approaches, lambda estimates range from 0 to 1, with values closer to 1 indicating that differences in elemental ratio(s) among species reflect evolutionary history (phylogenetic conservatism). Finally, the initial dataset was partitioned into five different datasets with variable number of imputed values to determine whether they affect the resulting phylogenetic signal (see details in Supplementary Table 9). First, the phylogenetic signal of an elemental dataset, including all of the elements except Cd/Ca and Na/Ca Second, the original dataset was subsampled for only Zn/Ca, Mg/Ca, Sr/Ca, and Li/Ca data. This dataset did not include any imputed values. Third, since almost half of elemental data for the arthropods were imputed, the shrimp, crabs, and lobsters were excluded from the analyses. Fourth, a dataset with all species was generated that included only Mg/Ca and Sr/Ca data. Fifth, a dataset of all species was generated that included only Mg/Ca, Sr/Ca, and Li/Ca. This set of five additional datasets was used to examine the effects of data imputation on the multivariate phylogenetic signal estimate. Lastly, the relative contributions of the phylogeny and mineralogy to geochemistry patterns among species was estimated using phylogenetic and non-phylogenetic regression models. It should be noted that patterns of divergence between X/Ca ratios and phylogenetic signal can still be explained by differences in polymorph mineralogy. Thus, a set of three regression models were fit for each of the elemental ratios. Each set of regression models included an intercept-only linear regression model (model 1), a linear regression model between the target elemental ratios and mineralogy (model 2), and a phylogenetic regression model between the target elemental ratios and mineralogy (model 3). Non-phylogenetic models were fit using the lm function in the stats R package. Phylogenetic regression models, with lambda values estimated from the data, were fit using the phylolm function in the *phylolm* R package (v2.6.2; Ho et al., 2016). The relative contribution of differences in biogenic carbonate mineralogies among species to elemental ratios among species was estimated by comparing partial *R*^2^-values between non-phylogenetic models (models 1 and 2). Similarly, the relative contribution of evolutionary history to explaining differences in elemental ratios among species was estimated by comparing partial *R*^2^-values between the phylogenetic regression and the linear regression models of elemental ratios versus mineralogy across species and *p*CO_2_ conditions (models 2 and 3). Comparison of partial *R*^2^-values, following Ives (2018), was conducted using the *rr2* R package (v1.0.2, Ives and Li, 2018).

## RESULTS

**FIGURE 1 |:**
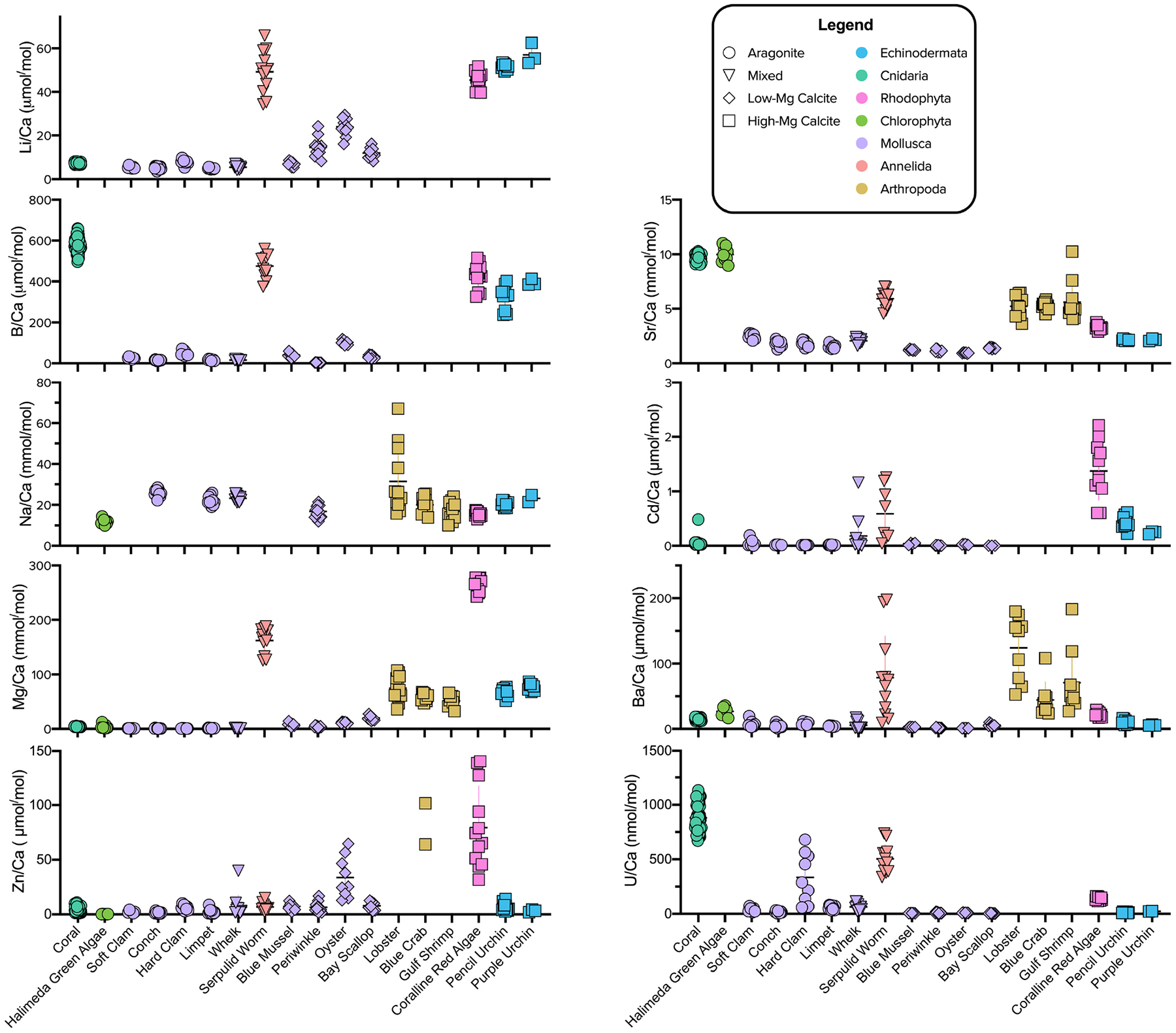
Elemental ratio data across all *p*CO2 culture conditions combined for the invertebrates studied. Color and shape indicate phylum and mineralogy, respectively. The species are organized from left to right according to mineralogy: Aragonite, Mixed Mineralogy, and Calcite. Mixed Mineralogy refers to carbonates with less than 90% of either aragonite or calcite. Differences between species and phyla are typically much greater than the variation within species data that could be attributed to varying seawater conditions. Note some elemental ratios are not displayed to avoid scale compression or due to low confidence in their analytical accuracy (e.g., arthropod Zn/Ca; see section “Materials and Methods”).

The reported X/Ca ratios for each species represent means of the measured data across the *p*CO_2_ culture conditions (Supplementary Table 3). These mean values were used for the multivariate and phylogenetic analyses described below because the range of X/Ca values for a given species across *p*CO_2_ treatments is relatively small compared to the differences in mean X/Ca of different species. For example, the pencil urchin, *E. tribuloides*, exhibits Li/Ca and Mg/Ca values of 50–53 μmol/mol and 60–78 mmol/mol across *p*CO_2_ treatments, respectively, whereas the oyster, *C. virginica*, exhibits values of 15–30 μmol/mol and 9.5–13 mmol/mol, respectively (Figure 1).

### Element/Calcium Ratios Amongst Diverse Marine Calcifiers

As expected, Mg, Sr, and Na were the most abundant elemental substitutions across all mineralogies (Figure 1 and Supplementary Table 3; Reeder, 1983; Sugawara and Kato, 2000; Allison et al., 2001; Iglikowska et al., 2016). The calcite in crustaceans’ carapaces and urchins’ spicules exhibits Mg/Ca in the range of 50–100 mmol/mol and the mixed mineralogy serpulid worm and calcitic coralline red algae exhibit relatively high Mg/Ca in the range of 100–300 mmol/mol, while the aragonite-producing species were low in Mg/Ca. Molluscs exhibited low Mg/Ca, regardless of the CaCO_3_ polymorph produced. Sr/Ca was highest in the range of 9–12 mmol/mol in the aragonite-forming coral and green algae. Strikingly, mollusc CaCO_3_ of all polymorphs was relatively low in all elemental ratios compared to other species, except for Li/Ca compared to the temperate coral and Na/Ca compared to most other species. Of the other elements, Li/Ca and B/Ca was notably higher in organisms that formed high-Mg calcite, including the serpulid worm, which produces a worm tube of aragonite and high-Mg calcite (see more analyses below in section “Relationships Between Mg/Ca and Other Elemental Ratios”). B/Ca was also high in the aragonite coral skeleton. The coralline red algae and serpulid worm generally had comparatively high levels of all measured elements compared to the other species. Amongst the aragonite producing species, the coral was noticeably different in that it exhibited much higher B/Ca, Sr/Ca, and U/Ca. The *Halimeda* algae exhibited elemental ratios similar to those of the coral.

### Relationships Between Mg/Ca and Other Elemental Ratios

**FIGURE 2 |:**
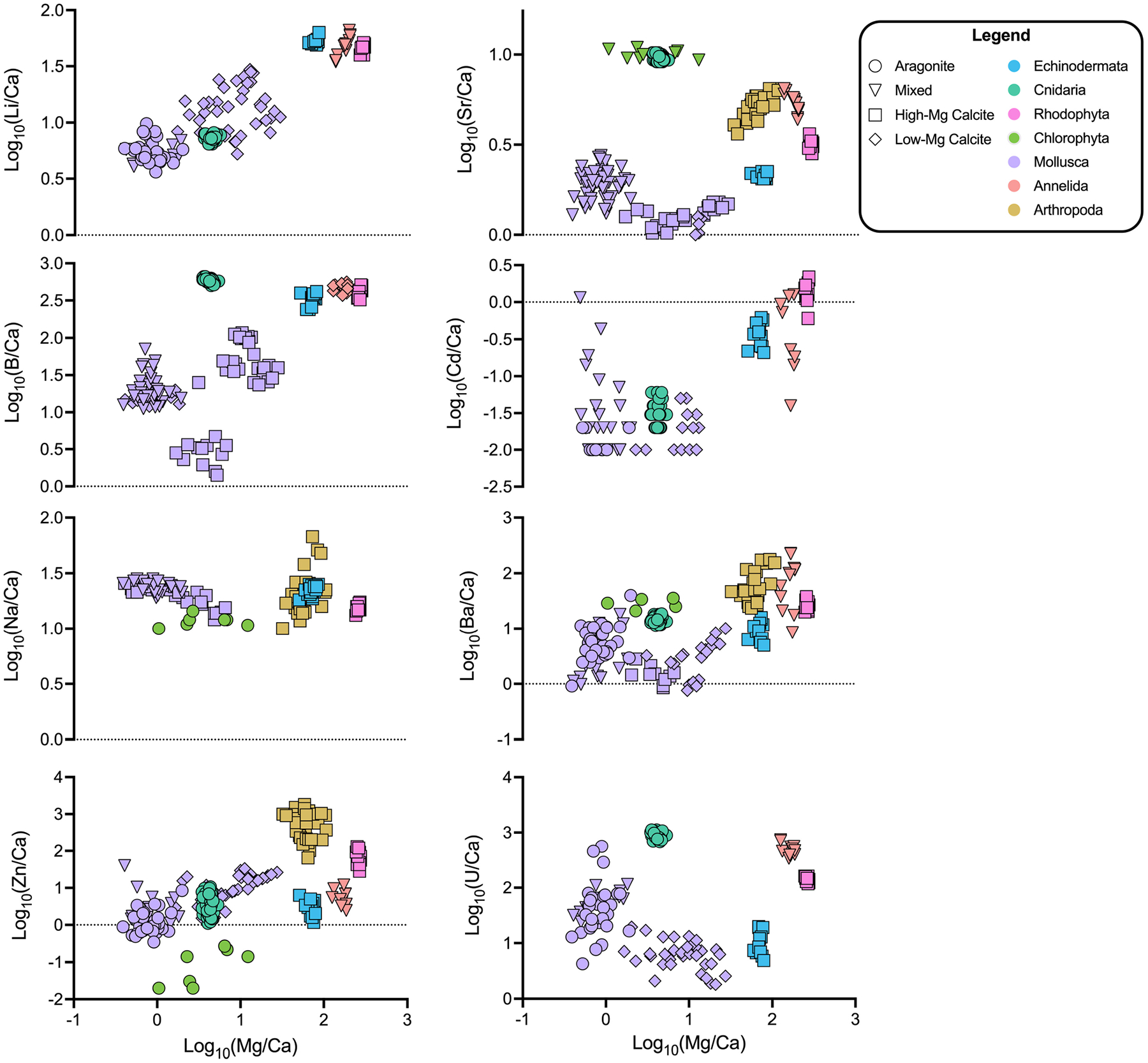
Elemental ratio data plotted against Mg/Ca (log10-transformed values are shown. Color and shape indicate phylum and mineralogy, respectively. Generalized additive models (GAMs) of this data show a number of significant relationships between elemental ratios and Mg/Ca, explaining 0–71% of the variance in the data (Supplementary Table 4). Grouping the organisms by phylum or mineralogy substantially improves model performance, and the best-performing models consider either or both grouping variables (38.70–99.00% of variance explained; Table 6).

Generally, higher incorporation of Mg in the biomineral lattice appears to be linked to higher incorporation of other elements (Figure 2). For example, for Li/Ca, the molluscs and coral group together with lower Mg content (Li/Ca: 6.5–25 μmol/mol; Mg/Ca: 0.5–6 mmol/mol), while the echinoderms, serpulid worms, and coralline red algae are relatively enriched in both Mg and Li (Li/Ca: 39–60 μmol/mol; Mg/Ca: 60–275 mmol/mol). Potential increases in Zn/Ca with Mg incorporation are less clearly resolved.

In this X/Ca-Mg/Ca space, the data appear to group by phylum, so generalized additive models (GAMs) were used to explore the influences of phylum and mineralogy on elemental clustering. The final GAMs explain between 26.4 and 99% of the variation in the elemental ratio of interest, with model selection indicating a preferred final model with phylum, or a combination of phylum and mineralogy, for seven of the eight elements (see Table 6 for final models; see Supplementary Table 4 for complete list of candidate models; see in Supplementary Table 5 for likelihood ratio test results; see Supplementary Figure 9 for final model components). Models that only consider Mg/Ca explain up to 71.9% of the variation in the element of interest but were in no case preferred by model selection. In seven of eight cases, including mineralogy with Mg/Ca substantially improves the models and explains between 16.6 and 95.6% of the variation in the element of interest. Cd/Ca is the only case where the model including only mineralogy was preferred. Six of the eight models were improved by including phylum, explaining up to 98.7% of the variation. All final models produced normally distributed residuals (Supplementary Figures 1–8). Overall, we find evidence that both mineralogy and phylum are key predictors of elemental ratios in the study organisms. Please note that these models consider the phylum that were used to classify the samples (i.e., Rhodophyta, Chlorophyta, Cnidaria, etc.); the models are not informed by the phylogenetic relationships between species. The results of the phylogenetically informed analyses are described below in section “Phylogenetic Signal and Regression Analyses.”

### Comparison of Inorganic Carbonate Element Partitioning to Marine Biominerals

**FIGURE 3 |:**
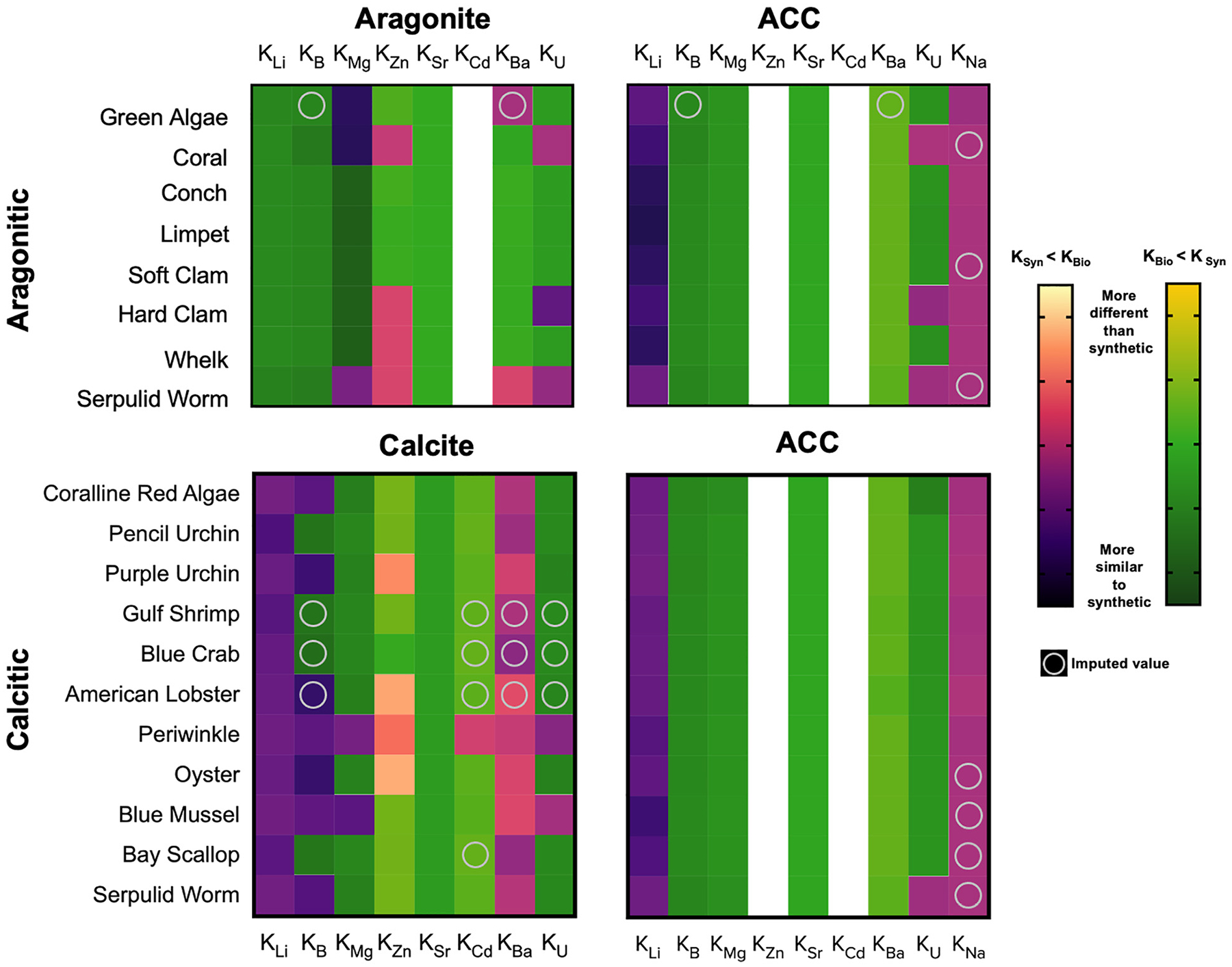
Heatmaps displaying the log10-transformed differences between apparent partition coefficients and published partition coefficients from inorganic mineral precipitation experiments (See Table 2 for values; Supplementary Table 5 for experimental conditions and citations for inorganic values). The apparent elemental partition coefficients calculated for the cultured species studied typically do not align with values derived from synthetic minerals, and species belonging to the same phyla appear to exhibit similar patterns. The warm color gradient (i.e., the left, yellow-black gradient) represents when biogenic carbonates are enriched in an element relative to the published synthetic values (*K*Synthetic < *K*Biogenic). Alternatively, a green-yellow color gradient (i.e., the right gradient) represents when biogenic carbonates are depleted relative to the published synthetic values (*K*Biogenic < *K*Synthetic). White pixels indicate that no published, synthetic values for that element were found. Circles within pixels denote imputed values (See section “Imputation, Hierarchical Clustering, and Ordination” and Figure 4 for details). Apparent partition coefficients were calculated by dividing the measured and imputed elemental ratios of the biogenic carbonates by established seawater minor and trace element concentrations (Bruland, 1983). The top row of panels presents the aragonitic organisms, while the bottom panels represent the calcitic organisms. The label above each panel denotes the inorganic, synthetic partition coefficients being compared to (i.e., aragonite, calcite, and amorphous calcium carbonate; ACC). The color of the pixels was determined by log10(| *K*X, Biogenic – *K*X, Synthetic|). The absolute value is used since y cannot be less than 0 in log10(y).

Patterns of elemental partition coefficients (*K*_*x*_) observed here are of course similar to those observed for the X/Ca data. However, calculating the apparent partition coefficients allows for comparison to other cultured biogenic samples and published partition coefficients derived from inorganic synthesis of calcite, aragonite, and amorphous calcium carbonate (ACC; Table 2 and Figure 3). Though some apparent partition coefficients align with those of inorganically precipitated CaCO_3_ minerals (e.g., *K*_*Li*_ for aragonitic organisms and ACC; *K*_*Li*_ for calcitic organisms and calcite), there are many that are relatively enriched or depleted with respect to those observed for inorganic precipitates. Positioning relative to the inorganic partition coefficients appears to be consistent amongst organisms of the same phylum regardless of mineralogy, as observed, for example, in the mollusca.

The molluscs are relatively depleted with respect to most of the measured elements compared the other organisms, while the coralline red algae and echinoderms are relatively enriched with respect to these elements. For *K*_*Li*_, the coral and the molluscs, regardless of mineralogy, exhibited values aligned with the published inorganic partition coefficients of ACC. The apparent *K*_*B*_ values of the calcitic organisms all lie closest to the published partition coefficient for calcite (Figure 3 and Table 2). The apparent *K*_*B*_ of aragonitic coral (0.014 ± 0.0009) is depleted relative to inorganic aragonite (0.02–2.48). The apparent *K*_*B*_ of the serpulid worm tube (0.0117 ± 0.0014) falls between the published values for inorganic calcite (1.4E-6–4.14E-3) and inorganic aragonite (0.02–2.48), consistent with its mixed mineralogy.

*K*_*Zn*_ values spanned the greatest range across the organisms, with the crustaceans exhibiting values >200. Only the coralline red algae exhibited *K*_*Zn*_ that was comparable to that of the crustacea, ranging from approximately 100 to 200. With the exception of the calcitic Eastern Oyster, which has an apparent partition coefficient similar to that of inorganic calcite, the remainder of the organisms sampled align with the *K*_*Zn*_ published for inorganic aragonite.

Surprisingly, apparent *K*_*Sr*_ for all species studied were lower relative to published values for inorganic precipitates of corresponding carbonate polymorph (Schöne et al., 2010). The highest apparent *K*_*Sr*_ for organisms were calculated from the aragonitic species of coral and *Halimeda* algae sampled, which range from 0.001 to 0.00125. The next highest values were calculated for the serpulid worm and the arthropods—approximately half of that of the aragonite species—which possess shells of mixed mineralogy and high-Mg calcite, respectively. The *K*_*Sr*_ of the coralline red algae (4E-4 ± 0.2), molluscs (1.8–2.9E-4), and echinoderms (2.0–3.0E-4) are even lower.

All of the molluscs, as well as the aragonitic coral and *Halimeda* algae, have apparent *K*_*Mg*_ similar to that of synthetic aragonite (4.72E-4). The high-Mg calcite arthropods and echinoderms are enriched relative to the values for published inorganic calcite (2.66E-2), while the serpulid worm is comparable to value for inorganic calcite. Coralline red algae high-Mg calcite exhibits the highest *K*_*Mg*_ at approximately 0.06.

There were no published partition coefficients for Cd in synthetic aragonite. The published *K*_*Cd*_ for synthetic calcite (18.5) overlaps with the highest and lowest ends of the respective ranges of apparent *K*_*Cd*_ calculated for the serpulid worm (9.78) and the coralline red algae (22.9). The coral, molluscs and echinoderms are all extremely depleted with respect to Cd relative to synthetic calcite.

The apparent *K*_*Ba*_ values calculated for the crustacea and the serpulid worm are higher than the other species and published partition coefficients for synthetic CaCO_3_ minerals, though their values range widely from 3 to 20. The lowest limit of this range as well as both the *Halimeda* and the coralline red algae appear to align with the *K*_*Ba*_ measured for synthetic ACC. The echinoderms, which produce high-Mg calcite biominerals, provide an apparent *K*_*Ba*_ consistent with that derived from synthetic calcite experiments. The apparent *K*_*Ba*_ of the mollusk species appear to be consistent, regardless of mineralogy, and lie between the published *K*_*Ba*_ values for synthetic calcite and aragonite. The values calculated for coral fall between the published values for synthetic calcite and ACC.

The highest apparent *K*_*U*_ are calculated for the coral and the serpulid worm, ranging from 0.5 to 0.75 and 0.25 to 0.40, respectively, and are both enriched relative to the published *K*_*U*_ values for synthetic ACC, calcite, and aragonite. The molluscs, regardless of mineralogy, and the echinoderms partition U similarly to synthetic calcite, except for the mixed mineralogy hard clam, which has an average calculated *K*_*U*_ similar to values published for synthetic aragonite. Coralline red algae appear to partition U similarly to synthetic ACC.

### Imputed Values and Hierarchical Clustering

**FIGURE 4 |:**
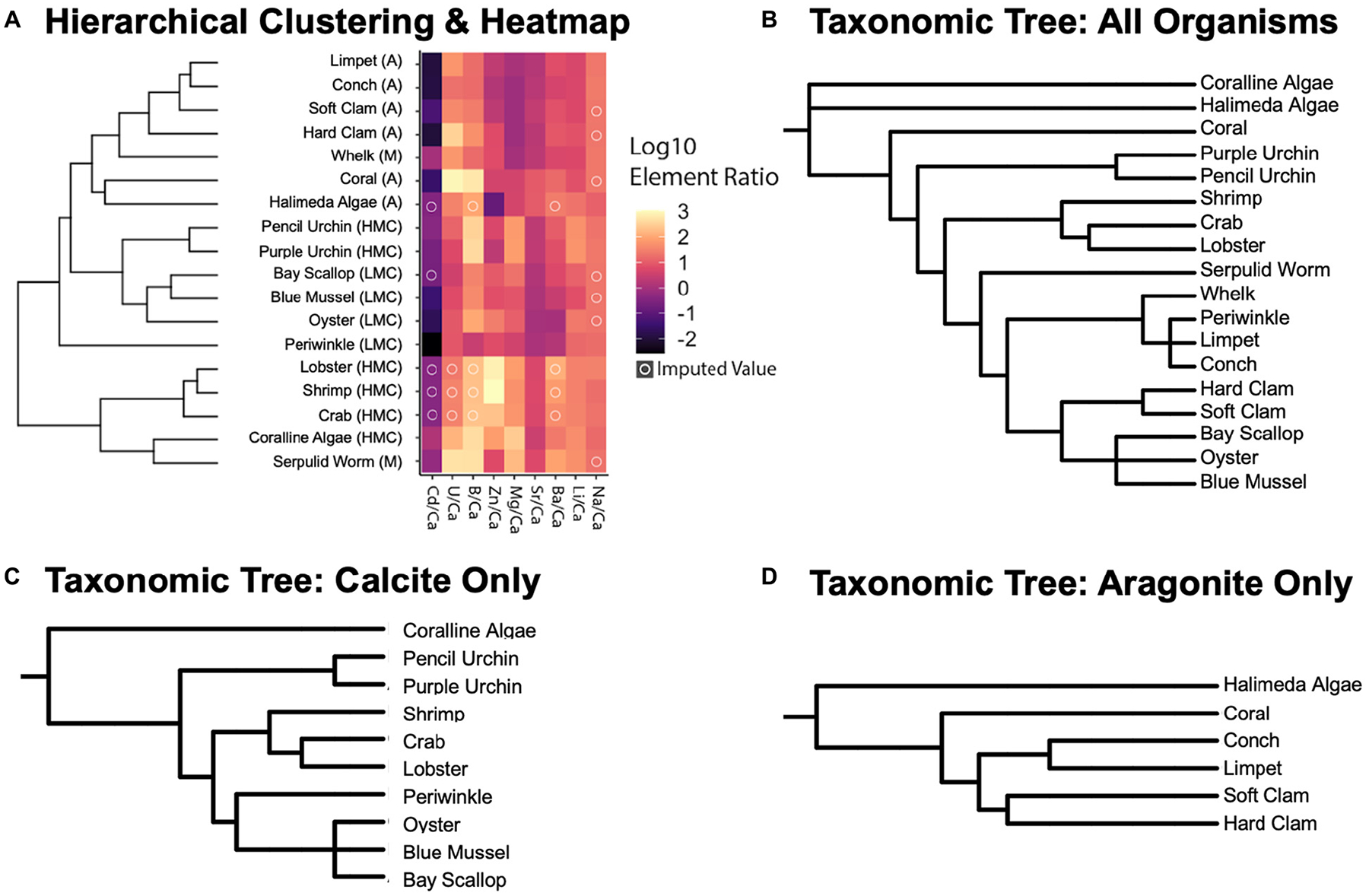
**(A)** Hierarchically clustered (Bray-Curtis dissimilarity) heatmap of log10 values of element to calcium ratios separated by species. Letters in ellipses correspond to organism biomineralogy: A, aragonite; HMC, high Mg calcite; LMC, low Mg calcite; and M, mixed. Lighter colors indicate higher values from log10-transformations of elemental ratios, and an “O” on top of a pixel indicates that the value was imputed using a K-Nearest Neighbors model estimation (see section “Materials and Methods” for details). **(B–D)** Taxonomic trees of the studied organisms based on genetic similarity/difference for all, calcitic, and aragonitic species, respectively. Taxonomic trees were built using the phyloT application based on the NCBI taxonomic backbone (see section “Phylogenetic Analyses”; Letunic and Bork, 2019). Qualitative similarities between the resulting dendrogram of the clustering analysis and the mineral-specific taxonomic trees suggest that taxonomic patterns arise in the elemental ratio measurements but are potentially convoluted by mineralogical effects.

**FIGURE 5 |:**
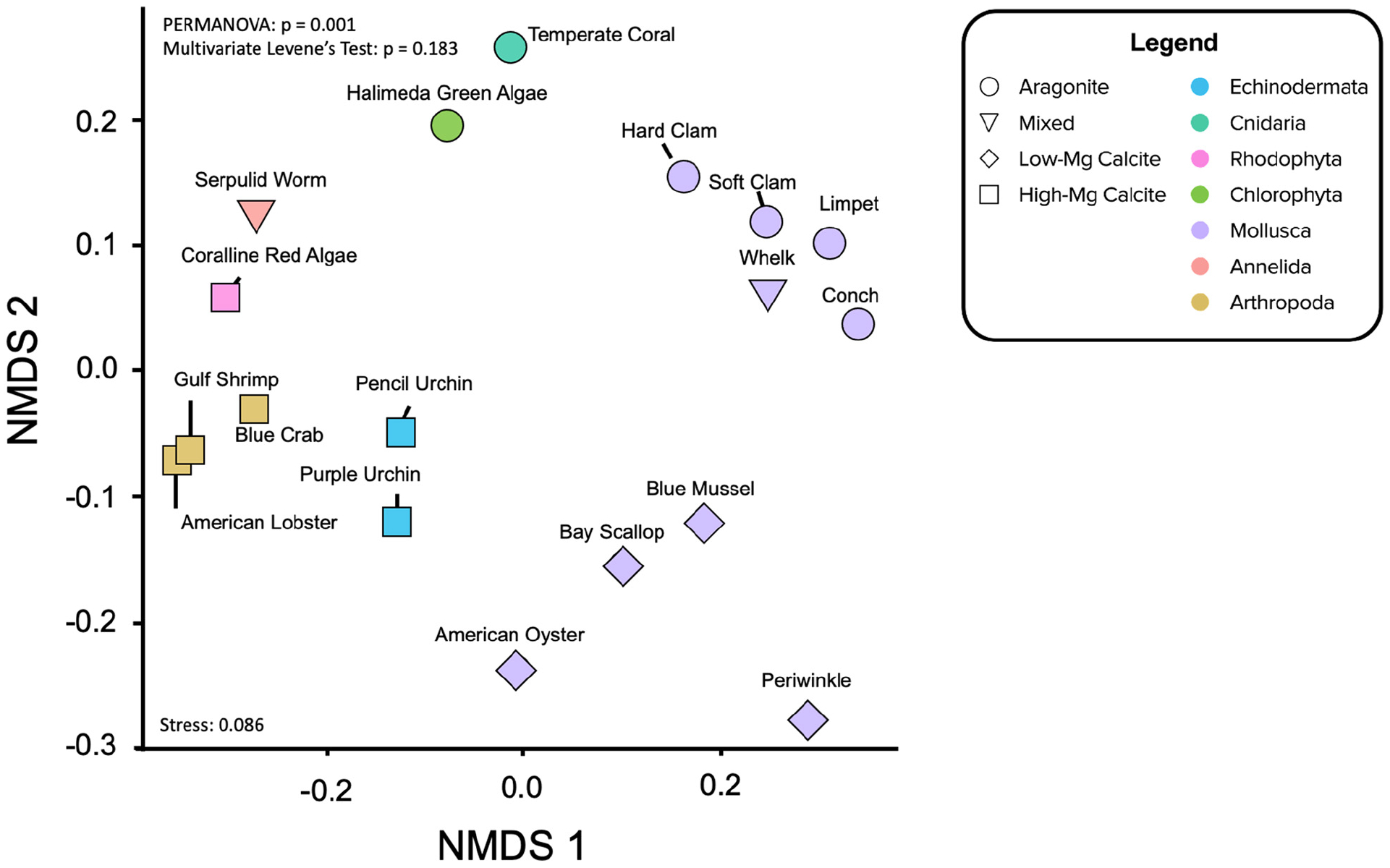
Non-metric Multidimensional Scaling (NMDS) ordination of the elemental ratio data projected to two-dimensions and colored by taxon phylum. Shape denotes mineralogy. PERMANOVA determined whether clustering by phyla is significant (*p* = 0.001). Multivariate Levene’s test of homogeneity (*p* = 0.183) and the stress value for this analysis (0.086) are reported. Visualizing the elemental ratio data in this way further illustrates the relative influences of mineralogy and phylogeny and potentially provides a framework for interpreting diagenetic effects of fossil carbonates.

**FIGURE 6 |:**
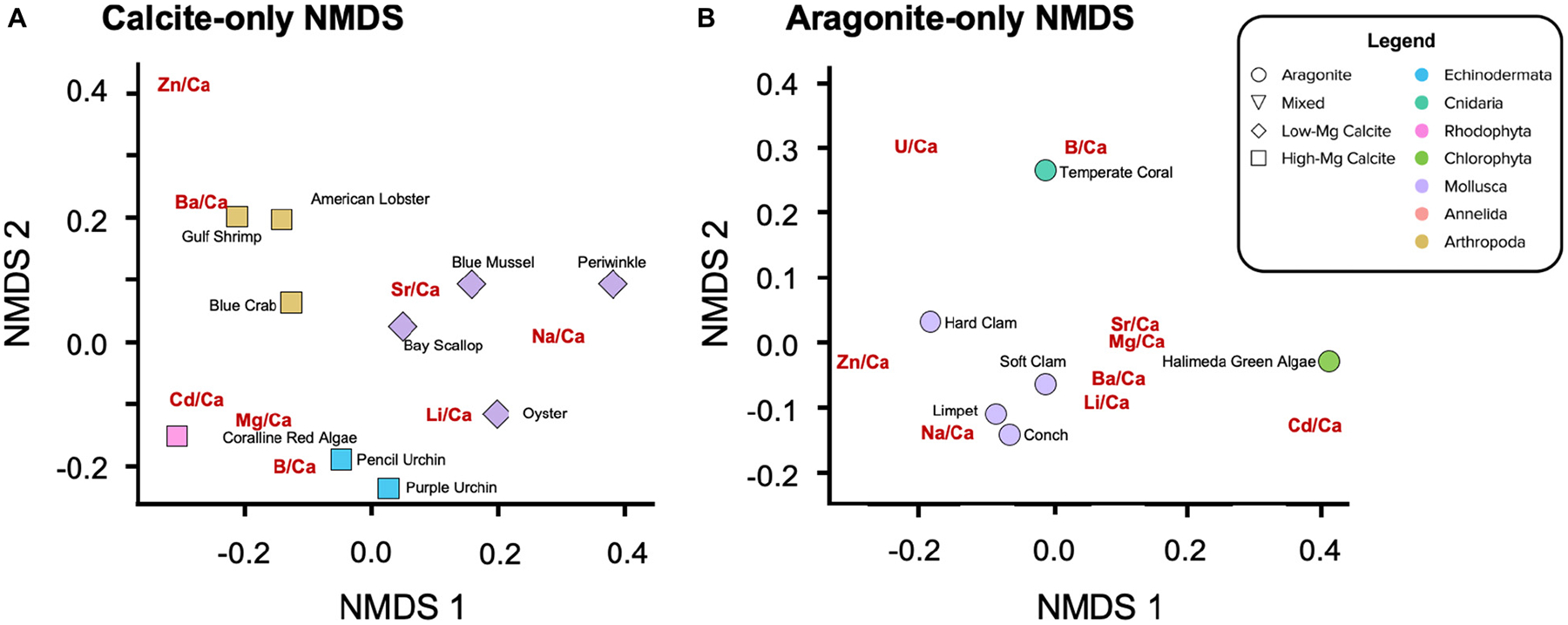
NMDS ordination of elemental ratios for **(A)** calcitic and **(B)** aragonitic organisms only, which are separated by species. The elemental ratios themselves are also represented in the ordination space. The positioning of the elemental ratios depends on the apparent level of incorporation in the respective biogenic carbonates; the closer to an organism the trace element ratio, the higher the level of incorporation of that element relative to the other organisms represented in the space. Considering only one mineralogy at a times excludes the influence of mineralogical differences. The color of a point denotes phyla. **(A)** Similar to Figure 4B, PERMANOVA determines significant clustering of points by phylum (*p* = 0.003). The stress value for this analysis is 0.035. **(B)** Similar to Figure 4B, PERMANOVA determines significant clustering of points by phylum (*p* = 0.136). The stress value for this analysis is 8.84E-5.

Some of the analyzed specimens were missing elemental ratios (14.2% of the dataset). These missing data were imputed using the data of similar points in the space (see Methods 2.5.3 for details; see imputed results in Supplementary Table 3) in order to facilitate multivariate and phylogenetic analysis of the dataset. These mean and imputed values for elemental ratios are used for the hierarchical clustering (Figure 4A) and phylogenetic signal analyses (Table 7 and Supplementary Table 8), as well as for the non-metric multidimensional scaling ordinations (NMDS; Figures 5, 6A,B) described below.

When compared to the taxonomic trees (Figures 4B–D), the hierarchical clustering results of the elemental ratio data (Figure 4A) appear to recapitulate the mineralogically specific taxonomic trees (Figures 4C,D). When including species of differing polymorph mineralogy, the similarities between the clustering analysis and the taxonomic tree (Figure 4B) seem to be confounded by polymorph mineralogy. Parsing out the confounding effects of mineralogy from the effects of phylogeny is explored below.

### Phylogenetic Signal and Regression Analyses

Uni- and multivariate analyses of phylogenetic signal based on the Pagel’s lambda statistics, reflecting the extent to which closely related species show similar X/Ca ratios, generally indicate a strong coupling between the phylogenetic tree and patterns of elemental ratios between species. While a Pagel’s lambda value of 0 for X/Ca ratios would indicate that closely related species are more likely to differ in X/Ca ratios than distantly related species (Pagel, 1999), the multivariate analyses of all nine X/Ca ratios recovered an estimated lambda of 0.89–0.99 (Table 7; range based on alternative datasets), indicating that closely related species tend to have similar X/Ca ratios. However, patterns of congruent evolutionary history and X/Ca ratios are not generalizable across all X/Ca ratios. For instance, three out of the nine X/Ca ratios examined in this study had phylogenetically decoupled patterns of change in ratios between species (Li/Ca, Zn/Ca, and Ba/Ca; Table 7).

We also explored the potential impact of the imputed values on the phylogenetic signal results by determining Pagel’s lambda with subsets of the data that minimize the number of imputed values (e.g., 0.938 for all X/Ca ratios except Cd/Ca and Na/Ca; 0.968 for all species except Arthropods) and found no substantial effects of missing data on estimate of phylogenetic signal (Supplementary Table 9). Furthermore, the impact of using a lower number of X/Ca was assessed through determining Pagel’s lambda using subsets of two, three, and four elemental ratios (Table 7). Pagel’s lambda remains high (0.925 for multivariate phylogenetic signal across Mg/Ca, Sr/Ca; 0.803 for Mg/Ca; 0.835 for Sr/Ca; Table 7). However, when an elemental ratio with no apparent phylogenetic signal is included in the analysis, lambda slightly decreases (0.898; Table 7). Thus, the analyses suggest that for some, but not all, of the examined X/Ca ratios, phylogenetic closeness is potentially a major driver of differences in X/Ca values amongst species.

**FIGURE 7 |:**
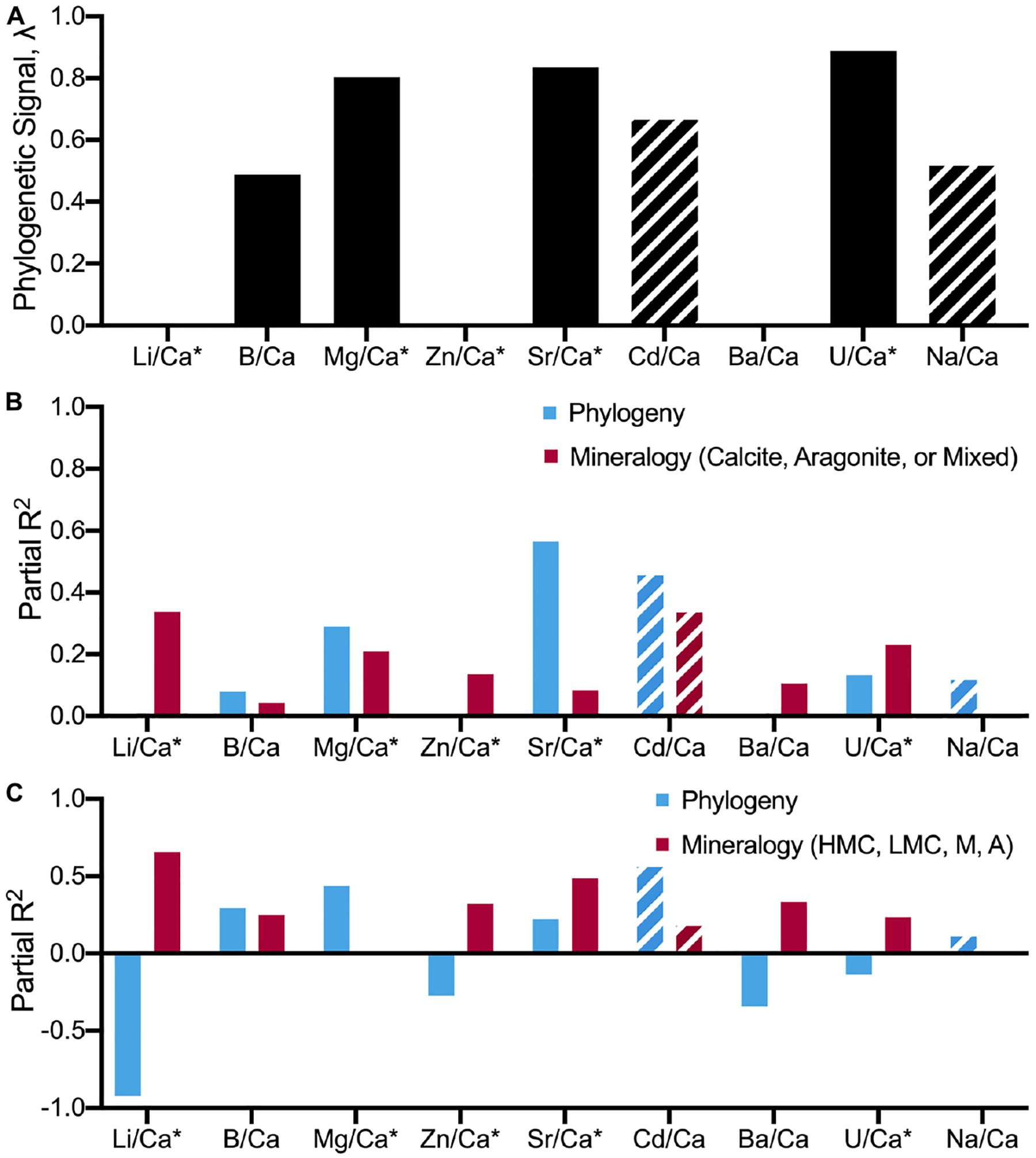
Estimated relative contributions of phylogeny and mineralogy on X/Ca ratios using phylogenetic and non-phylogenetic models on the full species-level dataset. **(A)** Bar graphs illustrating the phylogenetic signal, λ, **(B,C)** relative contributions of evolutionary history (phylogeny) and mineralogy on differences in X/Ca ratios among 18 focal species. Partial *R*^2^s for each predictor within each given element-to-calcium ratio follow Ives (2018) and Ives and Li (2018). Full results are provided in Supplementary Table 9. “*” denotes elemental ratios that have three or less values imputed. Mg, Sr, Li, and Zn datasets have zero imputed values. Striped bars denote elemental ratios (Cd and Na) that consider five or more imputed values, potentially influencing the resulting λ and *R*^2^. The difference between graphs **(B,C)** are how mineralogy was defined for the analysis: **(B)** Mineralogy is categorized as whether an organism produces calcite or aragonite, or a mixture of the two; **(C)** Mineralogy is broken down further, categorizing mineralogy as HMC, high-Mg calcite; LMC, low-Mg calcite; A, aragonite; or M, mixture (Table 1). The high phylogenetic signals and high partial-*R*^2^ Phylogeny values deconvolve phylogenetic and mineralogical impacts on the elemental ratios, highlighting the importance of considering phylogeny when assessing elemental ratio data.

Further, regression analysis was used to examine whether interspecific differences in X/Ca ratios are better explained by phylogeny or differences in mineralogy. As shown above, phylogenetic signal varies between X/Ca ratios (Table 7), so the effects of phylogenetic history relative to mineralogy are expected to vary amongst elemental ratios. Phylogenetic and non-phylogenetic models were used to resolve the relative contributions of evolutionary history and mineralogy to explaining patterns of X/Ca ratios between species. When classifying mineralogy as calcite, aragonite, or mixed, phylogeny was 1.4–59 times better at explaining variance in X/Ca ratios than mineralogy in five out of the nine X/Ca ratios (i.e., B/Ca, Mg/Ca, Sr/Ca, Cd/Ca, and Na/Ca; Figure 7 and Supplementary Table 9). However, when mineralogical classification is broken down further (high-Mg calcite, low-Mg calcite, aragonite, and mixed), the amount of variance explained by phylogeny decreases to 1.3–15.7 times better in four out of the nine X/Ca ratios, demonstrating the importance of distinguishing between low-Mg and high-Mg calcites. Also notable is that in all but one of the X/Ca ratios (U/Ca), high phylogenetic signal was coupled with higher explanatory effects of phylogeny relative to mineralogy. Overall, these phylogenetic analyses suggest that elevated phylogenetic signals generally indicate that differences in X/Ca ratios are better explained by phylogenetic closeness, not mineralogy. Thus, the elevated phylogenetic signal recovered across all X/Ca ratios using multivariate analyses reflects the major contributions of phylogeny to generating extant patterns of X/Ca ratios amongst species.

Finally, mollusc elemental data were used to examine the relative contributions of mineralogy and phylogeny to explaining differences in X/Ca ratios between species within the same phylum. Regression analyses were conducted comparing the relative contributions of mineralogy and phylogenetic history using phylogenetic and non-phylogenetic models. The mollusk dataset includes four species assigned to the calcite category, four assigned to the aragonite category, and a single species to the mixed mineralogy category. It should be noted that the limited sample size may compromise the calculations assessing phylogenetic signal at the class level presented below. Regression models run for X/Ca ratios with high phylogenetic signals (B/Ca, Mg/Ca, and Sr/Ca; Table 7) found that for two of the three X/Ca ratios examined within molluscs, phylogenetic history better explains patterns of divergence in X/Ca ratios than mineralogy. Phylogeny is about two times better than mineralogy (partial *R*^2^_*phylogeny*_ = 0.239 vs. mineralogy = 0.097) in predicting B/Ca. Similarly, phylogeny explains nearly two times more variance in Mg/Ca than mineralogy (partial *R*^2^, phylogeny = 0.60 vs. mineralogy = 0.33). For Sr/Ca, both phylogeny and mineralogy explain a similar fraction of differences in ratios between species (partial *R*^2^, phylogeny = 0.632 vs. mineralogy = 0.65). Overall, results within molluscs for X/Ca ratios with high phylogenetic signal indicate that phylogeny is generally a better predictor of differences in X/Ca ratios between species than mineralogy. Nevertheless, as indicated above, these results were potentially affected by the limited sample size and should be re-examined in future studies including more mollusc species. This analysis of a mollusc-only dataset reaffirms that, for specific X/Ca with high phylogenetic signal, differences in X/Ca between species largely reflect phylogenetic closeness, not mineralogy.

### Non-metric Multidimensional Scaling (NMDS) Ordination

Non-metric Multidimensional Scaling ordinations project the all of X/Ca ratios, including the imputed values, project in two dimensions (Figures 5, 6). As opposed to Euclidean distance, which is the distance computed for the hierarchical clustering analysis, NMDS uses rank-based ordering and is better suited for detecting non-linear relationships. The model iteratively collapses the nine dimensions of X/Ca ratios onto two dimensions, seeking to minimize the distances between species.

Not surprisingly, there is a split with the dataset between the calcitic and aragonitic organisms, with the 50HMC:50A serpulid worm occupying an intermediate position between the groups (Figure 5). Within mineralogy, however, species cluster according to phylogenetic relationships (Figure 5) much more strongly than in the hierarchical clustering analysis. NMDS ordination of all organisms shows statistically significant clusters of X/Ca ratios by phylum (i.e., for species of same mineralogy), where the ratios within phylum show equal variances across organisms (PERMANOVA: *p* = 0.001; permutational multivariate Levene’s test: *p* = 0.183). For example, the bivalve molluscs fall close together, as do the arthropods and echinoderms. The aragonitic *Halimeda* green algae and temperature coral aragonitic species lie proximal, as do the high-Mg calcite producing coralline red algae and the serpulid worms of mixed mineralogy.

Separating the calcite-forming (Figure 6A) and the aragonite-forming (Figure 6B) organisms removes the role of mineralogy while revealing rank-based positioning of the X/Ca ratios, themselves. In the calcite-only NMDS, significant clustering of organisms by phylum is also observed (Figure 6A; PERMANOVA: *p* = 0.003), along with variation within phyla. The X/Ca ratios position themselves in the ordination space with a similar multivariate structure as the organisms; they lie closest to organisms that incorporate them the most. Of the arthropods, Ba/Ca has the most similar data structure to the American lobster and Gulf shrimp. Li/Ca, Na/Ca, and Sr/Ca in the calcitic organisms have data structures similar to those elements in the calcitic molluscs, although there is some variation amongst them. Also, Li/Ca in addition to B/Ca in the calcite ordination to have data structures similar to those elements in the echinoderms. The coralline red alga exhibits the most distinct data structure, exhibiting high ratios of Mg/Ca, Cd/Ca, and U/Ca. Zn/Ca in the calcite ordination does not appear to have a data structure similar to that of any of the other calcitic organisms. In the aragonite-only NMDS, significant clustering by phyla is again observed (Figure 6B; PERMANOVA: *p* = 0.136). The temperate coral and the *Halimeda* green algae have data structures more similar to B/Ca and Cd/Ca, respectively. The aragonitic molluscs, with the exception of the hard clam, exhibit relatively high Na/Ca, Li/Ca, and Ba/Ca. Also of note is the clustering of Sr/Ca, Mg/Ca, Ba/Ca, and Li/Ca for the aragonitic species, potentially indicating similar mechanisms of partitioning.

### Relationships Between Elemental Ratios, Carbonate Chemistry, and Other Measured Parameters

Relationships between the elemental data and a number of the measured experimental parameters were explored via linear and quadratic regressional analysis, including net calcification rate (Ries et al., 2009), culture water carbonate ion concentration ([CO_3_^2−^]), culture seawater pH (pH_*sw*_), and δ^11^B-derived internal calcifying fluid pH (pH_*cf*_; Liu et al., 2020; Supplementary Figures 10–45). If both linear and quadratic fits were significant, Akaike Information Criterion (AIC) was used to compare and identify the best fit (Tables 3–5).

A total of 16 species displayed at least one significant relationship between an X/Ca ratio and seawater [CO_3_^2−^]. Nine species exhibited more than four significant linear or quadratic relationships, including the coral, coralline red algae, hard clam, limpet, oyster, pencil urchin, serpulid worm, soft clam, and whelk. The pencil urchin exhibited the most significant relationships between the measured elemental ratios versus the carbonate chemistry and other measured parameters. Of the elements, Sr/Ca had the most significant relationships, while only one significant relationship was identified for Na/Ca between both linear and quadratic fits for all species versus the carbonate chemistry and other measured parameters.

Net calcification rate, as determined by the buoyant weight method described in Ries et al. (2009), has 24 significant relationships with X/Ca ratios across all species. The species with the most significant relationships with changing net calcification rate was the hard clam, and the elements most frequently correlated with calcification rate across species are Sr and Ba. Sr/Ca significantly increases with increasing net calcification rate in a few molluscs, including the soft clam, blue mussel, and limpet. The aragonitic molluscs, the soft clam and the limpet, exhibit Sr/Ca that increases linearly with higher net calcification rates (*R*^2^ = 0.74, *p* = 0.0002; *R*^2^ = 0.342, *p* = 0.02), while the calcitic blue mussel Sr/Ca exhibits a parabolic variation with net calcification rate (*R*^2^ = 0.452, *p* = 0.01). The pencil urchin Sr/Ca exhibits an inverse parabolic pattern with respect to net calcification rate (*R*^2^ = 0.461, *p* = 0.01). Measured Ba/Ca significantly varies a parabolic pattern with increasing net calcification rate in the coralline red alga, lobster, and oyster (*R*^2^ = 0.733, *p* = 0.02; *R*^2^ = 0.99, *p* = 0.05; *R*^2^ = 0.661, *p* = 0.03), while increasing linearly for the serpulid worm tube and hard clam (*R*^2^ = 0.571, *p* = 0.003; *R*^2^ = 0.211, *p* = 0.02).

The [CO_3_^2−^] of the culture seawater exhibited 28 significant relationships with X/Ca ratios across all of the studied species (Table 4). Organisms that exhibited the most significant relationships with [CO_3_^2−^] are the coral, limpet, pencil urchin, and serpulid worm. The elements that most frequently varied with [CO_3_^2−^] were Sr/Ca, Zn/Ca, and Ba/Ca. Sr/Ca significantly decreases with decreasing [CO_3_^2−^] in the serpulid worm and a number of molluscs, including the oyster, soft clam, conch, whelk, and limpet. The mixed mineralogy serpulid worm and the oyster both decrease parabolically with decreasing [CO_3_^2−^] (*R*^2^ = 0.309, *p* = 0.008; *R*^2^ = 0.541, *p* = 0.004), while the aragonitic soft clam and conch decrease following inverse parabolic patterns (*R*^2^ = 0.66, *p* = 0.001; *R*^2^ = 0.354, *p* = 0.004). The aragonitic gastropod molluscs, the whelk and limpet, both linearly decrease with decreasing [CO_3_^2−^] (*R*^2^ = 0.156, *p* = 0.03; *R*^2^ = 0.201, *p* = 0.03). Zn/Ca also systematically varies with decreasing seawater [CO_3_^2−^] in the coralline red algae, serpulid worm, coral, blue mussel, and gulf shrimp. Yet, despite sharing the same high-Mg calcite mineralogy, Zn/Ca levels in shrimp are an order of magnitude higher than those measured in the coralline red algae. For the coral, Zn/Ca linearly increases with decreasing seawater [CO_3_^2−^] (*R*^2^ = 0.0982, *p* = 0.0005), while the Zn/Ca ratio in the serpulid worms and blue mussels and span comparable ranges and exhibit similar inverse parabolic patterns with decreasing [CO_3_^2−^] (*R*^2^ = 0.258, *p* = 0.02; *R*^2^ = 0.539, *p* = 0.04). Significant changes in biomineral Ba/Ca occur with decreasing seawater [CO_3_^2−^] were observed for the lobsters, echinoderms, and a suite of molluscs, including the hard clam, oyster, conch, and whelk. In the calcitic oyster, the decrease is slight and linear (*R*^2^ = 0.632; *p* = 0.004), while in the aragonitic hard clam, conch, and whelk, Ba/Ca exhibits an inverse parabolic pattern with decreasing [CO_3_^2−^] (*R*^2^ = 0.784, *p* = 0.0004; *R*^2^ = 0.257, *p* = 0.004; *R*^2^ = 0.193, *p* = 0.04). A similar inverse parabolic pattern in Ba/Ca with decreasing seawater [CO_3_^2−^] emerges for the high-Mg calcite lobster and pencil urchin (*R*^2^ = 0.763, *p* = 0.006; *R*^2^ = 0.752, *p* < 0.0001), and the purple urchin exhibits a linear increase in Ba/Ca with decreasing seawater [CO_3_^2−^] (*R*^2^ = 0.335, *p* = 0.01).

The pH_*sw*_ of the culture tanks was measured, and the pH_*cf*_ has been calculated for 10 of the species based on δ^11^B measurements of the same specimens evaluated in the present study (Liu et al., 2020). Twenty-five significant linear or quadratic relationships were identified between pH_*sw*_ and elemental ratios across all species. Of the 10 species with pH_*cf*_ data, there were 13 significant relationships between pH_*cf*_ and elemental ratios. The species that exhibit the most significant relationships between elemental ratios and pH_*sw*_ are the coral (3; Supplementary Figures 22, 23), coralline red alga (3; Supplementary Figures 24, 25), limpet (3; Supplementary Figures 32, 33) and pencil urchin (3; Supplementary Figures 34, 35). The coralline red alga exhibits the most significant relationships between elemental ratios and pH_*cf*_ (4; Supplementary Figures 24, 25). The elements that most frequently vary with pH_*sw*_ are B/Ca, Sr/Ca, and Ba/Ca. Likewise, B/Ca and Sr/Ca most often correlate with pH_*cf*_. B/Ca significantly decreases with decreasing pH_*sw*_ in the coralline red alga, pencil urchin, and limpet, while significantly increasing with pH_*cf*_ in the coralline red algae, serpulid worm, and hard clam. For both pH_*sw*_ and pH_*cf*_, the high-Mg calcite coralline red alga appears to maintain its B/Ca under moderately lower pH_*sw*_ conditions before exhibiting a decrease under even more acidic conditions (*R*^2^ = 0.695, *p* = 0.002; *R*^2^ = 0.681, *p* = 0.004). Measured Sr/Ca decreases significantly with decreasing pH_*sw*_ in the serpulid worm and a suite of molluscs, including the oyster, soft clam, conch, whelk, and limpet. Sr/Ca significantly increases in the hard clam and pencil urchin, and significantly decreases in the oyster with increasing pH_*cf*_. The Sr/Ca of the serpulid worm and the calcitic oyster exhibit parabolic trends with decreasing pH_*sw*_604781529604781529 (*R*^2^ = 0.285, *p* = 0.01; *R*^2^ = 0.356, *p* = 0.03). The Sr/Ca of the soft clam, whelk and limpet decrease with decreasing pH_*sw*_; however, they differ in that Sr/Ca of the soft clam follows an inverse parabolic pattern, while Sr/Ca of the whelk and limpet decrease linearly (*R*^2^ = 0.723, *p* = 0.0003; *R*^2^ = 0.171, *p* = 0.0254). For pH_*cf*_, similar to pH_*sw*_, the molluscan Sr/Ca varies in different ways: Sr/Ca of the aragonitic hard clam decreases parabolically with pH_*cf*_ (*R*^2^ = 0.639; *p* = 0.001), while Sr/Ca of the low-Mg calcite oyster decreases linearly with pH_*cf*_ (*R*^2^ = 0.602; *p* = 0.001).

## DISCUSSION

The data presented survey nine elemental ratios in 18 marine calcifying species and thus represent a unique opportunity to examine carbonate elemental geochemistry from the perspective of comparative organismal biology. One of the main strengths of this analysis is its revelation of large-scale patterning of element incorporation amongst different CaCO_3_ mineralogies, species, and seawater carbonate chemistries. It also allows examination of the sensitivity of elemental partitioning in biogenic carbonates to changes in external (pH_*sw*_) and internal pH (pH_*cf*_; boron-isotope-based; Liu et al., 2020) conditions. For many of the investigated species, elemental data were only available for 2–3 individuals per *p*CO_2_ treatment. Although some relationships may not be apparent due to small sample size, interesting and statistically significant relationships were still evident (Tables 3–5; red outlines, Supplementary Figures 10–45). Nevertheless, these relationships warrant further investigation due to the small sample sizes involved.

Precipitation rates are known to affect elemental partition coefficients in inorganically precipitated calcium carbonates to varying degrees (See Supplementary Table 5 and references therein). Ries et al. (2009) illustrated that the cultured species exhibit diverse responses in net calcification rate to increasing *p*CO_2_ (Table 1). Though net calcification rates of whole organisms cannot easily be related to the growth rates of calcium carbonate crystals, there are a number of significant relationships between X/Ca and the determined net calcification rate of the species across the *p*CO_2_ conditions for 10 of the 18 species (Table 3). Mg/Ca, Sr/Ca, and Ba/Ca were the most common elemental ratios that varied significantly with net calcification rates across the 10 species, while Li/Ca, Zn/Ca, Cd/Ca, and U/Ca were the least common. B/Ca only significantly varied with net calcification rate in the pencil urchin, *E. tribuloides*. However, despite there being a number of significant relationships between X/Ca and net calcification rate in the cultured species, the signs and shapes of many of the relationships differ from those published for inorganic minerals (e.g., Gabitov et al., 2014; Holcomb et al., 2016; Mavromatis et al., 2018).

Boron was among the most responsive elements to seawater carbonate chemistry, showing statistically significant trends in five of the 18 species. For example, the B/Ca in the pencil urchin was highly sensitive (over 200 μmol/mol, 62% of the mean, across treatments; Supplementary Figures 34, 35) to manipulation of seawater carbonate chemistry, while B/Ca in the purple urchin was not (Supplementary Figures 38, 39). B/Ca in the coralline red algae was also highly sensitive to seawater [CO_3_^2−^] (Supplementary Figures 24, 25). U/Ca was also found to be sensitive to seawater [CO_3_^2−^] in the temperate coral (Supplementary Figure 23), the limpet (Supplementary Figure 32) and the pencil urchin (Supplementary Figure 34). Significant trends were also observed for many other elements, including Mg, Ba, Sr, Zn, and U. In some cases, significant relationships between element/Ca ratios and pH_*cf*_ derived from boron isotopes (Liu et al., 2020) were apparent when relationships with external pH_*sw*_ were not (e.g., Hard clam; Table 5 and Supplementary Figures 30, 31). These data suggest that seawater carbonate chemistry influences X/Ca ratios in a range of calcifying species, provide launching points for exploring the viability of these elements as palaeoceanographic proxies of seawater carbonate chemistry.

Regarding large-scale patterns in the study, most elements showed significant variability amongst species, and apparent biomineral partition coefficients were often quite different from empirically derived elemental partition coefficients for calcite, aragonite, or ACC precipitated inorganically from seawater (Table 2 and Figure 3). Deviations from inorganic mineral relationships reveal the influence that different biomineralization mechanisms have on the pathways that ions take to the site of calcification. Some clear patterns emerged in this regard. For example, Mg/Ca in mollusc shells was usually low regardless of shell polymorph mineralogy, and Sr/Ca was low in all the biominerals compared to ratios in inorganic precipitates, with coral and *Halimeda* aragonite exhibiting higher Sr/Ca compared to other species, and aragonite molluscs exhibiting especially low Sr/Ca. Strikingly, mollusc CaCO_3_ (of all polymorphs) was relatively low in all X/Ca ratios compared to other species, except for Li/Ca (compared to the temperate coral) and Na/Ca.

The partition coefficients for some elements in mollusc shells, including for Li and B, were notably similar to recently published elemental data for inorganically precipitated ACC (e.g., *K*_*Li*_ in ACC: 6.5E-3–14.5E-3; *K*_*Li*_ in the oyster: 9.5E-3; Table 2; Evans et al., 2020), regardless of mollusc polymorph mineralogy (Figure 3). This is noteworthy as molluscs are known to form their shells from ACC precursors (Addadi et al., 2003), whereas the evidence and importance of ACC in some of the other species examined here are unclear. However, it should be noted that ACC precursor phases may dissolve and reprecipitate in their transformation to crystalline calcium carbonate, potentially involving the exchange of elements with the surrounding fluid (e.g., Giuffre et al., 2015). Since most of the apparent partition coefficients do not resemble those reported for ACC, it is not known how pertinent the elemental data for ACC (e.g., Evans et al., 2020) are to crystalline biominerals. Nevertheless, the data in the present study provide an interesting point of comparison.

Varying levels of biological control on biomineralization are apparent across the phyla studied when assessing elements individually (Figures 1, 3; Dove, 2010). However, clear groupings of species arise when evaluating the elemental ratios together (Figures 4–6). A possible explanation is that species most enriched in Mg tend to also exhibit high ratios of other elements (Figure 2). This is most apparent for the organisms that produce high-Mg calcite: the arthropods, echinoderms, coralline red algae, and serpulid worms. This scenario has been demonstrated in synthetic calcites, where higher incorporation of Mg^2+^ led to higher incorporation of Sr^2+^ due to distortion of the crystal lattice (Mucci and Morse, 1983). Although this scenario may explain the behavior of some of the measured elements (e.g., Li/Ca; Figure 2), many cannot be explained in this manner. To explore potential explanations for the variability in these patterns, the GAMs demonstrate that accounting for phylum and/or mineralogy substantially improves the models’ power in predicting X/Ca in the studied species (Table 6). Furthermore, when analyzed in an evolutionary context (i.e., time-calibrated phylogenetic tree), X/Ca ratios exhibit strong phylogenetic signals, with the differences in elemental ratios between species largely reflecting phylogenetic distance (Table 2). Clearly, Mg/Ca alone cannot explain the abundance of other elements via lattice distortion, and our GAMs provide evidence that mineralogy and phylum are key predictors of elemental ratios in the studies species. Thus, explanations for differences in elemental ratios rooted in differences in the species’ mechanisms of biomineralization must be explored.

Calcitic bivalve molluscs are known to strongly and actively exclude Mg from their biominerals to form low-Mg calcite, as shown specifically for the blue mussel (*M. edulis*; Lorens and Bender, 1977). The exclusion of trace and minor elements from molluscan biominerals is likely associated with regulation of the ionic composition of the parent fluid for calcification, as well as potentially other processes, such as detoxification (*L. littorea*; Mason and Nott, 1981). The data presented here suggest that this or similar processes are also active in these bivalves and gastropods, and that other elements are also excluded during the mollusc biomineralization process, given the low elemental ratios and low apparent partition coefficients compared to those of inorganically precipitated calcite (Figures 1, 3).

Amongst the high-Mg calcitic species investigated, comparisons between the arthropods and echinoderms are particularly revealing. Both exhibit similar Mg/Ca, but the crustaceans are relatively enriched in the other elemental ratios. Echinoderms and arthropods form their calcareous exoskeletons by transporting amorphous calcium carbonate to be co-precipitated with an organic matrix that appears to serve as a mineralogical template, but has different B, Ba, and Sr compositions (Roer and Dillaman, 1984; Märkel et al., 1986; Beniash et al., 1997; Dillaman et al., 2005). The high-Mg content can be explained by way of an amorphous precursor, as ACC has been shown to incorporate up to 20 mol% Mg during its transformation into Mg-calcite (Purgstaller et al., 2016); however, the source of discrepancy between the other elements is less clear (see reviews by Long et al. (2014) and Evans et al. (2020)). Some crustacea, including the American lobster evaluated here, maintain a stabilized ACC phase as a predominant component of their fully calcified cuticle in addition to the layer of high-Mg calcite in the exocuticle (Levi-Kalisman et al., 2002; Mergelsberg et al., 2019; Evans et al., 2020). Arthropods are also unique in their ability to bi-directionally transport Ca^2+^ and CO_3_^2−^ when resorbing the old cuticle during pre-molting, which could lead to element accumulation and possibly explain the enrichment in X/Ca ratios relative to the echinoderms.

Echinoderms and arthropods also differ in the extent to which they isolate their site of calcification from seawater and utilize different types of cells to transport ACC to the site of calcification—primary mesenchyme cells (PMCs) in echinoderms and epithelial cells in the arthropods (Roer and Dillaman, 1984; Märkel et al., 1986). Echinoderm PMCs transport ACC to the site of calcification and form a syncytium of other cells that maintain favorable calcification conditions. The pH within PMCs is maintained at approximately 6.9; however, the pH_*cf*_ within the syncytium is typically equivalent to that of seawater (Stumpp et al., 2012). Despite this, [HCO_3_^−^] in the syncytium is maintained at higher levels relative to seawater (Holtmann, 2013). Therefore, these fundamentally different modes of biomineralization in arthropods and echinoderms may both support the formation of high-Mg calcite, but with differing compositions of other elements.

The species that exhibit the highest Mg/Ca ratios, the serpulid worm and the coralline red alga, also exhibit the highest levels of the other X/Ca ratios. Here, too, there are no significant relationships of any X/Ca ratio with Mg/Ca (Figure 2). Given that these two species are phylogenetically distant, one an animal and the other an alga, it is interesting that they cluster together in the multivariate analyses (Figures 4, 5). Figure 3 demonstrates that the apparent partition coefficients calculated for the serpulid worm typically either agree with the published values for inorganic calcite or aragonite or lie in the middle of the two (Table 2). This may indicate that, although generally enriched in X/Ca ratios, the differences between the serpulid worm and the coralline red algae potentially arise from the bimineralic composition of the serpulid worm tube sampled here. Serpulid worms and coralline red algae, including the species investigated here, have been shown to elevate pH at the site of calcification (Chan et al., 2015a; Short et al., 2015; Cornwall et al., 2017; Donald et al., 2017; Sutton et al., 2018). Although these processes may support calcification, they do not explain the high X/Ca ratios measured in the coralline red algae. Coralline red algae biomineralize intercellularly, outside of the cell membrane but within and between cell walls (Ries, 2006; Long et al., 2014). Evidence of ACC has been observed at the growing edge of a coralline red algal biomineral (Raz et al., 2000; Rahman et al., 2019). Stabilized ACC does not dominate the biomineral phase produced by coralline red algae, as it does for the lobster, but the potential formation of coralline red algal high-Mg calcite via ACC could explain the very high Mg component (Purgstaller et al., 2016; Mergelsberg et al., 2019; Evans et al., 2020). It has also been shown that various biomolecules that compose the organic matrices and templates, in conjunction with Mg^2+^ ions in solution, can lead to higher levels of Mg^2+^-incorporation in calcite (Long et al., 2014).

The green alga *H*. *incrassata* and the temperature coral *O. arbuscula* group together in the hierarchical clustering and multivariate analyses of the measured elements, but this may simply be driven by their shared aragonitic mineralogy. Evidence for pH elevation at the site of calcification has also been shown for this coral species and this and other species of *Halimeda* green algae (Liu et al., 2020; see review by Borowitzka and Larkum, 1987). Both of the species photosynthesize and precipitate aragonite biominerals (Ries et al., 2009; Ries, 2011), but that is generally where their similarities in biomineralization end. For the *Halimeda* alga, precipitation occurs extracellularly within invaginated sections of a syncytial cell wall (Borowitzka and Larkum, 1987; Ries, 2009; Cuif et al., 2013). Conversely, the *Oculina* coral apparently mineralizes in fluid pockets beneath the calicoblastic epithelium (Tambutté et al., 2011). Though there is currently debate surrounding the precise mechanisms of ion transport and the potential role of organic templates for mineral nucleation in scleractinian corals, there is general agreement that the conditions at the site of calcification and the mineralization process are highly controlled (Ogilvie, 1896; Cohen and McConnaughey, 2003; Mass et al., 2017; Von Euw et al., 2017).

Qualitatively, it does not appear that elemental chemistry perfectly recapitulates taxonomy due to the confounding influence of calcium carbonate polymorph (Figures 4B–D). However, the dendrogram produced by the hierarchical clustering analysis of the elemental chemistry of the studied species (Figure 4A) visually recapitulates phylogenetic relationships amongst the aragonitic (Figure 4B) and calcitic species (Figure 4C). However, mixed mineralogy whelk and serpulid worm do not exhibit this taxonomic clustering. Even within the molluscs, the bivalve and gastropod molluscs groups are distinct in Figure 4A, potentially revealing clustering at the class level. A similar class-level clustering for P/Ca and Mg/Ca has also been identified in the Malacostraca (Mergelsberg et al., 2019). The whelk is further removed from the other aragonitic gastropods in the hierarchical cluster analysis, most likely due to its bimineralic nature (~75% aragonite: 25% calcite; Ries, 2011). The dislocation of the serpulid worm tube in the hierarchical clustering analysis, which is relatively closely related to the molluscs, probably arised from its bimineralic composition (*ca*. 50% aragonite: 50% calcite; Ries, 2011). The aragonitic coral and green algae are the least related to everything else in the aragonitic tree and are also closest in the clustering analysis of the elemental data. Collectively, these results suggest that the divergence in X/Ca ratios between species is related to both phylogeny and mineralogy.

The wide array of biomineralization pathways amongst the studied species makes it difficult to deconvolve the effects of biological processes from physicochemical processes. Although the strong phylogenetic signal detected across all X/Ca ratios using phylogenetic analyses is partly driven by patterns of elemental congruence within aragonitic and calcitic groups (Figures 4A–D), using both phylogenetic and non-phylogenetic regression models to examine interspecific differences in X/Ca ratios show that phylogeny explains more of the variance in the X/Ca ratios than mineralogy (Figure 7 and Supplementary Table 9).604781538604781538

NMDS ordination visualizes this primary clustering of organisms by phyla as well as the secondary clustering by mineralogy (Figures 5, 6). Clustering by mineralogy is not unexpected, as differences in X/Ca ratios between calcite and aragonite are well documented via inorganic precipitation experiments (e.g., Gabitov et al., 2011, 2019; Mavromatis et al., 2015, 2018). Furthermore, the phylogenetic analyses only including the Molluscan data show that clustering of elemental ratios by phylogenetic proximity is relevant even at the class level (see section “Phylogenetic Signal and Regression Analyses”). Future studies should examine whether the apparent accord between phylogeny and elemental clustering at the class level within Mollusca holds true when including more species, including ones with mixed mineralogies. Regardless, an interesting and new result of this work is the strong phylogenetic signal within the elemental clustering of the studied species, even after controlling for the effects of mineralogy (Figures 3–7).

The present study has implications for interpreting carbonate preservation and diagenesis in the sedimentary record. By systematically defining the plausible ranges and patterns of elemental partitioning in a diverse range of modern calcifiers, for example by multivariate ordination analysis (e.g., Figures 5, 6), measurements of paleo-carbonates can be placed within this framework to assess whether they fall within the range of compositions of evolutionarily-related and mineralogically similar organisms. If they do not, it may indicate diagenetic alteration of the elemental composition of the fossil—rendering it unsuitable for paleo-reconstructions.

In sum, it is challenging to unequivocally attribute specific aspects of species’ biomineralization pathways to differing patterns of elemental partitioning in biogenic carbonates. The present study shows that the broad patterning of elemental partitioning within marine calcifiers reflects not only environmental conditions, but also phylogeny, potentially via the molecular and structural mechanisms that they evolved to support the formation of their protective shells and skeletons. With more work, mechanistic explanations of elemental partitioning in biogenic carbonates should be achievable.

## Supplementary Material

Data and supporting figures

Supporting tables

## Figures and Tables

**TABLE 1 |:** Species examined in this study with supporting data.

Organism	Latin	Phylum	Mineralogy^#,21^	Net calcification rate response^22^	pH_*cf*_ (±SD)^23^	ΔpH^23^	ΔpH Response^23^	Amorphous precursor?	Biomineralization mode
American Lobster	*Homarus americanus*	Arthropoda	HMC	Threshold positive	–	–	–	Yes^25,26,27^	Extracellular
Blue Crab	*Callinectes sapidus*	Arthropoda	HMC	Positive	7.965 (0.06)	−0.065	Neutral	Yes^24,26,27^	Extracellular
Gulf Shrimp	*Penaeus plebejus*	Arthropoda	HMC	Positive	8.6 (0.12)	0.57	Positive	Yes^26,27^	Extracellular
Blue Mussel	*Mytilus edulis*	Mollusca	97LMC:3A	Neutral	7.62 (0.36)	−0.53	Non-linear positive	Yes	Extracellular
Oyster	*Crassostrea virginica*	Mollusca	LMC	Negative	7.76 (0.38)	−0.39	Non-linear positive	Yes^1.2.3^	Extracellular
Hard Clam	*Mercenaria mercenaria*	Mollusca	97A: 3HMC	Threshold negative	7.89 (0.30)	−0.22	Positive	Yes^1,2,3^	Extracellular
Soft Clam	*Mya arenaria*	Mollusca	97A:3HMC	Negative	–	–	–	Yes^4,5.6^	Extracellular
Bay Scallop	*Argopecten irradians*	Mollusca	97LMC:3A	Negative	–	–	–	Yes^7^	Extracellular
Conch	*Strombus alatus*	Mollusca	97A:3LMC	Threshold negative	–	–	–	Yes^2,8^	Extracellular
Periwinkle	*Littorina littorea*	Mollusca	97LMC:3A	Negative	–	–	–	Yes^2,8^	Extracellular
Whelk	*Urosalpinx cinerea*	Mollusca	75A:25LMC	Negative	–	–	–	Yes^2,8^	Extracellular
Limpet	*Crepidula fornicata*	Mollusca	97A:3LMC	Parabolic	–	–	–	Yes^9^	Extracellular
Purple Urchin	*Arbacia punctulata*	Echinodermata	HMC	Parabolic	7.81 (0.15)	−0.23	Positive	Yes	Intercellular^15^
Pencil Urchin	*Eucidaris tribuloides*	Echinodermata	HMC	Threshold negative	8.13 (0.02)	0.09	Non-linear positive	Yes	Intercellular^15^
Red Coralline Algae	*Neogoniolithon spectabile*	Rhodophyta	HMC	Parabolic	9.22 (0.16)	1.03	Neutral	Yes^10,11^	Intercellular^16,17^
Green Halimeda Algae	*Halimeda incrassata*	Chlorophyta	A	Parabolic	–	–	–	–	Intercellular^16,18^
Serpulid worm	*Hydroides crucigera*	Annelida	~50A:50HMC	Negative	8.17 (0.02)	0.06	Non-linear positive	Yes^12,13,14^	Intracellular^13,14^
Temperate Coral	*Oculina arbuscula*	Cnidaria	A	Threshold negative	8.52 (0.01)	0.41	Positive	Yes (debated)	Extracellular^19,20^

# High Mg-calcite (HMC), low Mg-calcite (LMC), aragonite (A); mineralogy is considered mixed if the proportions exceed a 90:10 split; otherwise, if less, the biomineral is considered to be called the dominant mineral [e.g., 97A:3LMC is considered to be A in the following analyses as the detection limit for the XRD used in Ries (2011) as 3%]. More specific quantification regarding relative proportions of each mineralogy can be found in Ries, 2011;

1 Weiss et al., 2002;

2 Addadi et al., 2006;

3 Immenhauser et al., 2016;

4 Harper et al., 1997;

5 Gazeau et al., 2013;

6 Meseck et al., 2018;

7 Brecevic and Nielsen, 1989;

8 Nassif et al., 2005;

9 Heinemann et al., 2011;

10 Leptophytum foecundum; Rahman et al., 2019;

11 Corallina elongate; Raz et al., 2000;

12 De Yoreo et al., 2015;

13 Chan et al., 2015a;

14 Chan et al., 2015b;

15 Wilt, 2002;

16 Halimeda sp.; Cuif et al., 2013;

17 Borowitzka and Larkum, 1987;

18 Halimeda; Wilbur et al., 1969;

19 Constantz, 1986;

20 Constantz and Meike, 1989;

21 Ries, 2011;

22 Ries et al., 2009;

23 Liu et al., 2020;

24 Dillaman et al., 2005;

25 Mergelsberg et al., 2019;

26 Luquet, 2012;

27 Roer and Dillaman, 1984.

**TABLE 2 |:** Summary of partition coefficients, including calculated apparent partition coefficients of the species cultured in this study; imputed values; synthetic calcite, aragonite, and amorphous calcium carbonate (ACC); and a number of published biogenic apparent partition coefficients.

Organism		K_*Li*_ (SD)	K_*B*_ (SD)	K_*Na*_ (SD)	K_*Mg*_ (SD)	K_*Zn*_ (SD)	K_*Sr*_ (SD)	K_*Cd*_ (SD)	K_*Ba*_ (SD)	K_*U*_ (SD)
Temperate Coral	This study	0.0028 (0.0004)	0.014 (0.0009)	0.506*	0.0008 (0.00007)	7.853 (4.144)	0.0011 (0.00004)	0.647 (1.044)	1.439 (0.195)	0.676 (0.0948)
Halimeda Algae	This study	0.0081*	0.0024*	0.259 (0.029)	0.0009 (0.0007)	0.252 (0.192)	0.001 (0.00008)	5.736*	2.525 (0.697)	0.0255*
Soft Clam	This study	0.00215 (0.00023)	0.00064 (0.00011)	0.490*	0.00015 (0.00003)	3.966 (2.022)	0.00029 (0.00003)	0.906 (1.220)	0.7146 (0.4464)	0.0311 (0.0153)
Conch	This study	0.002 (0.0003)	0.0003 (0.00005)	0.567 (0.038)	0.0002 (0.00006)	2.84 (1.67)	0.0002 (0.00003)	0.187 (0.113)	0.418 (0.242)	0.014 (0.007)
Hard Clam (Quahog)	This study	0.00185 (0.00131)	0.00066 (0.00050)	0.482*	0.0031 (0.0044)	185.46 (184.71)	0.00026 (0.00008)	0.10166 (0.92659)	0.81877 (0.48562)	0.12254 (0.13280)
Limpet	This study	0.0019 (0.00013)	0.0004 (0.00009)	0.492 (0.050)	0.0002 (0.00008)	4.28 (4.39)	0.00018 (0.000021)	0.182 (0.133)	0.652 (0.793)	0.045 (0.015)
Whelk	This study	0.002 (0.0003)	0.0004 (0.00007)	0.508 (0.033)	0.0002 (0.00009)	12.31 (20.43)	0.0002 (0.00003)	17.84 (48.82)	0.492 (0.555)	0.049 (0.022)
Serpulid Worm	This study	0.0195 (0.0039)	0.0117 (0.0014)	0.433*	0.0316 (0.0044)	11.75 (6.06)	0.0007 (0.00009)	9.775 (8.302)	8.177 (6.382)	0.388 (0.098)
Blue Mussel	This study	0.0028 (0.0005)	0.00102 (0.00025)	0.481*	0.0052 (0.0058)	13.323 (5.825)	0.00025 (0.00017)	0.5478 (0.3158)	3.1824 (6.9398)	0.0048 (0.0010)
Periwinkle	This study	0.006 (0.002)	0.00007 (0.00002)	0.366 (0.065)	0.0008 (0.0003)	12.63 (10.09)	0.0001 (0.00001)	0.064 (0.061)	0.144 (0.051)	0.007 (0.004)
American Oyster	This study	0.0095 (0.0017)	0.0025 (0.00025)	0.459*	0.0021 (0.0003)	52.67 (29.13)	0.00012 (0.000013)	0.365 (0.121)	0.0958 (0.0144)	0.00697 (0.0194)
Bay Scallop	This study	0.0047 (0.001)	0.0008 (0.0002)	0.4787*	0.0038 (0.0008)	18.098 (11.507)	0.00016 (0.000008)	0.0396 (0.0124)	0.5489 (0.1901)	0.00287 (0.00156)
American Lobster	This study	0.0122*	0.0058*	0.688 (0.353)	0.015 (0.004)	1378.00 (698.64)	0.0006 (0.0001)	6.65*	11.69 (4.64)	0.0184*
Blue Crab	This study	0.0132*	0.0052*	0.438 (0.083)	0.011 (0.001)	398.35 (162.77)	0.0006 (0.00004)	6.88*	4.17 (2.62)	0.4380*
Gulf Shrimp	This study	0.0125*	0.0049*	0.381 (0.101	0.009 (0.004)	2009.32 (1101.27)	0.0006 (0.0002)	6.918*	6.66 (4.69)	0.0258*
Coralline Red Alga	This study	0.018 (0.001)	0.012 (0.002)	0.337 (0.027)	0.052 (0.002)	142.16 (64.98)	0.0004 (0.00002)	22.87 (8.86)	2.31 (0.52)	0.108 (0.012)
Pencil Urchin	This study	0.02 (0.0005)	0.008 (0.001)	0.450 (0.031)	0.013 (0.0014)	9.945 (5.854)	0.0002 (0.000008)	7.118 (1.893)	0.9967 (0.2938)	0.0102 (0.0043)
Purple Urchin	This study	0.023 (0.002)	0.010 (0.0004)	0.517 (0.030)	0.015 (0.001)	3.99 (1.99)	0.0003 (0.00001)	4.0000 (0.4585	0.6995 (0.2267)	0.005 (0.0008)
Published inorganic aragonite	See Supplementary Table 5	0.0321	0.02–2.48	–	4.72E-04	5.7	1.13	–	0.22–2.11	0.248
Published inorganic calcite	See Supplementary Table 5	1.4E-4–4.9E-3	1.4E-6–4.14E-3	–	2.66 × 10^−2^	54	2.59 × 10^−1^	18.5	4.0E-3–96.3	2.0–6.0 × 10^−2^
Published ACC	See Supplementary Table 5	6.5–14.5E-3	0.01–2.34	0.6–4.06E-3	0.014–0.221	–	0.18–1.02	–	0.35–5.97	0.01–0.94
Coral	Published	1.22E-3–2.12E-3^1^	2.63E-05^2^	–	3.41E-4^1,2^–2.37^3^		1.035^2^–2051^3^		3770^3^	0.0647^2^
Mollusc	Published	0.003–0.01^4^	–	–	0.0002–0.002^4^	–	0.116–0.23^4,6^	–	0.1–1.29^5,6^	0.4125^7^
Echinoderms	Published	0.0111–0.0958^9^	0.0203–0.0376^9^	–	0.0218–0.0338^8,9^	–	0.222–0.269^8^	–	–	–
Arthropods	Published				0.00358–0.022^10,11^		36.65–58.61^10^			
Algae	Published		0.013^13^	–	0.03–0.055^12^	–	0.34^12^	–	–	–
Brachiopods	Published	0.0091–0.0188^14^	0.0297–1.2351^14^	0.0001–0.0003^14^	0.0008–0.0019^14^		0.1332–0.2807^14^		0–0.4239^14^	
Foraminifera	Published	5.85E-03^19^	1.19E-03^15,19^	1.35E-04^19^	0.00031–0.00278^18,19^	–	0.159–0.33^18,19^	0.8–4^15,17,19^	0.20–0.7^17,19^	31^16,19^

1 Montagna et al., 2014;

2 Sinclair and Risk, 2006 (model);

3 Gaetani et al., 2011 (model);

4 Calculated from Dellinger et al., 2018;

5 Gillikin et al., 2006;

6 Zacherl et al., 2003;

7 Frieder et al., 2014;

8 Lavigne et al., 2013;

9 Nguyen, 2013;

10 Wickins, 1984;

11 Kondo et al., 2005;

12 Ries, 2006;

13 Donald et al., 2017;

14 Jurikova et al., 2020;

15 Dang et al., 2019;

16 Keul et al., 2013;

17 Havach et al., 2001;

18 Raitzsch et al., 2010;

19 Allen et al., 2016.

* Refers to the apparent partition coefficients that were calculated using imputed values.

**TABLE 3 |:** Summary of AIC test results for significant relationships (*p* < 0.05) between element/Ca ratios and net calcification rates across all organisms.

Organism	Phylum	X/Ca	Best-fit regression			
			TYPE	*p*	*R* ^2^	Relationship
American lobster	Arthropoda	Ba	Para	0.00498	0.99	+
Blue mussel	Mollusca	Sr	Para	0.0146	0.452	+
		Cd	Para	0.0192	0.999	−
American oyster	Mollusca	Li	Para	0.00317	0.916	+
		Ba	Para	0.0288	0.661	+
Hard clam	Mollusca	Mg	Linear	0.0276	0.251	+
		Sr	Linear	0.0413	0.213	+
		Ba	Linear	0.0026	0.571	+
		U	Linear	0.0284	0.508	+
Soft clam	Mollusca	Zn	Linear	0.0446	0.595	−
		Sr	Linear	0.000196	0.742	+
		Cd	Linear	0.0149	0.86	−
Bay scallop	Mollusca	Mg	Para	0.00587	0.61	−
Limpet	Mollusca	Li	Para	0.0127	0.831	+
		Mg	Para	0.00632	0.471	+
		Sr	Linear	0.0196	0.325	+
Pencil urchin	Echinodermata	B	Linear	0.00694	0.623	+
		Sr	Para	0.00707	0.461	−
		U	Para	0.0151	0.816	+
Coralline red alga	Rhodophyta	Ba	Para	0.0159	0.733	+
Serpulid worm	Annelida	Ba	Linear	0.0182	0.211	+
Temperate coral	Cnidaria	Li	Linear	0.00222	0.165	−
		Mg	Para	0.0318	0.0462	+
		Zn	Linear	0.00321	0.0798	−

**TABLE 4 |:** Summary of AIC test results for significant relationships (*p* < 0.05) between element/Ca ratios and seawater carbonate ion concentrations across all organisms.

Organism	Phylum	X/Ca	Best-fit regression			
			TYPE	*p*	*R* ^2^	Relationship
American lobster	Arthropoda	Ba	Para	0.00562	0.763	−
Gulf shrimp	Arthropoda	Zn	Para	0.0302	0.479	+
Blue mussel	Mollusca	Zn	Para	0.0414	0.539	−
American oyster	Mollusca	Sr	Para	0.00373	0.541	+
		Ba	Linear	0.00365	0.632	+
Hard clam	Mollusca	Ba	Para	0.000406	0.784	−
Soft clam	Mollusca	Sr	Para	0.00106	0.66	−
Conch	Mollusca	Sr	Para	0.00391	0.354	−
		Ba	Para	0.0171	0.257	−
Whelk	Mollusca	Mg	Linear	0.00817	0.245	+
		Sr	Linear	0.0319	0.156	+
		Ba	Para	0.0403	0.193	−
Limpet	Mollusca	B	Linear	0.00406	0.675	+
		Sr	Linear	0.0271	0.201	+
		U	Linear	0.00535	0.597	+
Pencil urchin	Echinodermata	B	Linear	<0.0001	0.88	+
		Ba	Para	<0.00001	0.752	−
		U	Para	<0.0001	0.982	+
Purple urchin	Echinodermata	Ba	Linear	0.014	0.335	−
Coralline red alga	Rhodophyta	B	Para	0.00357	0.651	−
		Zn	Para	<0.00001	0.648	+
Serpulid worm	Annelida	Li	Linear	0.0422	0.287	−
		Mg	Para	0.0107	0.554	−
		Zn	Para	0.0227	0.258	−
		Sr	Para	0.00799	0.309	+
Temperate coral	Cnidaria	Li	Linear	0.00208	0.147	−
		Zn	Linear	0.000476	0.0982	−
		U	Linear	0.0435	0.0524	+

**TABLE 5 |:** Summary of AIC test results for significant relationships (*p* < 0.05) between element/Ca ratios and seawater pH and δ^11^B-derived calcifying fluid pH (indicated with bold italic text) across all organisms.

Organism	Phylum	X/Ca	pH_*sw*_ or pH_*cf*_	Best-fit regression			
				TYPE	*p*	*R* ^2^	Relationship
American lobster	Arthropoda	Ba	pH_*sw*_	Para	0.00562	0.763	−
American oyster	Mollusca	Sr	pH_*sw*_	Para	0.0284	0.356	+
			***pH***_***cf***_	***Linear***	***0.000675***	***0.602***	**−**
		Ba	pH_*sw*_	Linear	0.00136	0.71	+
			*pH*_*cf*_	*Para*	*0.0221*	*0.695*	+
Hard clam	Mollusca	Ba	pH_*sw*_	Linear	0.0308	0.326	+
		***B***	***pH***_***cf***_	***Para***	***0.000625***	***0.963***	**+**
		***Sr***	***pH***_***cf***_	***Para***	***0.00146***	***0.639***	**+**
Soft clam	Mollusca	Sr	pH_*sw*_	Para	0.000343	0.723	−
		Cd	pH_*sw*_	Linear	0.0491	0.577	−
Conch	Mollusca	Sr	pH_*sw*_	Para	0.0156	0.263	−
Periwinkle	Mollusca	Ba	pH_*sw*_	Linear	0.0482	0.155	−
Whelk	Mollusca	Mg	pH_*sw*_	Linear	0.00441	0.283	+
		Sr	pH_*sw*_	Linear	0.0254	0.171	+
Limpet	Mollusca	B	pH_*sw*_	Linear	0.00404	0.675	+
		Sr	pH_*sw*_	Linear	0.0261	0.204	+
		U	pH_*sw*_	Para	0.0082	0.674	+
Tropical urchin	Echinodermata	B	pH_*sw*_	Linear	<0.00001	0.905	+
		Ba	pH_*sw*_	Para	<0.00001	0.833	−
			***pH***_***cf***_	***Para***	***0.0086***	***0.502***	**−**
		***Sr***	***pH***_***cf***_	***Para***	***0.000357***	***0.791***	**−**
		U	pH_*sw*_	Para	<0.0001	0.982	+
Temperate urchin	Echinodermata	Ba	pH_*sw*_	Linear	0.147	0.33	−
Coralline red alga	Rhodophyta	B	pH_*sw*_	Para	0.00194	0.695	−
			***pH***_***cf***_	***Linear***	***0.0038***	***0.681***	**+**
		***Ba***	***pH***_***cf***_	***Para***	***0.0119***	***0.472***	**−**
		Mg	pH_*cf*_	Linear	0.0306	0.44	+
		Na	pH_*sw*_	Linear	0.0379	0.3	+
		Zn	pH_*sw*_	Linear*	<0.00001	0.663	−
			***pH***_***cf***_	***Para***	***0.000377***	***0.529***	**−**
		***U***	***pH***_***cf***_	***Linear***	***0.0161***	***0.528***	**−**
Serpulid worm	Annelida	Mg	pH_*sw*_	Para	0.0151	0.519	−
		Zn	pH_*sw*_	Para	0.0129	0.301	−
		Sr	pH_*sw*_	Para	0.0114	0.285	+
		***B***	***pH***_***cf***_	***Linear***	***0.0147***	***0.447***	**+**
Temperate coral	Cnidaria	Li	pH_*sw*_	Linear	0.00129	0.161	−
		Zn	pH_*sw*_	Linear	0.0000893	0.0884	−
		***Cd***	***pH***_***cf***_	***Para***	***0.0125***	***0.5***	**+**
		U	pH_*sw*_	Para	0.0356	0.0792	+

**TABLE 6 |:** Summary of the simplest (X/Ca vs. Mg/Ca) as well as the final GAMs (bolded) as determined by likelihood ratio tests results for the X/Ca vs. Mg/Ca scatterplots (Figure 2).

Elemental ratio	Formula	Bootstrapped *R*^2^	95% confidence interval (lower, higher)	% deviance explained	Model likelihood	*n*
Li/Ca	Li.Ca ~ Mg.Ca	0.65	0.56, 0.72	64.60%	678.43	181
	Li.Ca ~ Mg.Ca * Phylum + Carbonate.Material	0.96	0.93, 0.97	96%	482.14	181
B/Ca	B.Ca ~ Mg.Ca	0.06	0.02, 0.10	5.54%	1302	188
	B.Ca ~ Mg.Ca * Phylum	0.98	0.98, 0.99	98.20%	927.98	188
Zn/Ca	Zn.Ca ~ Mg.Ca	0.03	0.01, 0.05	2.89%	1928.7	279
	Zn.Ca ~ Mg.Ca + Phylum	0.62	0.49, 0.70	61.70%	1799	279
Sr/Ca	Sr.Ca ~ Mg.Ca	n.s.				
	Sr.Ca ~ Mg.Ca * Phylum + Carbonate.Material	0.99	0.99, 0.99	99%	85.993	239
Cd/Ca	Cd.Ca ~ Mg.Ca	0.72	0.51, 0.83	71.90%	−12.355	148
	Cd.Ca ~ Carbonate.Material	0.51	0.39, 0.60	50.90%	29.034	148
Ba/Ca	Ba.Ca ~ Mg.Ca	0.17	0.10, 0.27	17.00%	1281.5	270
	Ba.Ca ~ Mg.Ca * Phylum	0.69	0.54, 0.79	68.70%	1149.9	270
U/Ca	U.Ca ~ Mg.Ca	n.s.				
	U.Ca ~ Mg.Ca * Phylum + Carbonate.Material	0.93	0.87, 0.95	93%	1063.6	175
Na/Ca	Na.Ca ~ Mg.Ca	n.s.				
	Na.Ca ~ Mg.Ca * Phylum + Carbonate. Material	0.39	0.15, 0.56	38.70%	383.96	121

In the case of Cd/Ca, the simplest model was also determined to be the most likely. Model fit statistics have been bootstrapped over 1,000 iterations. The models tested explored the influence of considering grouping the data by phylum and/or mineralogy. Interactive model components are denoted by an asterisk (*) while additive model components are denoted by a plus sign. Note that “Phylum” here does not account for the phylogenetic relatedness of the species as we do in Figure 7 and Table 2 and Supplementary Table 9 (see methods 2.5.2). For the full list of candidate models, see Supplementary Table 4, and model comparisons in Supplementary Table 5. Models for Cd/Ca and Na/Ca may not be accurate due to the large amount of data missing for five and seven organisms, respectively.

**TABLE 7 |:** Estimates of phylogenetic signal for X/Ca ratios.

Elemental Ratio	Lambda
**Multivariate**	
All	0.99
Li/Ca, Mg/Ca, Zn/Ca, and Sr/Ca	0.895
Li/Ca, Mg/Ca, Sr/Ca	0.898
Mg/Ca and Sr/Ca	0.925
**Univariate**	
Li/Ca	0
B/Ca	0.488
Mg/Ca	0.803
Zn/Ca	0
Sr/Ca	0.835
Cd/Ca	0.665
Ba/Ca	0
U/Ca	0.889
Na/Ca	0.516

Multivariate analyses were conducted using all and combinations of the X/Ca ratios together, as based on a phylogenetic principal component analysis in phytools (Revell, 2012; row = “All”). Univariate analyses estimated phylogenetic signals for individual X/Ca ratios using the phylosig function also in phytools.

## Data Availability

The original contributions presented in the study are included in the article/Supplementary Material, further inquiries can be directed to the corresponding author/s.

## Footnotes

1
https://phylot.biobyte.de
