# Supplementary material for: Patterns of Element Incorporation in Calcium Carbonate Biominerals Recapitulate Phylogeny for a Diverse Range of Marine Calcifiers
Source: Front Earth Sci (Lausanne). Author manuscript; Available in PMC 2022 Nov 14. (PMC9645792; doi:10.3389/feart.2021.641760)
Supplement: Data and supporting figures [file NIHMS1821844-supplement-Data_and_supporting_figures.pdf]

Supplementary Information

Table of Contents

I. Culture experiment growth rate and carbonate chemistry data 3 – 7
II. Summary of elemental ratio measurements 8 – 9
III. Summary of generalized additive models (GAMs)
- A. Complete list of candidate models 9 – 14
- B. Table of likelihood ratio test results 15 – 23
- C. Diagnostic plots for final models 24 – 31
- D. Figures showing final model components 32

IV. Description of inorganic partition coefficient selection 33 – 38
V. Scatterplot arrays of elemental ratios versus carbonate chemistry and other measured parameters 39 – 75
- A. American Lobster (*Homarus americanus*) 40 – 41
- B. American Oyster (*Crassostrea virginica*) 42 – 43
- C. Bay Scallop (*Argopecten irradians*) 44 – 45
- D. Blue Crab (*Callinectes sapidus*) 46 – 47
- E. Blue Mussel (*Mytilus edulis*) 48 – 49
- F. Conch (*Strombus alatus*) 50 – 51
- G. Temperate Coral (*Oculina arbuscula*) 52 – 53
- H. Coralline Red Algae (*Neogoniolithon spectabile*) 54 – 55
- I. Gulf Shrimp (*Penaeus plebejus*) 56 – 57
- J. Halimeda Green Algae (*Halimeda incrassata*) 58 – 59
- K. Hard Clam (*Mercenaria mercenaria*) 60 – 61
- L. Limpet (*Crepidula fornicata*) 62 – 63
- M. Pencil Urchin (*Eucidaris tribuloides*) 64 – 65
- N. Periwinkle (*Littorina littorea*) 66 – 67
- O. Purple Urchin (*Arbacia punctulata*) 68 – 69
- P. Serpulid Worm (*Hydroides crucigera*) 70 – 71
- Q. Soft Clam (*Mya arenaria*) 72 – 73
- R. Whelk (*Urosalpinx cinerea*) 74 – 75

VI. Links to GitHub repositories containing the code used to generate plots 76
- A. Element-to-calcium ratio boxplots
- B. Scatterplots of element-to-calcium ratios vs. carbonate chemistry and other measured parameters with linear fits
- C. Scatterplots of element-to-calcium ratios vs. carbonate chemistry and other measured parameters with quadratic fits
- D. Akaike Information Criterion (AIC) analysis: comparisons between linear and quadratic fits
- E. Non-metric multidimensional scaling (NMDS) plots

VII. Phylogenetic analysis
- A. Methods and results of phylogenetic trees 77 – 79
- B. Link to Nexus file for Time-calibrated phylogenetic tree 79
- C. Additional phylogenetic signal results 79 – 80
- D. Tabulated results of relative contribution comparison 80 – 81

VIII.	List of Supplementary Files	82
IX.	References	83 – 85

I. Culture experiment growth rate and carbonate chemistry data

Here we reproduce data from Ries et al. (2009) for the carbonate chemistry of culture water and organismal net calcification rates as determined by the buoyant weight method.

Table S1. Modified from Ries *et al.* (2009). Tabulated information of the carbonate chemistry parameters in the culture tanks the organisms were grown in. Each group of organisms was grown at four different pCO_2 conditions for the original experiment.

**Tank**	**pCO_2(SD)**	**Sal(SD)**	**Temp(SD)**	**pH(SD)**	**DIC(SD)***
Lobster/Crab/Shrimp (I)	409(5.66)	32.0(0.169)	24.9(0.179)	8.03(0.0562)	1678(139)
Lobster/Crab/Shrimp (II)	606(7.26)	31.8(0.148)	25.0(0.141)	7.85(0.0962)	1732(119)
Lobster/Crab/Shrimp (III)	903(11.74)	32.1(0.969)	25.0(0.152)	7.72(0.0575)	1744(155)
Lobster/Crab/Shrimp (IV)	2856(53.73)	31.9(0.187)	25.1(0.114)	7.31(0.0348)	1860(169)
Conch/Limpet/Periwinkle (I)	409(5.66)	31.8(0.088)	25.1(0.110)	8.09(0.0304)	1568(101)
Conch/Limpet/Periwinkle (II)	606(7.26)	31.8(0.228)	24.9(0.152)	8.00(0.0719)	1634(147)
Conch/Limpet/Periwinkle (III)	903(11.74)	31.9(0.191)	24.9(0.148)	7.86(0.0734)	1733(78)
Conch/Limpet/Periwinkle (IV)	2856(53.73)	31.7(0.110)	24.9(0.134)	7.31(0.0348)	2097(111)
Coralline red alga/Halimeda (I)	409(5.66)	31.8(0.207)	25.0(0.055)	8.19(0.0317)	1738(50)
Coralline red alga/Halimeda (II)	606(7.26)	31.7(0.118)	25.0(0.152)	8.05(0.0604)	1786(101)
Coralline red alga/Halimeda (III)	903(11.74)	31.5(0.155)	25.1(0.164)	7.91(0.0286)	1903(46)
Coralline red alga/Halimeda (IV)	2856(53.73)	31.8(0.258)	24.9(0.130)	7.49(0.0216)	2350(33)
Pencil urchin/Purple urchin (I)	409(5.66)	31.9(0.269)	25.1(0.122)	8.04(0.0574)	1563(127)
Pencil urchin/Purple urchin (II)	606(7.26)	31.8(0.099)	25.0(0.152)	7.90(0.0510)	1623(145)
Pencil urchin/Purple urchin (III)	903(11.74)	31.7(0.218)	24.9(0.114)	7.77(0.0241)	1707(108)
Pencil urchin/Purple urchin (IV)	2856(53.73)	31.7(0.218)	25.0(0.148)	7.36(0.0328)	1921(73)
Coral/Serpulid worm (I)	409(5.66)	31.7(0.208)	25.0(0.191)	8.11(0.0628)	1738(47)
Coral/Serpulid worm (II)	606(7.26)	31.6(0.353)	24.9(0.129)	8.03(0.0376)	1824(32)
Coral/Serpulid worm (III)	903(11.74)	31.7(0.426)	24.9(0.141)	7.85(0.0519)	1907(30)
Coral/Serpulid worm (IV)	2856(53.73)	31.5(0.633)	25.2(0.058)	7.48(0.0332)	2070(40)
Clams/Mussel/Scallop/Oyster (I)	409(5.66)	32.1(0.288)	25.1(0.148)	8.15(0.0404)	1598(112)

Clams/Mussel/Scallop/Oyster (II)	606(7.26)	31.7(0.199)	25.1(0.110)	8.02(0.0803)	1684(126)
Clams/Mussel/Scallop/Oyster (III)	903(11.74)	31.9(0.251)	25.0(0.158)	7.83(0.0452)	1749(87)
Clams/Mussel/Scallop/Oyster (IV)	2856(53.73)	31.9(0.240)	25.0(0.084)	7.45(0.0370)	2071(51)

*Calculated

Table S2. Modified from Ries *et al.* (2009). Tabulated information about the net calcification rate results calculated from the buoyant weighing technique. Briefly, organisms were suspended by an aluminum wire hanging from a bottom-loading scale in an aquarium with filtered seawater. Net calcification rates were calculated as the %-weight difference between the organisms' initial and final buoyant weights. A calibration curve of final buoyant weight vs. dry $CaCO_3$ weight was used to estimate the final dry $CaCO_3$ weight of the organisms.

**Organism**	**Scientific name**	**pCO_2 (SD)**	**Avg net calcification rate (SD)**
			wt-%/60-day
American lobster	*Homarus americanus*	409(5.66)	353.0(91.0)
American lobster	*Homarus americanus*	606(7.26)	349.5(39.6)
American lobster	*Homarus americanus*	903(11.74)	376.3(58.6)
American lobster	*Homarus americanus*	2856(53.73)	606.1(164.6)
Blue crab	*Callinectes sapidus*	409(5.66)	433.8(138.0)
Blue crab	*Callinectes sapidus*	606(7.26)	598.2(116.7)
Blue crab	*Callinectes sapidus*	903(11.74)	600.9(117.3)
Blue crab	*Callinectes sapidus*	2856(53.73)	724.0(109.2)
Gulf shrimp	*Penaeus plebejus*	409(5.66)	15.3(11.4)
Gulf shrimp	*Penaeus plebejus*	606(7.26)	17.3(7.4)
Gulf shrimp	*Penaeus plebejus*	903(11.74)	27.5(9.4)
Gulf shrimp	*Penaeus plebejus*	2856(53.73)	37.8(13.7)
Conch	*Strombus alatus*	409(5.66)	2.0(0.5)
Conch	*Strombus alatus*	606(7.26)	0.8(1.2)
Conch	*Strombus alatus*	903(11.74)	1.2(1.4)
Conch	*Strombus alatus*	2856(53.73)	-3.1(2.0)
Limpet	*Crepidula fornicata*	409(5.66)	12.4(9.5)
Limpet	*Crepidula fornicata*	606(7.26)	22.2(6.8)
Limpet	*Crepidula fornicata*	903(11.74)	33.0(5.0)
Limpet	*Crepidula fornicata*	2856(53.73)	21.2(7.0)
Whelk	*Urosalpinx cinerea*	409(5.66)	2.9(1.9)
Whelk	*Urosalpinx cinerea*	606(7.26)	0.7(1.1)

Whelk	*Urosalpinx cinerea*	903(11.74)	0.7(0.6)
Whelk	*Urosalpinx cinerea*	2856(53.73)	-1.5(0.9)
Periwinkle	*Littorina littorea*	409(5.66)	3.8(2.9)
Periwinkle	*Littorina littorea*	606(7.26)	1.9(1.6)
Periwinkle	*Littorina littorea*	903(11.74)	0.9(2.1)
Periwinkle	*Littorina littorea*	2856(53.73)	-1.2(3.4)
Coralline red algae	*Neogoniolithon spectabile*	409(5.66)	5.7(2.9)
Coralline red algae	*Neogoniolithon spectabile*	606(7.26)	14.5(6.6)
Coralline red algae	*Neogoniolithon spectabile*	903(11.74)	10.7(4.7)
Coralline red algae	*Neogoniolithon spectabile*	2856(53.73)	3.6(3.5)
Halimeda green alga	*Halimeda incrassata*	409(5.66)	23.9(12.5)
Halimeda green alga	*Halimeda incrassata*	606(7.26)	43.9(7.8)
Halimeda green alga	*Halimeda incrassata*	903(11.74)	25.4(3.0)
Halimeda green alga	*Halimeda incrassata*	2856(53.73)	6.0(2.6)
Pencil urchin	*Eucidaris tribuloides*	409(5.66)	8.7(6.4)
Pencil urchin	*Eucidaris tribuloides*	606(7.26)	5.6(2.7)
Pencil urchin	*Eucidaris tribuloides*	903(11.74)	5.6(3.1)
Pencil urchin	*Eucidaris tribuloides*	2856(53.73)	-18.3(7.3)
Purple urchin	*Arbacia punctulata*	409(5.66)	8.2(5.7)
Purple urchin	*Arbacia punctulata*	606(7.26)	39.4(5.0)
Purple urchin	*Arbacia punctulata*	903(11.74)	42.8(3.8)
Purple urchin	*Arbacia punctulata*	2856(53.73)	28.5(1.9)
Coral	*Oculina arbuscula*	409(5.66)	11.8(1.2)
Coral	*Oculina arbuscula*	606(7.26)	11.6(1.2)
Coral	*Oculina arbuscula*	903(11.74)	11.1(1.5)
Coral	*Oculina arbuscula*	2856(53.73)	3.8(1.7)
Serpulid worm	*Hydroides crucigera*	409(5.66)	3.5(4.1)
Serpulid worm	*Hydroides crucigera*	606(7.26)	5.5(3.8)

Serpulid worm	*Hydroides crucigera*	903(11.74)	2.3(4.0)
Serpulid worm	*Hydroides crucigera*	2856(53.73)	1.3(3.8)
Hard clam	*Mercenaria mercenaria*	409(5.66)	1.0(0.6)
Hard clam	*Mercenaria mercenaria*	606(7.26)	0.7(0.3)
Hard clam	*Mercenaria mercenaria*	903(11.74)	0.5(0.4)
Hard clam	*Mercenaria mercenaria*	2856(53.73)	-1.4(0.4)
Blue mussel	*Mytilus edulis*	409(5.66)	4.4(2.2)
Blue mussel	*Mytilus edulis*	606(7.26)	3.1(2.5)
Blue mussel	*Mytilus edulis*	903(11.74)	3.6(3.4)
Blue mussel	*Mytilus edulis*	2856(53.73)	3.1(3.2)
Soft clam	*Mya arenaria*	409(5.66)	17.5(3.5)
Soft clam	*Mya arenaria*	606(7.26)	7.7(3.6)
Soft clam	*Mya arenaria*	903(11.74)	0.2(6.9)
Soft clam	*Mya arenaria*	2856(53.73)	-8.1(6.9)
Bay scallop	*Argopecten irradians*	409(5.66)	6.9(2.1)
Bay scallop	*Argopecten irradians*	606(7.26)	6.3(0.8)
Bay scallop	*Argopecten irradians*	903(11.74)	4.8(4.2)
Bay scallop	*Argopecten irradians*	2856(53.73)	2.3(1.9)
Oyster	*Crassostrea virginica*	409(5.66)	1.9(0.3)
Oyster	*Crassostrea virginica*	606(7.26)	1.6(0.3)
Oyster	*Crassostrea virginica*	903(11.74)	1.1(0.3)
Oyster	*Crassostrea virginica*	2856(53.73)	0.3(0.4)

II. Summary of element-to-calcium ratio measurements from this study.

Table S3. Summary of element-to-calcium ratio measurements. "*" denotes an imputed value (see methods and Figure 4).

**Organism**	**Li/Ca (SD)**	**B/Ca (SD)**	**Na/Ca (SD)**	**Mg/Ca (SD)**	**Zn/Ca (SD)**	**Sr/Ca (SD)**	**Cd/Ca (SD)**	**Ba/Ca (SD)**	**U/Ca (SD)**
Temperate Coral	7.30 (0.43)	582.31 (36.53)	23.14*	4.22 (0.36)	4.16 (2.20)	9.61 (0.38)	0.04 (0.06)	15.25 (2.07)	879.07 (123.26)
Halimeda Algae	20.50*	96.42*	11.86 (1.35)	4.50 (3.84)	0.13 (0.10)	9.98 (0.70)	0.344*	27.76 (7.39)	33.15*
Soft Clam	5.41 (0.58)	26.10 (4.35)	22.40*	0.77 (0.15)	2.10 (1.07)	2.50 (0.27)	0.05 (0.07)	7.57 (4.73)	40.47 (19.86)
Conch	5.18 (0.76)	15.62 (2.06)	25.90 (1.74)	0.77 (0.30)	1.50 (0.89)	1.80 (0.28)	0.01 (0.01)	4.43 (2.56)	18.47 (8.69)
Hard Clam (Quahog)	8.40 (0.83)	49.26 (12.23)	22.04*	15.87 (29.04)	98.29 (112.28)	2.27 (0.86)	0.01 (0.00)	1.59 (19.36)	286.75 (227.15)
Limpet	4.87 (0.34)	16.58 (3.50)	22.50 (2.27)	1.11 (0.39)	2.27 (2.33)	1.58 (0.18)	0.01 (0.01)	6.78 (8.19)	58.68 (19.23)
Whelk	5.43 (0.86)	15.96 (2.90)	23.22 (1.51)	0.98 (0.48)	6.52 (10.83)	2.06 (0.22)	0.98 (2.81)	5.21 (5.88)	63.24 (28.87)
Serpulid Worm	49.24 (9.73)	475.05 (56.13)	19.79*	162.24 (22.34)	6.23 (3.21)	5.69 (0.74)	0.59 (0.50)	86.68 (67.65)	504.18 (127.97)
Blue Mussel	7.00 (1.23)	41.25 (10.03)	22.00*	29.00 (30.49)	163.23 (202.26)	2.46 (1.55)	0.03 (0.02)	52.97 (85.99)	6.30 (1.34)
Periwinkle	14.51 (4.33)	2.89 (0.96)	16.74 (2.96)	3.95 (1.52)	6.69 (5.35)	1.16 (0.12)	0.00 (0.00)	1.53 (0.54)	8.68 (5.48)
American Oyster	23.94 (4.40)	101.85 (10.01)	20.98*	10.97 (1.48)	27.92 (15.44)	1.00 (0.12)	0.02 (0.01)	1.02 (0.15)	9.06 (2.52)
Bay Scallop	11.97 (2.54)	32.97 (7.37)	21.88*	19.32 (4.01)	9.59 (6.10)	1.41 (0.07)	0.00 (0.00)	5.82 (2.02)	3.74 (2.03)
American Lobster	30.87*	195.76*	31.45 (16.13)	74.62 (22.82)	730.34 (370.28)	5.21 (0.94)	0.399*	123.95 (49.17)	24.01*
Blue Crab	33.36*	212.37*	20.02 (3.81)	58.26 (6.93)	211.13 (86.27)	5.30 (0.36)	0.413*	44.19 (27.77)	31.54*
Gulf Shrimp	31.39*	198.75*	17.41 (4.63)	51.57 (10.64)	1064.94 (583.68)	5.61 (1.81)	0.415*	70.58 (49.73)	33.58*
Coralline Red Algae	45.40 (3.75)	425.42 (60.88)	15.42 (1.22)	264.42 (11.68)	75.35 (34.44)	3.23 (0.22)	1.37 (0.53)	24.46 (5.56)	140.63 (15.76)
Pencil Urchin	51.63 (1.24)	323.72 (55.84)	20.57 (1.40)	68.45 (7.38)	5.27 (3.10)	2.11 (0.07)	0.43 (0.11)	10.57 (3.11)	13.23 (5.53)
Purple Urchin	57.00 (4.86)	395.40 (15.54)	23.62 (1.36)	76.37 (5.90)	2.12 (1.05)	2.21 (0.09)	0.23 (0.03)	7.25 (2.40)	5.91 (1.01)

III. Summary of generalized additive models (GAMs) from this study.

A. Complete list of candidate models

Table S4. Generalized additive model results. Within the model formulae, "*" and "+" indicate interactive and additive models, respectively. Invalid models are noted with a "n.s." in the % Deviance Explained column. **Bolded** model rows indicate the most likely model determined by lrtest().

Li				
Model	Formula	% Deviance Explained	Model Likelihood	n
li.mod	Li.Ca ~ Mg.Ca	64.60%	678.43	181
li.mod2	Li.Ca ~ Phylum	91.00%	554.36	181
li.mod3	Li.Ca ~ Mg.Ca + Phylum	94.30%	513.65	181
li.mod4	Li.Ca ~ Mg.Ca * Phylum	94.30%	508.04	181
li.mod9	Li.Ca ~ Carbonate.Material	72.10%	656.96	181
li.mod10	Li.Ca ~ Phylum + Carbonate.Material	94.20%	515.28	181
li.mod11	Li.Ca ~ Phylum * Carbonate.Material	94.20%	515.28	181
li.mod12	Li.Ca ~ Carbonate.Material + Phylum	94.20%	515.28	181
li.mod13	Li.Ca ~ Carbonate.Material * Phylum	94.20%	515.28	181
li.mod14	Li.Ca ~ Mg.Ca + Carbonate.Material	78.10%	634.79	181
li.mod15	Li.Ca ~ Mg.Ca * Carbonate.Material	95.60%	489.49	181
**li.mod16**	**Li.Ca ~ Mg.Ca * Phylum + Carbonate.Material**	**96%**	**482.14**	**181**
li.mod17	Li.Ca ~ Mg.Ca + Phylum + Carbonate.Material	95.50%	490.79	181
li.mod18	Li.Ca ~ Mg.Ca * Carbonate.Material + Phylum	95.90%.	482.74	181
li.mod19	Li.Ca ~ Mg.Ca * Carbonate.Material * Phylum	n.s.		
B				
Model	Formula	Deviance Explained	Model Likelihood	n
b.mod	B.Ca ~ Mg.Ca	5.54%	1302	188
b.mod2	B.Ca ~ Phylum	97.80%	950.21	188
b.mod3	B.Ca ~ Mg.Ca + Phylum	97.90%	945.28	188

**b.mod4**	**B.Ca ~ Mg.Ca * Phylum**	**98.20%**	**927.98**	**188**
b.mod9	B.Ca ~ Carbonate.Material	28%	1277	188
b.mod10	B.Ca ~ Phylum + Carbonate.Material	97.80%	947.41	188
b.mod11	B.Ca ~ Phylum * Carbonate.Material	97.80%	947.41	188
b.mod12	B.Ca ~ Carbonate.Material + Phylum	97.80%	947.41	188
b.mod13	B.Ca ~ Carbonate.Material * Phylum	97.80%	947.41	188
b.mod14	B.Ca ~ Mg.Ca + Carbonate.Material	35.20%	1266.9	188
b.mod15	B.Ca ~ Mg.Ca * Carbonate.Material	94.1%	1041.8	188
b.mod16	B.Ca ~ Mg.Ca * Phylum + Carbonate.Material	98.20%	927.548	188
b.mod17	B.Ca ~ Mg.Ca + Phylum + Carbonate.Material	97.90%	944.38	188
b.mod18	B.Ca ~ Mg.Ca * Carbonate.Material + Phylum	98.20%	930.11	188
b.mod19	B.Ca ~ Mg.Ca * Carbonate.Material * Phylum	98.20%	927.51	188
Zn				
Model	Formula	Deviance Explained	Model Likelihood	n
zn.mod	Zn.Ca ~ Mg.Ca	2.89%	1928.7	279
zn.mod2	Zn.Ca ~ Phylum	59.10%	1863.9	282
**zn.mod3**	**Zn.Ca ~ Mg.Ca + Phylum**	**61.70%**	**1799**	**279**
zn.mod4	Zn.Ca ~ Mg.Ca * Phylum	n.s.		
zn.mod9	Zn.Ca ~ Carbonate.Material	27.60%	1944.4	282
zn.mod10	Zn.Ca ~ Phylum + Carbonate.Material	59.10%	1863.8	282
zn.mod11	Zn.Ca ~ Phylum * Carbonate.Material	59.10%	1863.8	282
zn.mod12	Zn.Ca ~ Carbonate.Material + Phylum	59.10%	1863.8	282
zn.mod13	Zn.Ca ~ Carbonate.Material * Phylum	59.10%	1863.8	282
zn.mod14	Zn.Ca ~ Mg.Ca + Carbonate.Material	32.80%	1877.3	279
zn.mod15	Zn.Ca ~ Mg.Ca * Carbonate.Material	34.70%	1873.3	279
zn.mod16	Zn.Ca ~ Mg.Ca * Phylum + Carbonate.Material	n.s.		
zn.mod17	Zn.Ca ~ Mg.Ca + Phylum + Carbonate.Material	61.80%	1798.8	279

zn.mod18	Zn.Ca ~ Mg.Ca * Carbonate.Material + Phylum	61.90%	1798.3	279
zn.mod19	Zn.Ca ~ Mg.Ca * Carbonate.Material * Phylum	n.s.		
Sr				
Model	Formula	Deviance Explained	Model Likelihood	n
sr.mod	Sr.Ca ~ Mg.Ca	n.s.		
sr.mod2	Sr.Ca ~ Phylum	97.00%	228.07	254
sr.mod3	Sr.Ca ~ Mg.Ca + Phylum	98.00%	167.86	239
sr.mod4	Sr.Ca ~ Mg.Ca * Phylum	98.70%	115.35	239
sr.mod9	Sr.Ca ~ Carbonate.Material	25.70%	634.92	254
sr.mod10	Sr.Ca ~ Phylum + Carbonate.Material	97.40%	207.7	254
sr.mod11	Sr.Ca ~ Phylum * Carbonate.Material	97.40%	207.7	254
sr.mod12	Sr.Ca ~ Carbonate.Material + Phylum	97.40%	207.7	254
sr.mod13	Sr.Ca ~ Carbonate.Material * Phylum	97.40%	207.7	254
sr.mod14	Sr.Ca ~ Mg.Ca + Carbonate.Material	33.30%	584.93	239
sr.mod15	Sr.Ca ~ Mg.Ca * Carbonate.Material	77.60%	454.81	239
**sr.mod16**	**Sr.Ca ~ Mg.Ca * Phylum + Carbonate.Material**	**99%**	**85.993**	**239**
sr.mod17	Sr.Ca ~ Mg.Ca + Phylum + Carbonate.Material	98.50%	133.55	239
sr.mod18	Sr.Ca ~ Mg.Ca * Carbonate.Material + Phylum	98.90%	99.973	239
sr.mod19	Sr.Ca ~ Mg.Ca * Carbonate.Material * Phylum	99%	85.891	239
Cd				
Model	Formula	Deviance Explained	Model Likelihood	n
cd.mod	Cd.Ca ~ Mg.Ca	71.90%	-12.355	148
cd.mod2	Cd.Ca ~ Phylum	75.20%	-21.578	148
cd.mod3	Cd.Ca ~ Mg.Ca + Phylum	75.50%	-22.599	148
cd.mod4	Cd.Ca ~ Mg.Ca * Phylum	76.30%	-24.999	148
**cd.mod9**	**Cd.Ca ~ Carbonate.Material**	**50.90%**	**29.034**	**148**
cd.mod10	Cd.Ca ~ Phylum + Carbonate.Material	76.10%	-24.375	148

cd.mod11	Cd.Ca ~ Phylum * Carbonate.Material	76.10%	-24.375	148
cd.mod12	Cd.Ca ~ Carbonate.Material + Phylum	76.10%	-24.375	148
cd.mod13	Cd.Ca ~ Carbonate.Material * Phylum	76.10%	-24.375	148
cd.mod14	Cd.Ca ~ Mg.Ca + Carbonate.Material	73.10%	-18.025	148
cd.mod15	Cd.Ca ~ Mg.Ca * Carbonate.Material	75.70%	-22.949	148
cd.mod16	Cd.Ca ~ Mg.Ca * Phylum + Carbonate.Material	77.20%	-27.727	148
cd.mod17	Cd.Ca ~ Mg.Ca + Phylum + Carbonate.Material	76.40%	-25.156	148
cd.mod18	Cd.Ca ~ Mg.Ca * Carbonate.Material + Phylum	77.20%	-27.759	148
cd.mod19	Cd.Ca ~ Mg.Ca * Carbonate.Material * Phylum	77.60%	-29.046	148
Ba				
Model	Formula	Deviance Explained	Model Likelihood	n
ba.mod	Ba.Ca ~ Mg.Ca	17.00%	1281.5	270
ba.mod2	Ba.Ca ~ Phylum	57.80%	1265.2	285
ba.mod3	Ba.Ca ~ Mg.Ca + Phylum	64.30%	1167.5	270
**ba.mod4**	**Ba.Ca ~ Mg.Ca * Phylum**	**68.70%**	**1149.9**	**270**
ba.mod9	Ba.Ca ~ Carbonate.Material	21.30%	1354.1	285
ba.mod10	Ba.Ca ~ Phylum + Carbonate.Material	57.90%	1265	285
ba.mod11	Ba.Ca ~ Phylum * Carbonate.Material	57.90%	1265	285
ba.mod12	Ba.Ca ~ Carbonate.Material + Phylum	57.90%	1265	285
ba.mod13	Ba.Ca ~ Carbonate.Material * Phylum	57.90%	1265	285
ba.mod14	Ba.Ca ~ Mg.Ca + Carbonate.Material	22.50%	1272.3	270
ba.mod15	Ba.Ca ~ Mg.Ca * Carbonate.Material	35.20%	1247.9	270
ba.mod16	Ba.Ca ~ Mg.Ca * Phylum + Carbonate.Material	68.80%	1149.4	270
ba.mod17	Ba.Ca ~ Mg.Ca + Phylum + Carbonate.Material	65.60%	1162.6	270
ba.mod18	Ba.Ca ~ Mg.Ca * Carbonate.Material + Phylum	66.50%	1159	270
ba.mod19	Ba.Ca ~ Mg.Ca * Carbonate.Material * Phylum	n.s.		
U				

Model	Formula	Deviance Explained	Model Likelihood	n
u.mod	U.Ca ~ Mg.Ca	n.s.		
u.mod2	U.Ca ~ Phylum	91.60%	1078.5	175
u.mod3	U.Ca ~ Mg.Ca + Phylum	92.00%	1074	175
u.mod4	U.Ca ~ Mg.Ca * Phylum	92.60%	1067.5	175
u.mod9	U.Ca ~ Carbonate.Material	37.70%	1254.3	175
u.mod10	U.Ca ~ Phylum + Carbonate.Material	92.30%	1071.5	175
u.mod11	U.Ca ~ Phylum * Carbonate.Material	92.30%	1071.5	175
u.mod12	U.Ca ~ Carbonate.Material + Phylum	92.30%	1071.5	175
u.mod13	U.Ca ~ Carbonate.Material * Phylum	92.30%	1071.5	175
u.mod14	U.Ca ~ Mg.Ca + Carbonate.Material	41.30%	1249.1	175
u.mod15	U.Ca ~ Mg.Ca * Carbonate.Material	88.50%	1106.2	175
**u.mod16**	**U.Ca ~ Mg.Ca * Phylum + Carbonate.Material**	**93%**	**1063.6**	**175**
u.mod17	U.Ca ~ Mg.Ca + Phylum + Carbonate.Material	92.40%	1070.1	175
u.mod18	U.Ca ~ Mg.Ca * Carbonate.Material + Phylum	92.70%	1066.7	175
u.mod19	U.Ca ~ Mg.Ca * Carbonate.Material * Phylum	n.s.		
Na				
Model	Formula	Deviance Explained	Model Likelihood	n
na.mod	Na.Ca ~ Mg.Ca	n.s.		
na.mod2	Na.Ca ~ Phylum	19.40%	409.7	124
na.mod3	Na.Ca ~ Mg.Ca + Phylum	27.60%	394.03	121
na.mod4	Na.Ca ~ Mg.Ca * Phylum	38.20%	384.39	121
na.mod9	Na.Ca ~ Carbonate.Material	4.39%	420.27	124
na.mod10	Na.Ca ~ Phylum + Carbonate.Material	26.40%	404.05	124
na.mod11	Na.Ca ~ Phylum * Carbonate.Material	26.40%	404.05	124
na.mod12	Na.Ca ~ Carbonate.Material + Phylum	26.40%	404.05	124
na.mod13	Na.Ca ~ Carbonate.Material * Phylum	26.40%	404.05	124
na.mod14	Na.Ca ~ Mg.Ca + Carbonate.Material	9.64%	407.41	121
na.mod15	Na.Ca ~ Mg.Ca * Carbonate.Material	16.60%	402.55	121

**na.mod16**	**Na.Ca ~ Mg.Ca * Phylum + Carbonate.Material**	**38.70%**	**383.96**	**121**
na.mod17	Na.Ca ~ Mg.Ca + Phylum + Carbonate.Material	36%	386.5	121
na.mod18	Na.Ca ~ Mg.Ca * Carbonate.Material + Phylum	37.30%	385.27	121
na.mod19	Na.Ca ~ Mg.Ca * Carbonate.Material * Phylum	n.s.		

B. Table of likelihood ratio test results

Table S5. Tabulated p-value results of likelihood ratio tests comparing candidate models.. Significant p-values ($p < 0.05$) infer that the models do not provide equal outcomes, and suggest that the better model is the one with a higher Model Likelihood score. Cells with “-” occur when there are missing Mg/Ca values for some of the replicates that have X/Ca data (X = Li, B, Zn, etc.) and thus causing models assessing phylum and/or mineralogy without Mg/Ca to have a different n and preventing it from being compared to the other models. Blank cells means that the model was invalid ($p > 0.05$). The cell color indicates which model performs better according to the log-likelihood ratio test. A white cell indicates that the model assigned to that column (i.e. the column label across the top) performed better than the cell assigned to that row (i.e. the row labels along the left side of the table); a gray cells indicates that the model assigned to that column performed worse than the model assigned to that row. A red cell indicates that the models were indistinguishable ($p > 0.05$) and that the model with the higher Model Likelihood should be used.

Li														
	li.mod	li.mod2	li.mod3	li.mod4	li.mod9	li.mod10	li.mod11	li.mod12	li.mod13	li.mod14	li.mod15	li.mod16	li.mod17	li.mod18	li.mod19
li.mod		2.20E-16	2.20E-16	2.20E-16	4.741E-10	2.20E-16	2.20E-16	2.20E-16	2.20E-16	4.96E-07	2.20E-16	2.20E-16	2.20E-16	2.20E-16	
li.mod2	2.20E-16		2.20E-16	2.20E-16	2.20E-16	2.20E-16	2.20E-16	2.20E-16	2.20E-16	2.20E-16	2.20E-16	2.20E-16	2.20E-16	2.20E-16	
li.mod3	2.20E-16	2.20E-16		0.0241	2.20E-16	7.10E-1	7.10E-1	7.10E-1	7.10E-1	2.20E-16	8.84E-13	1.05E-11	1.96E-10	1.38E-12	
li.mod4	2.20E-16	2.20E-16	2.20E-16		2.20E-16	4.91E-03	4.91E-03	0.004912	0.004912	2.20E-16	8.8E-9	5.6E-12	3.2E-8	1.1E-12	
li.mod9	4.741E-10	2.20E-16	2.20E-16	2.20E-16		2.20E-16	2.20E-16	2.20E-16	2.20E-16	1.20E-14	2.20E-16	2.20E-16	2.20E-16	2.20E-16	
li.mod10	2.20E-16	2.20E-16	7.10E-1	2.20E-16	2.20E-16		1.00E+00	1.00E+00	1.00E+00	2.20E-16	2.20E-16	1.02E-12	5.30E-12	1.06E-13	
li.mod11	2.20E-16	2.20E-16	7.10E-1	2.20E-16	2.20E-16	1.00E+00		1.00E+00	1.00E+00	2.20E-16	2.20E-16	1.02E-12	5.30E-12	1.06E-13	
li.mod12	2.20E-16	2.20E-16	7.10E-1	2.20E-16	2.20E-16	1.00E+00	1.00E+00		1.00E+00	2.20E-16	2.20E-16	1.02E-12	5.30E-12	1.06E-13	
li.mod13	2.20E-16	2.20E-16	7.10E-1	2.20E-16	2.20E-16	1.00E+00	1.00E+00	1.00E+00		2.20E-16	2.20E-16	1.02E-12	5.30E-12	1.06E-13	
li.mod14	2.20E-16	2.20E-16	2.20E-16	2.20E-16	2.70E-11	2.20E-16	2.20E-16	2.20E-16	2.20E-16		2.20E-16	2.20E-16	2.20E-16	2.20E-16	
li.mod15	2.20E-16	2.20E-16	3.2E-11	8.8E-9	2.20E-16	6.8E-13	6.8E-13	6.8E-13	6.8E-13	2.20E-16		0.005	0.006757	0.01801	
li.mod16	2.20E-16	2.20E-16	1.08E-11	5.6E-12	2.20E-16	6.0E-13	6.0E-13	6.0E-13	6.0E-13	2.20E-16	0.005		0.001468	0.2011	
li.mod17	2.20E-16	2.20E-16	1.2E-10	3.2E-8	2.20E-16	2.6E-12	2.6E-12	2.6E-12	2.6E-12	2.20E-16	2.20E-16	0.002		0.0002428	
li.mod18	2.20E-16	2.20E-16	5.1E-12	1.1E-12	2.20E-16	2.5E-13	2.5E-13	2.5E-13	2.5E-13	2.20E-16	0.004	0.27	0.001		

li.mod19															
B															
	b.mod	b.mod2	b.mod3	b.mod4	b.mod9	b.mod10	b.mod11	b.mod12	b.mod13	b.mod14	b.mod15	b.mod16	b.mod17	b.mod18	b.mod19
b.mod		2.20E-16	2.20E-16	2.20E-16	1.09E-11	2.20E-16	2.20E-16	2.20E-16	2.20E-16	2.90E-15	4.99E-16	2.20E-16	2.20E-16	2.20E-16	2.20E-16
b.mod2	2.20E-16		1.70E-03	1.87E-08	2.20E-16	6.00E-01	6.00E-01	6.00E-01	6.00E-01	2.20E-16	2.20E-16	1.20E-07	0.009	4.16E-07	7.80E-07
b.mod3	2.20E-16	1.70E-03		5.58E-07	2.20E-16	4.00E-02	4.00E-02	4.00E-02	4.00E-02	2.20E-16	2.20E-16	3.48E-07	0.4059	1.26E-05	2.13E-05
b.mod4	2.20E-16	1.87E-08	5.58E-07		2.20E-16	1.85E-08	1.85E-08	1.85E-08	1.85E-08	2.20E-16	2.20E-16	0.6477	7.51E-08	0.03902	0.9202
b.mod9	1.09E-11	2.20E-16	2.20E-16	2.20E-16		2.20E-16	2.20E-16	2.20E-16	2.20E-16	6.81E-06	2.20E-16	2.20E-16	2.20E-16	2.20E-16	2.20E-16
b.mod10	2.20E-16	6.00E-01	4.00E-02	1.85E-08	2.20E-16		1.00E+00	1.00E+00	1.00E+00	< 2.2e-16	< 2.2e-16	1.69E-07	0.01384	5.59E-07	1.38E-06
b.mod11	2.20E-16	6.00E-01	4.00E-02	1.85E-08	2.20E-16	1.00E+00		1.00E+00	1.00E+00	< 2.2e-16	< 2.2e-16	1.69E-07	0.01384	5.59E-07	1.38E-06
b.mod12	2.20E-16	6.00E-01	4.00E-02	1.85E-08	2.20E-16	1.00E+00	1.00E+00		1.00E+00	< 2.2e-16	< 2.2e-16	1.69E-07	0.01384	5.59E-07	1.38E-06
b.mod13	2.20E-16	6.00E-01	4.00E-02	1.85E-08	2.20E-16	1.00E+00	1.00E+00	1.00E+00		< 2.2e-16	< 2.2e-16	1.69E-07	0.01384	5.59E-07	1.38E-06
b.mod14	2.90E-15	2.20E-16	2.20E-16	2.20E-16	6.81E-06	< 2.2e-16	< 2.2e-16	< 2.2e-16	< 2.2e-16		< 2.2e-16	< 2.2e-16	< 2.2e-16	< 2.2e-16	< 2.2e-16
b.mod15	4.99E-16	2.20E-16	2.20E-16	2.20E-16	2.20E-16	< 2.2e-16	< 2.2e-16	< 2.2e-16	< 2.2e-16	< 2.2e-16		< 2.2e-16	< 2.2e-16	< 2.2e-16	< 2.2e-16
b.mod16	2.20E-16	1.20E-07	3.48E-07	0.6477	2.20E-16	1.69E-07	1.69E-07	1.69E-07	1.69E-07	< 2.2e-16	< 2.2e-16		8.67E-07	2.35E-02	0.9699
b.mod17	2.20E-16	0.009	0.4059	7.51E-08	2.20E-16	0.01384	0.01384	0.01384	0.01384	< 2.2e-16	< 2.2e-16	8.67E-07		2.78E-06	7.55E-06
b.mod18	2.20E-16	4.16E-07	1.26E-05	0.03902	2.20E-16	5.59E-07	5.59E-07	5.59E-07	5.59E-07	< 2.2e-16	< 2.2e-16	2.35E-02	2.78E-06		0.1584
b.mod19	2.20E-16	7.80E-07	2.13E-05	0.9202	2.20E-16	1.38E-06	1.38E-06	1.38E-06	1.38E-06	< 2.2e-16	< 2.2e-16	0.9699	7.55E-06	0.1584	
Zn															

	zn.mod	zn.mod2	zn.mod3	zn.mod4	zn.mod9	zn.mod10	zn.mod11	zn.mod12	zn.mod13	zn.mod14	zn.mod15	zn.mod16	zn.mod17	zn.mod18	zn.mod19
zn.mod		-	< 2.2E-16		-	-	-	-	-	< 2.2E-16	< 2.2E-16		< 2.2E-16	< 2.2E-16	
zn.mod2	-		-		< 2.2E-16	9.96E-01	9.96E-01	9.96E-01	9.69E-01	-	-		-	-	
zn.mod3	< 2.2E-16	-			-	-	-	-	-	2.20E-16	2.20E-16		7.70E-01	9.10E-01	
zn.mod4															
zn.mod9	-	< 2.2E-16	-			< 2.2E-16	< 2.2E-16	< 2.2E-16	< 2.2E-16	-	-		-	-	
zn.mod10	-	9.96E-01	-		< 2.2E-16		1.00E+00	1.00E+00	1.00E+00	-	-		-	-	
zn.mod11	-	9.96E-01	-		< 2.2E-16	1.00E+00		1.00E+00	1.00E+00	-	-		-	-	
zn.mod12	-	9.96E-01	-		< 2.2E-16	1.00E+00	1.00E+00		1.00E+00	-	-		-	-	
zn.mod13	-	9.69E-01	-		< 2.2E-16	1.00E+00	1.00E+00	1.00E+00		-	-		-	-	
zn.mod14	< 2.2E-16	-	2.20E-16		-	-	-	-	-		4.50E-02		< 2.2E-16	< 2.2E-16	
zn.mod15	< 2.2E-16	-	2.20E-16		-	-	-	-	-	4.50E-02			< 2.2E-16	< 2.2E-16	
zn.mod16		-			-	-	-	-	-						
zn.mod17	< 2.2E-16	-	7.70E-01		-	-	-	-	-	< 2.2E-16	< 2.2E-16			7.90E-01	
zn.mod18	< 2.2E-16	-	9.10E-01		-	-	-	-	-	< 2.2E-16	< 2.2E-16		7.90E-01		
zn.mod19															
Sr															
	sr.mod	sr.mod2	sr.mod3	sr.mod4	sr.mod9	sr.mod10	sr.mod11	sr.mod12	sr.mod13	sr.mod14	sr.mod15	sr.mod16	sr.mod17	sr.mod18	sr.mod19

sr.mod															
sr.mod2			-	-	< 2.2E-16	1.43E-09	1.43E-09	1.43E-09	1.43E-09	-	-	-	-	-	-
sr.mod3		-		< 2.2E-16	-	-	-	-	-	< 2.2E-16	< 2.2E-16	< 2.2E-16	1.27E-15	< 2.2E-16	< 2.2E-16
sr.mod4		-	< 2.2E-16		-	-	-	-	-	< 2.2E-16	< 2.2E-16	1.78E-13	2.39E-07	1.34E-09	4.89E-12
sr.mod9		< 2.2E-16	-	-		< 2.2E-16	< 2.2E-16	< 2.2E-16	< 2.2E-16	-	-	-	-	-	-
sr.mod10		1.43E-09	-	-	< 2.2E-16		1	1	1	-	-	-	-	-	-
sr.mod11		1.43E-09	-	-	< 2.2E-16	1		1	1	-	-	-	-	-	-
sr.mod12		1.43E-09	-	-	< 2.2E-16	1	1		1	-	-	-	-	-	-
sr.mod13		1.43E-09	-	-	< 2.2E-16	1	1	1		-	-	-	-	-	-
sr.mod14		-	< 2.2E-16	< 2.2E-16	-	-	-	-	-		< 2.2E-16	< 2.2E-16	< 2.2E-16	< 2.2E-16	< 2.2E-16
sr.mod15		-	< 2.2E-16	< 2.2E-16	-	-	-	-	-	< 2.2E-16		< 2.2E-16	< 2.2E-16	< 2.2E-16	< 2.2E-16
sr.mod16		-	< 2.2E-16	1.78E-13	-	-	-	-	-	< 2.2E-16	< 2.2E-16		< 2.2E-16	6.65E-05	0.9033
sr.mod17		-	1.27E-15	2.39E-07	-	-	-	-	-	< 2.2E-16	< 2.2E-16	< 2.2E-16		8.98E-16	< 2.2E-16
sr.mod18		-	< 2.2E-16	1.34E-09	-	-	-	-	-	< 2.2E-16	< 2.2E-16	6.65E-05	8.98E-16		0.0004876
sr.mod19		-	< 2.2E-16	4.89E-12	-	-	-	-	-	< 2.2E-16	< 2.2E-16	0.9033	< 2.2E-16	0.0004876	
Cd															
	cd.mod	cd.mod2	cd.mod3	cd.mod4	cd.mod9	cd.mod10	cd.mod11	cd.mod12	cd.mod13	cd.mod14	cd.mod15	cd.mod16	cd.mod17	cd.mod18	cd.mod19

cd.mod		3.55E-04	4.00E-04	1.39E-03	7.78E-16	0.0002132	0.0002132	0.0002132	0.0002132	0.098	0.001697	0.0006463	0.000264	0.0003191	0.0008435
cd.mod2	3.55E-04		0.1533	0.2329	< 2.2E-16	0.06107	0.06107	0.06107	0.06107	<2.2E-16	0.4336	0.05048	0.09124	0.06718	9.30E-02
cd.mod3	4.00E-04	0.1533		3.09E-01	< 2.2E-16	0.05947	0.05947	0.05947	0.05947	0.0001642	0.7047	0.0621	0.007755	0.06664	1.16E-01
cd.mod4	1.39E-03	0.2329	3.09E-01		< 2.2E-16	0.7416	0.7416	0.7416	0.7416	0.001921	0.1288	0.06532	0.8547	0.01879	8.82E-02
cd.mod9	7.78E-16	< 2.2E-16	< 2.2E-16	< 2.2E-16		< 2.2E-16	< 2.2E-16	< 2.2E-16	< 2.2E-16	< 2.2E-16	< 2.2E-16	< 2.2E-16	< 2.2E-16	< 2.2E-16	< 2.2E-16
cd.mod10	0.0002132	0.06107	0.05947	0.7416	< 2.2E-16		1	1	1	0.0001396	9.13E-02	0.2436	0.2114	0.1486	0.2291
cd.mod11	0.0002132	0.06107	0.05947	0.7416	< 2.2E-16	1		1	1	0.0001396	9.13E-02	0.2436	0.2114	0.1486	0.2291
cd.mod12	0.0002132	0.06107	0.05947	0.7416	< 2.2E-16	1	1		1	0.0001396	9.13E-02	0.2436	0.2114	0.1486	0.2291
cd.mod13	0.0002132	0.06107	0.05947	0.7416	< 2.2E-16	1	1	1		0.0001396	9.13E-02	0.2436	0.2114	0.1486	0.2291
cd.mod14	0.098	<2.2E-16	0.0001642	0.001921	< 2.2E-16	0.0001396	0.0001396	0.0001396	0.0001396		0.001903	0.0009463	0.0002353	0.0004185	0.001349
cd.mod15	0.001697	0.4336	0.7047	0.1288	< 2.2E-16	9.13E-02	9.13E-02	9.13E-02	9.13E-02	0.001903		0.0486	< 2.2E-16	0.02208	0.05779
cd.mod16	0.0006463	0.05048	0.0621	0.06532	< 2.2E-16	0.2436	0.2436	0.2436	0.2436	0.0009463	0.0486		0.273	0.7998	2.68E-01
cd.mod17	0.000264	0.09124	0.007755	0.8547	< 2.2E-16	0.2114	0.2114	0.2114	0.2114	0.0002353	< 2.2E-16	0.273		0.1572	0.2547
cd.mod18	0.0003191	0.06718	0.06664	0.01879	< 2.2E-16	0.1486	0.1486	0.1486	0.1486	0.0004185	0.02208	0.7998	0.1572		4.62E-01
cd.mod19	0.0008435	9.30E-02	1.16E-01	8.82E-02	< 2.2E-16	0.2291	0.2291	0.2291	0.2291	0.001349	0.05779	2.68E-01	0.2547	4.62E-01	
Ba															
	ba.mod	ba.mod2	ba.mod3	ba.mod4	ba.mod9	ba.mod10	ba.mod11	ba.mod12	ba.mod13	ba.mod14	ba.mod15	ba.mod16	ba.mod17	ba.mod18	ba.mod19
ba.m		-	<	<	-	-	-	-	-	3.60E	1.61E	<	<	<	

od			2.2E-16	2.2E-16						-04	-12	2.2E-16	2.2E-16	2.2E-16	
ba.mod2	-		-	-	< 2.2E-16	0.8452	0.8452	0.8452	0.8452	-	-	-	-	-	
ba.mod3	< 2.2E-16	-		3.98E-06	-	-	-	-	-	< 2.2E-16	< 2.2E-16	1.68E-05	0.007526	0.004476	
ba.mod4	< 2.2E-16	-	3.98E-06		-	-	-	-	-	< 2.2E-16	< 2.2E-16	0.6307	4.18E-05	2.03E-05	
ba.mod9	-	< 2.2E-16	-	-		< 2.2E-16	< 2.2E-16	< 2.2E-16	< 2.2E-16	-	-	-	-	-	
ba.mod10	-	0.8452	-	-	< 2.2E-16		1	1	1	-	-	-	-	-	
ba.mod11	-	0.8452	-	-	< 2.2E-16	1		1	1	-	-	-	-	-	
ba.mod12	-	0.8452	-	-	< 2.2E-16	1	1		1	-	-	-	-	-	
ba.mod13	-	0.8452	-	-	< 2.2E-16	1	1	1		-	-	-	-	-	
ba.mod14	3.60E-04	-	< 2.2E-16	< 2.2E-16	-	-	-	-	-		1.53E-10	< 2.2E-16	< 2.2E-16	< 2.2E-16	
ba.mod15	1.61E-12	-	< 2.2E-16	< 2.2E-16	-	-	-	-	-	1.53E-10		< 2.2E-16	< 2.2E-16	< 2.2E-16	
ba.mod16	< 2.2E-16	-	1.68E-05	0.6307	-	-	-	-	-	< 2.2E-16	< 2.2E-16		0.0004857	0.002554	
ba.mod17	< 2.2E-16	-	0.007526	4.18E-05	-	-	-	-	-	< 2.2E-16	< 2.2E-16	0.0001941		0.0001941	
ba.mod18	< 2.2E-16	-	0.004476	2.03E-05	-	-	-	-	-	< 2.2E-16	< 2.2E-16	0.0002624	0.0001941		
ba.mod19															

U

	u.mod	u.mod2	u.mod3	u.mod4	u.mod9	u.mod10	u.mod11	u.mod12	u.mod13	u.mod14	u.mod15	u.mod16	u.mod17	u.mod18	u.mod19
u.mod															

u.mod2			3.09E-03	0.0005158	2.20E-16	0.0008784	0.0008784	0.0008784	0.0008784	2.20E-16	5.82E-12	0.0001017	0.0007368	0.0006387	
u.mod3		3.09E-03		9.98E-03	2.20E-16	0.02107	0.02107	0.02107	0.02107	2.20E-16	1.21E-14	0.001773	0.01692	0.01139	
u.mod4		0.0005158	9.98E-03		2.20E-16	0.04686	0.04686	0.04686	0.04686	2.20E-16	2.20E-16	0.02021	0.07722	0.2223	
u.mod9		2.20E-16	2.20E-16	2.20E-16		2.20E-16	2.20E-16	2.20E-16	2.20E-16	0.001244	2.20E-16	2.20E-16	2.20E-16	2.20E-16	
u.mod10		0.0008784	0.02107	0.04686	2.20E-16		1	1	1	2.20E-16	2.20E-16	0.007556	0.0921	0.0508	
u.mod11		0.0008784	0.02107	0.04686	2.20E-16	1		1	1	2.20E-16	2.20E-16	0.007556	0.0921	0.0508	
u.mod12		0.0008784	0.02107	0.04686	2.20E-16	1	1		1	2.20E-16	2.20E-16	0.007556	0.0921	0.0508	
u.mod13		0.0008784	0.02107	0.04686	2.20E-16	1	1	1		2.20E-16	2.20E-16	0.007556	0.0921	0.0508	
u.mod14		2.20E-16	2.20E-16	2.20E-16	0.001244	2.20E-16	2.20E-16	2.20E-16	2.20E-16		2.20E-16	2.20E-16	2.20E-16	2.20E-16	
u.mod15		5.82E-12	1.21E-14	2.20E-16	2.20E-16	2.20E-16	2.20E-16	2.20E-16	2.20E-16	2.20E-16		2.20E-16	2.20E-16	2.20E-16	
u.mod16		0.0001017	0.001773	0.02021	2.20E-16	0.007556	0.007556	0.007556	0.007556	2.20E-16	2.20E-16		0.01165	0.01198	
u.mod17		0.0007368	0.01692	0.07722	2.20E-16	0.0921	0.0921	0.0921	0.0921	2.20E-16	2.20E-16	0.01165		0.08535	
u.mod18		0.0006387	0.01139	0.2223	2.20E-16	0.0508	0.0508	0.0508	0.0508	2.20E-16	2.20E-16	0.01198	0.08535		
u.mod19															

Na

	na.mod	na.mod2	na.mod3	na.mod4	na.mod9	na.mod10	na.mod11	na.mod12	na.mod13	na.mod14	na.mod15	na.mod16	na.mod17	na.mod18	na.mod19
na.mod															
na.mod2			-	-	4.29E-06	0.003533	0.003533	0.003533	0.003533	-	-	-	-	-	
na.mod3		-		6.92E-04	-	-	-	-	-	2.29E-07	2.00E-04	0.00262	5.37E-04	0.003623	
na.mod4		-	6.92E-04		-	-	-	-	-	8.87E-09	1.30E-08	0.6522	0.1212	0.1836	
na.mod9		4.29E-06	-	-		4.26E-07	4.26E-07	4.26E-07	4.26E-07	-	-	-	-	-	
na.mod10		0.003533	-	-	4.26E-07		1	1	1	-	-	-	-	-	
na.mod11		0.003533	-	-	4.26E-07	1		1	1	-	-	-	-	-	

na.mod12		0.003533	-	-	4.26E-07	1	1		1	-	-	-	-	-	
na.mod13		0.003533	-	-	4.26E-07	1	1	1		-	-	-	-	-	
na.mod14		-	2.29E-07	8.87E-09	-	-	-	-	-		0.02098	5.82E-08	4.36E-09	6.49E-08	
na.mod15		-	2.00E-04	1.30E-08	-	-	-	-	-	0.02098		1.66E-07	< 2.23E-16	1.52E-07	
na.mod16		-	0.00262	0.6522	-	-	-	-	-	5.82E-08	1.66E-07		0.2796	0.1053	
na.mod17		-	5.37E-04	0.1212	-	-	-	-	-	4.36E-09	< 2.23E-16	0.2796		0.4838	
na.mod18		-	0.003623	0.1836	-	-	-	-	-	6.49E-08	1.52E-07	0.1053	0.4838		
na.mod19															

C. Diagnostic plots for final models

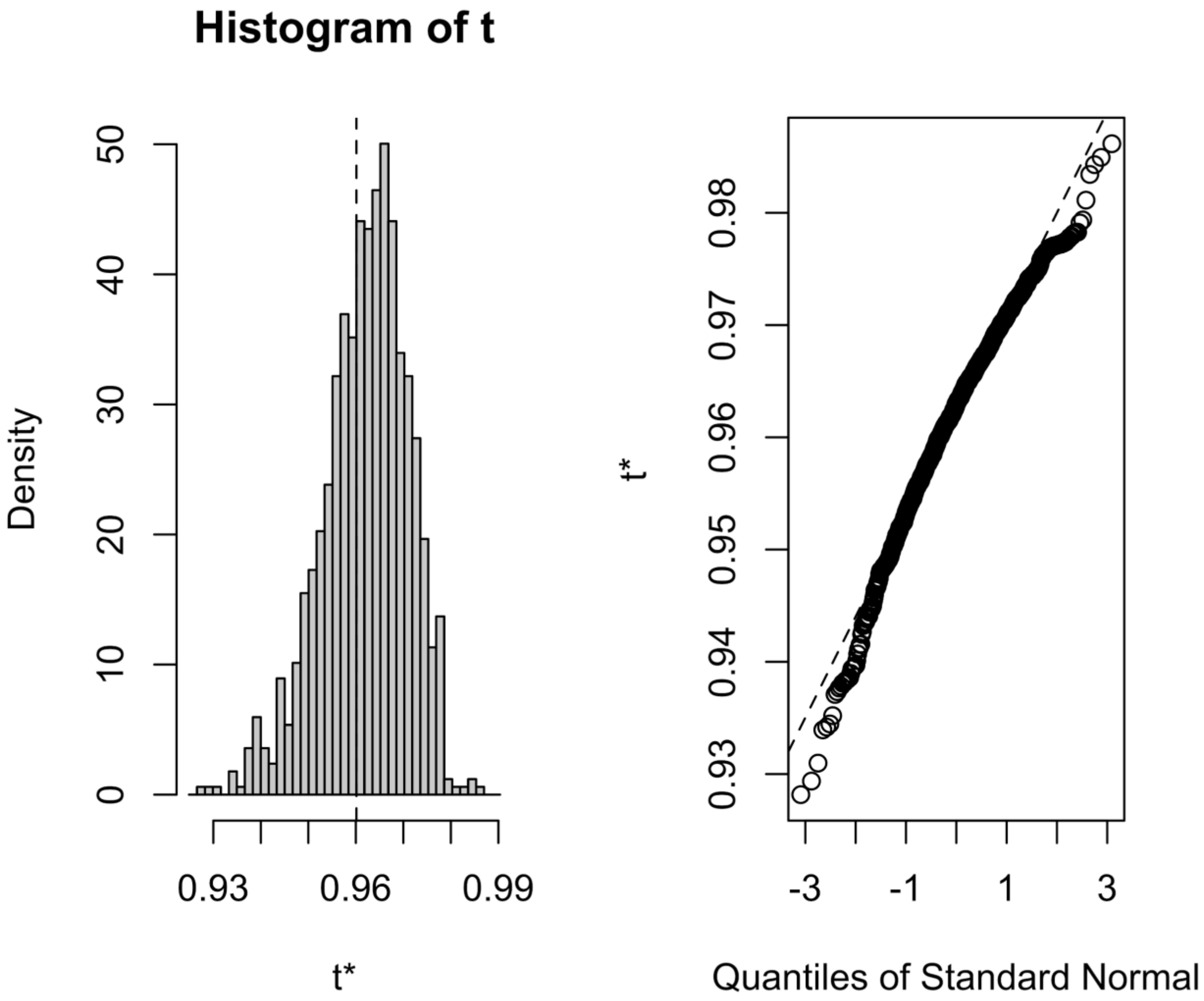

Figure S1. Diagnostic plots for Li.Ca ~ Mg.Ca * Phylum + Carbonate.Material. The left panel is a histogram of the residuals and the right panel is a Q-Q plot.

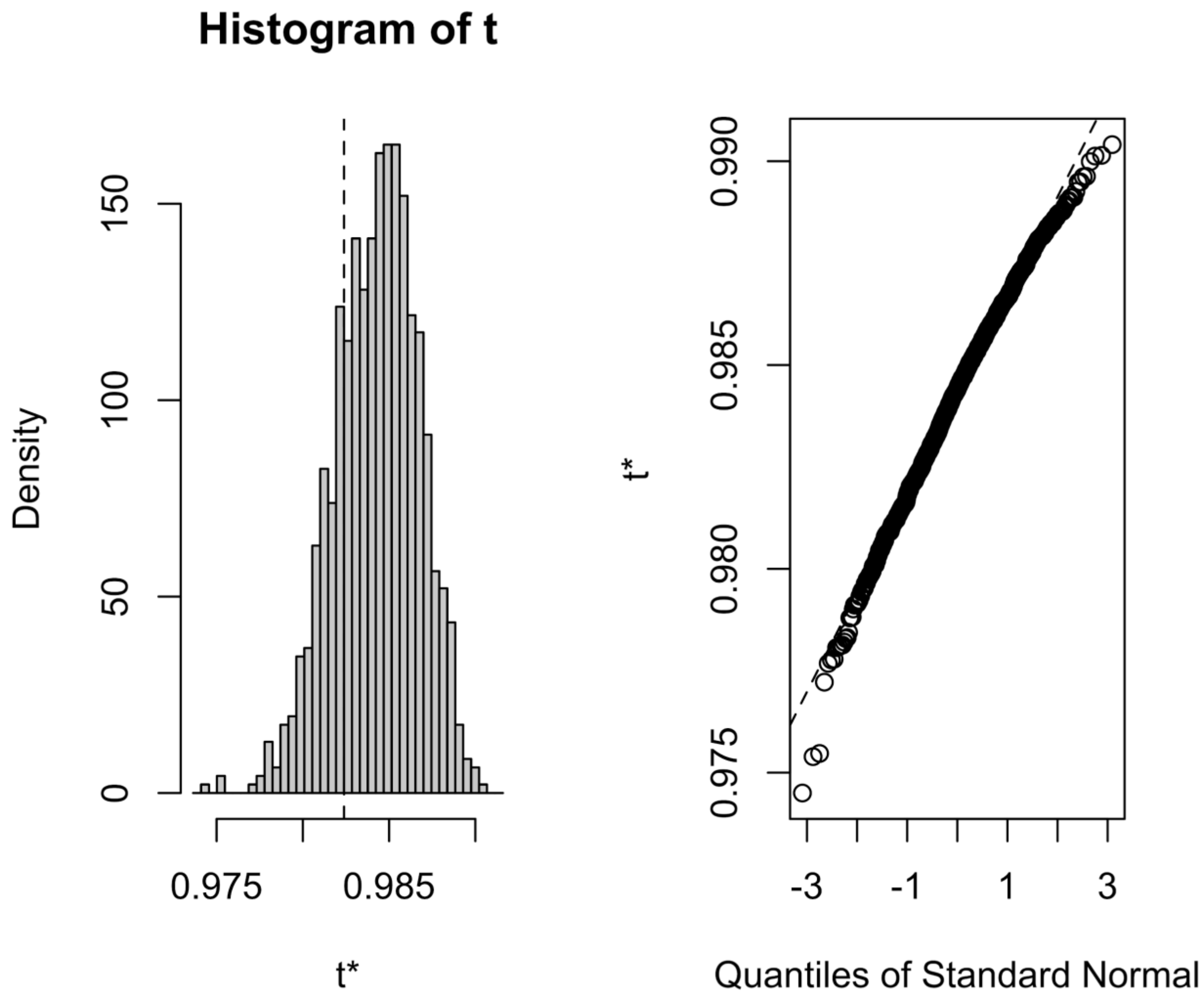

Figure S2. Diagnostic plots for B.Ca ~ Mg.Ca * Phylum. The left panel is a histogram of the residuals and the right panel is a Q-Q plot.

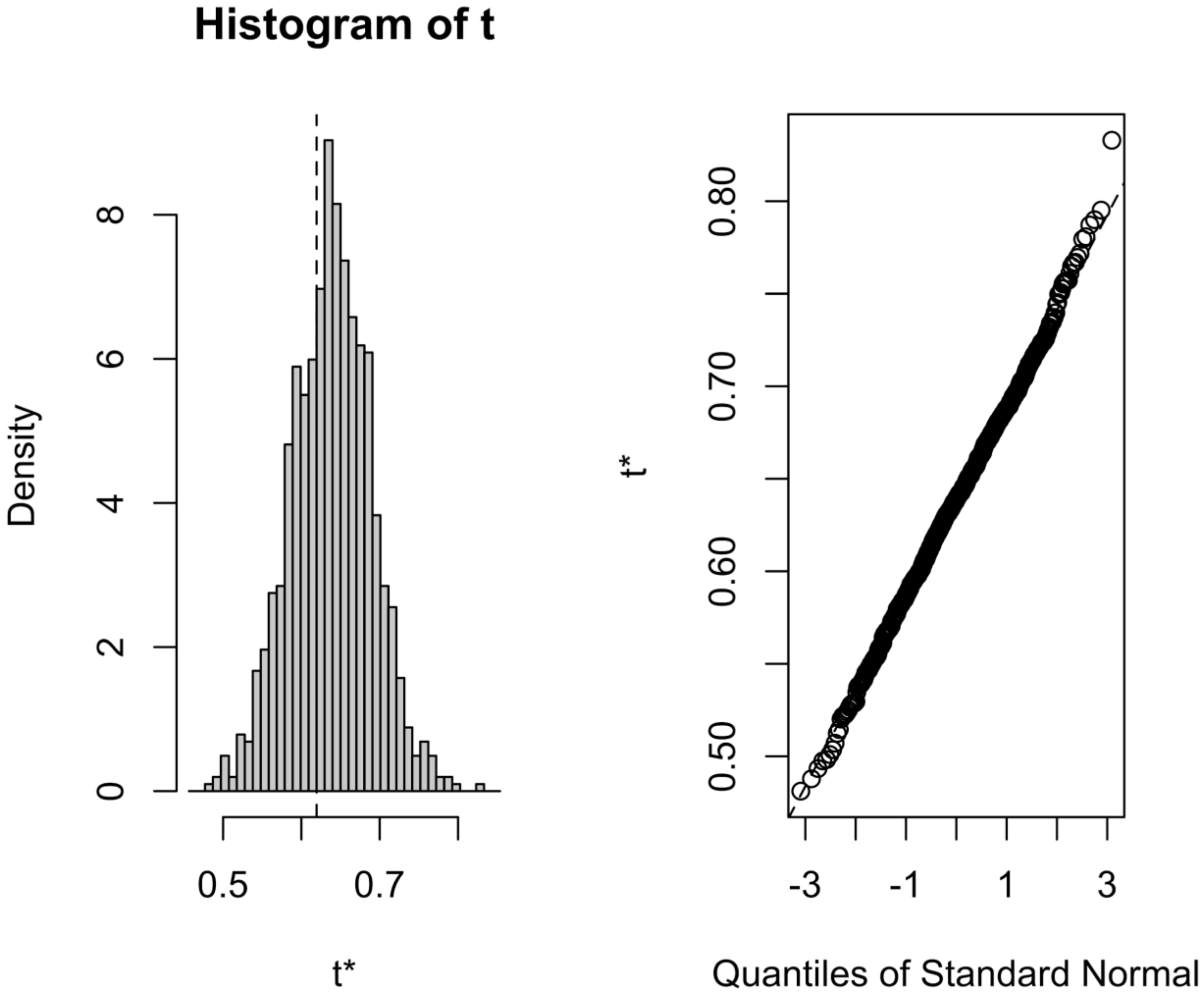

Figure S3. Diagnostic plots for Zn.Ca ~ Mg.Ca * Phylum. The left panel is a histogram of the residuals and the right panel is a Q-Q plot.

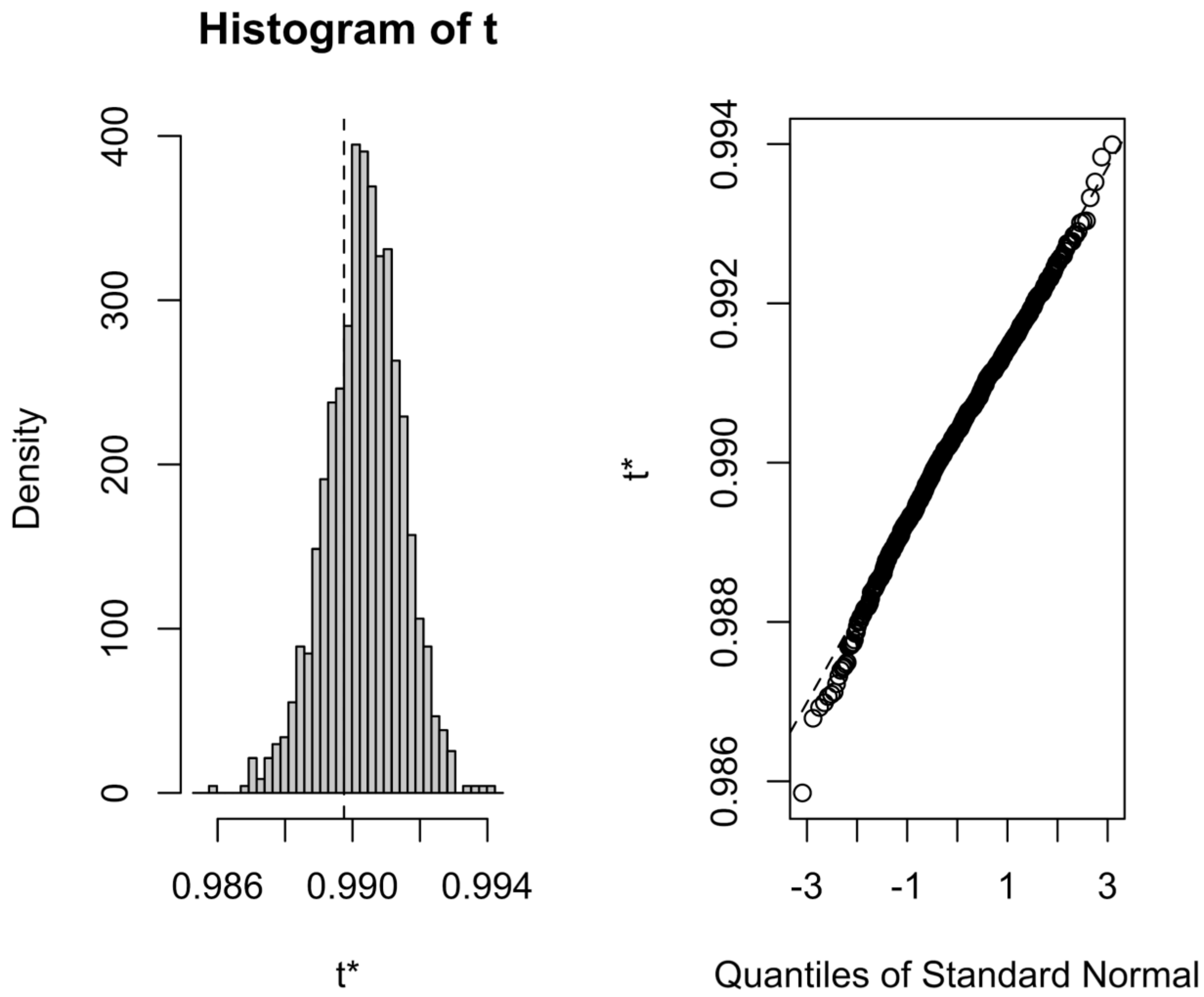

Figure S4. Diagnostic plots for Sr.Ca ~ Mg.Ca * Phylum + Carbonate.Material. The left panel is a histogram of the residuals and the right panel is a Q-Q plot.

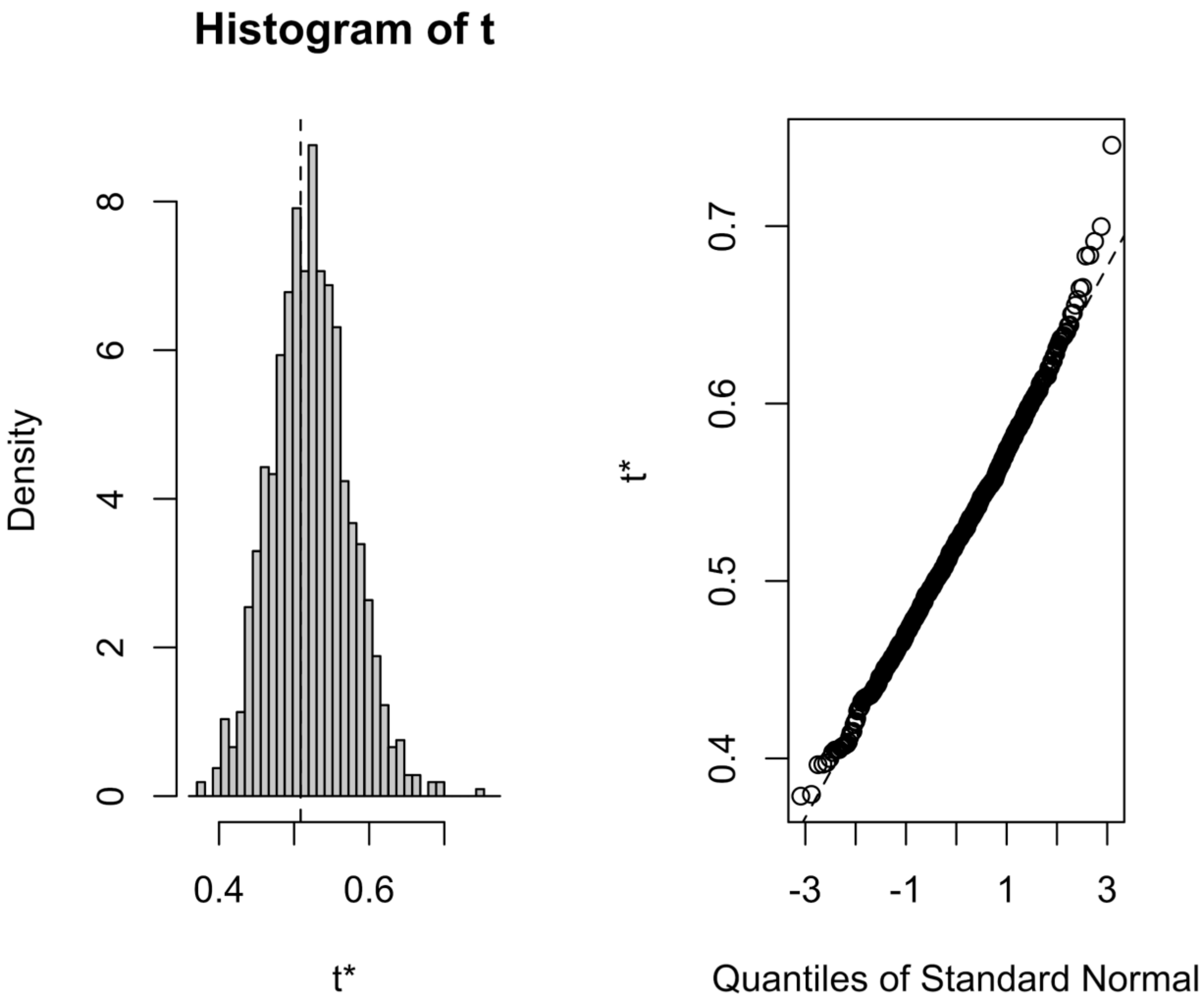

Figure S5. Diagnostic plots for Cd.Ca ~ Carbonate.Material. The left panel is a histogram of the residuals and the right panel is a Q-Q plot.

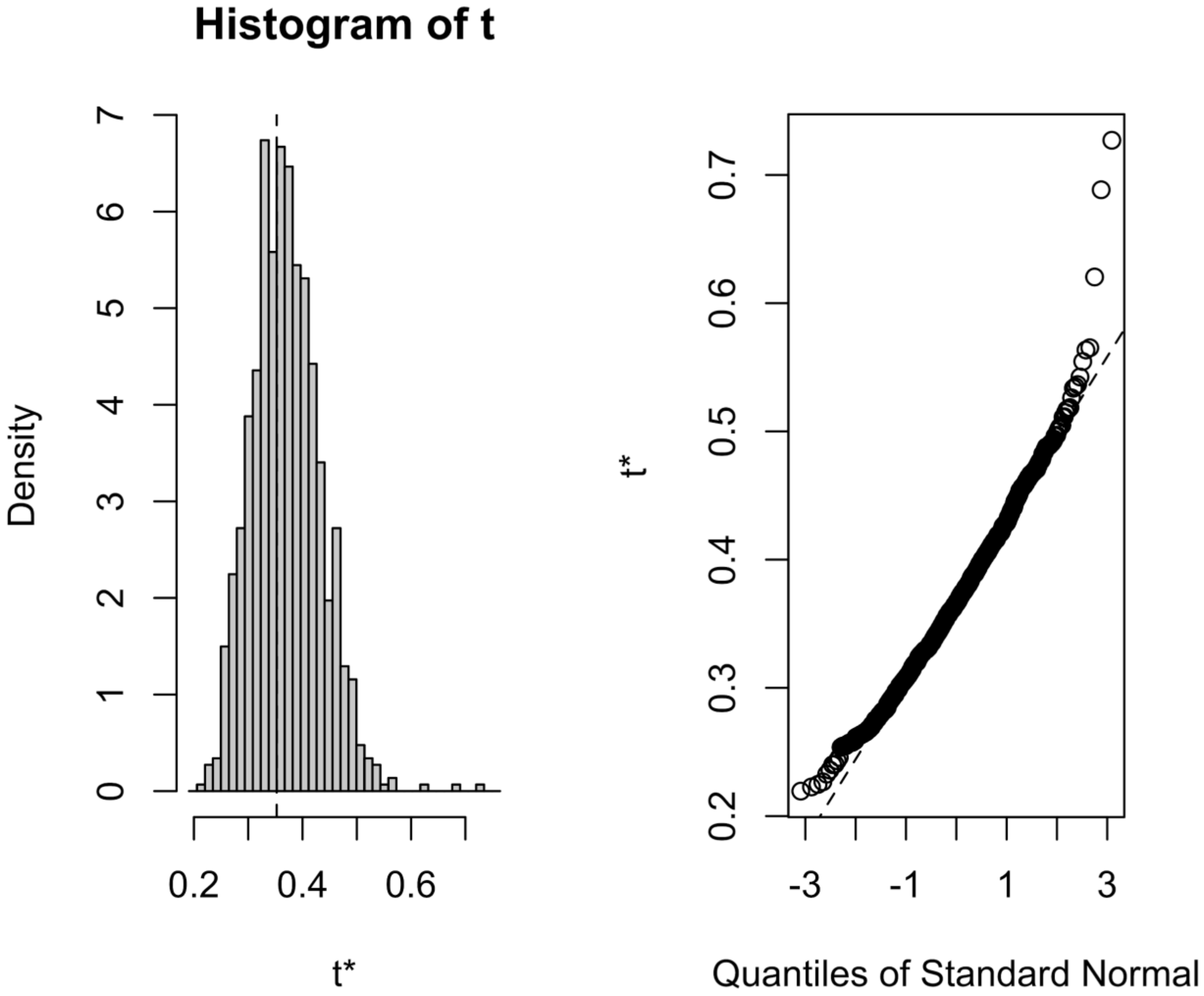

Figure S6. Diagnostic plots for Ba.Ca ~ Mg.Ca * Phylum. The left panel is a histogram of the residuals and the right panel is a Q-Q plot.

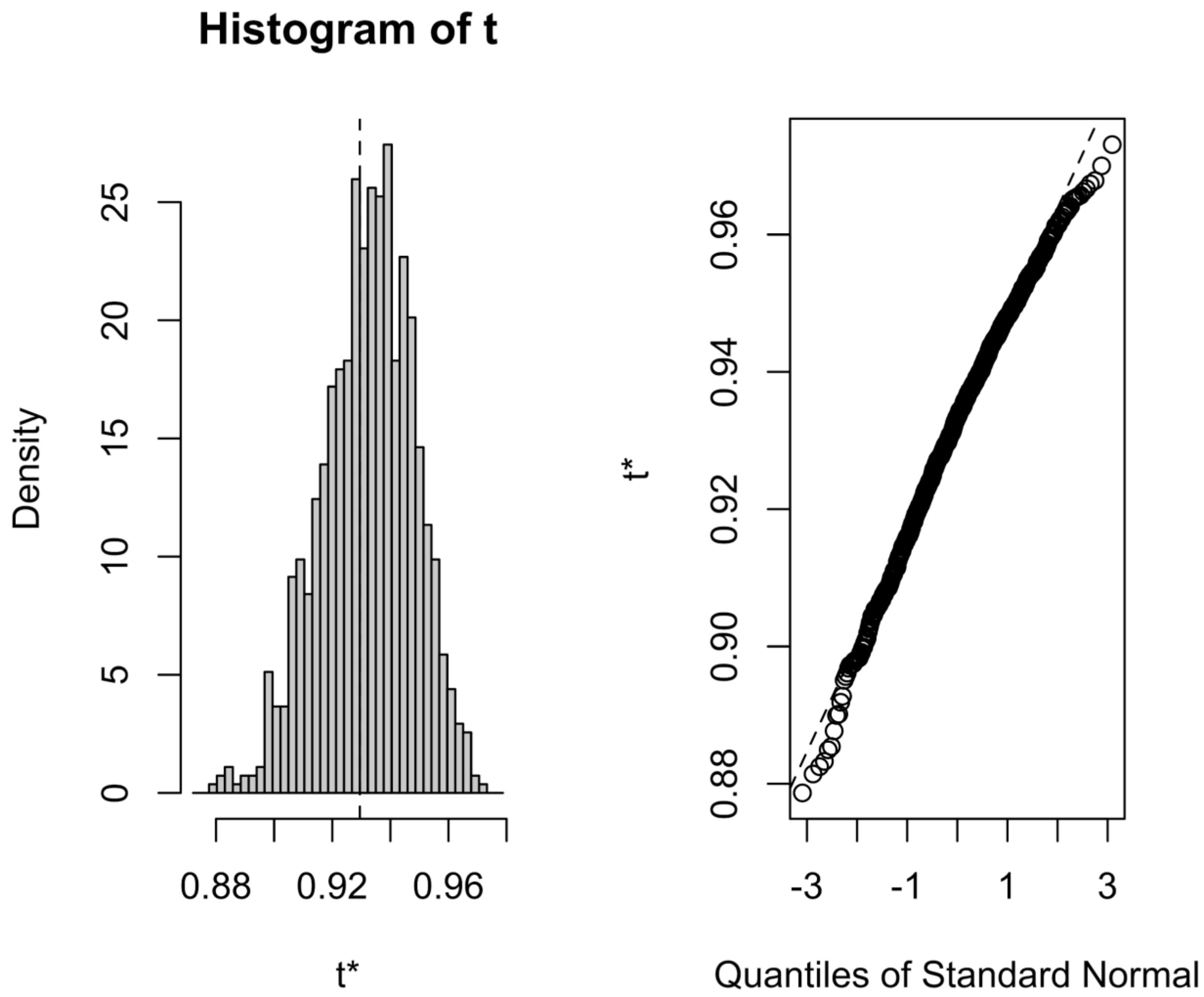

Figure S7. Diagnostic plots for U.Ca ~ Mg.Ca * Phylum + Carbonate.Material. The left panel is a histogram of the residuals and the right panel is a Q-Q plot.

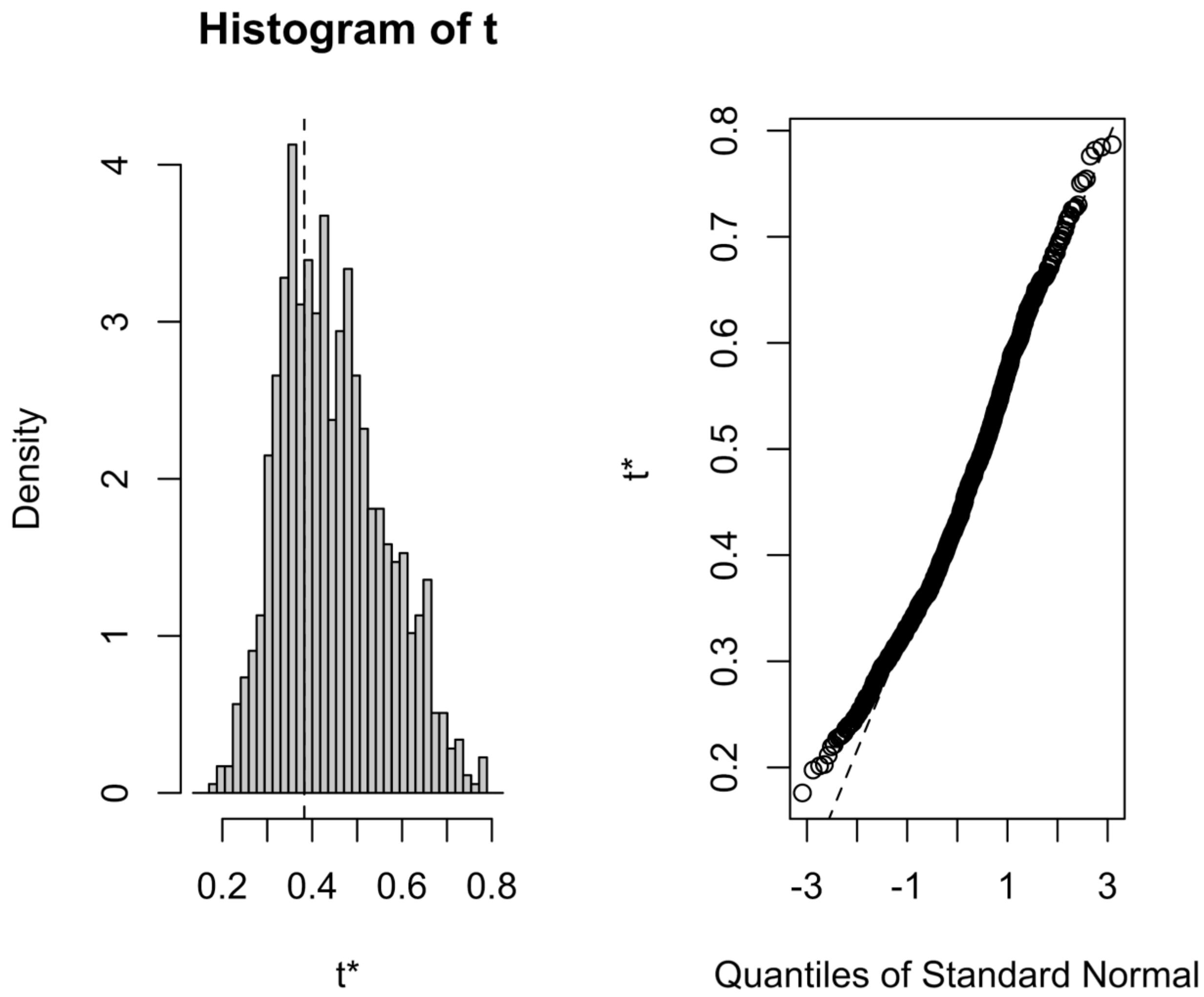

Figure S8. Diagnostic plots for the li.mod (Na.Ca ~ Mg.Ca * Phylum). The left panel is a histogram of the residuals and the right panel is a Q-Q plot.

D. Figures showing final model components

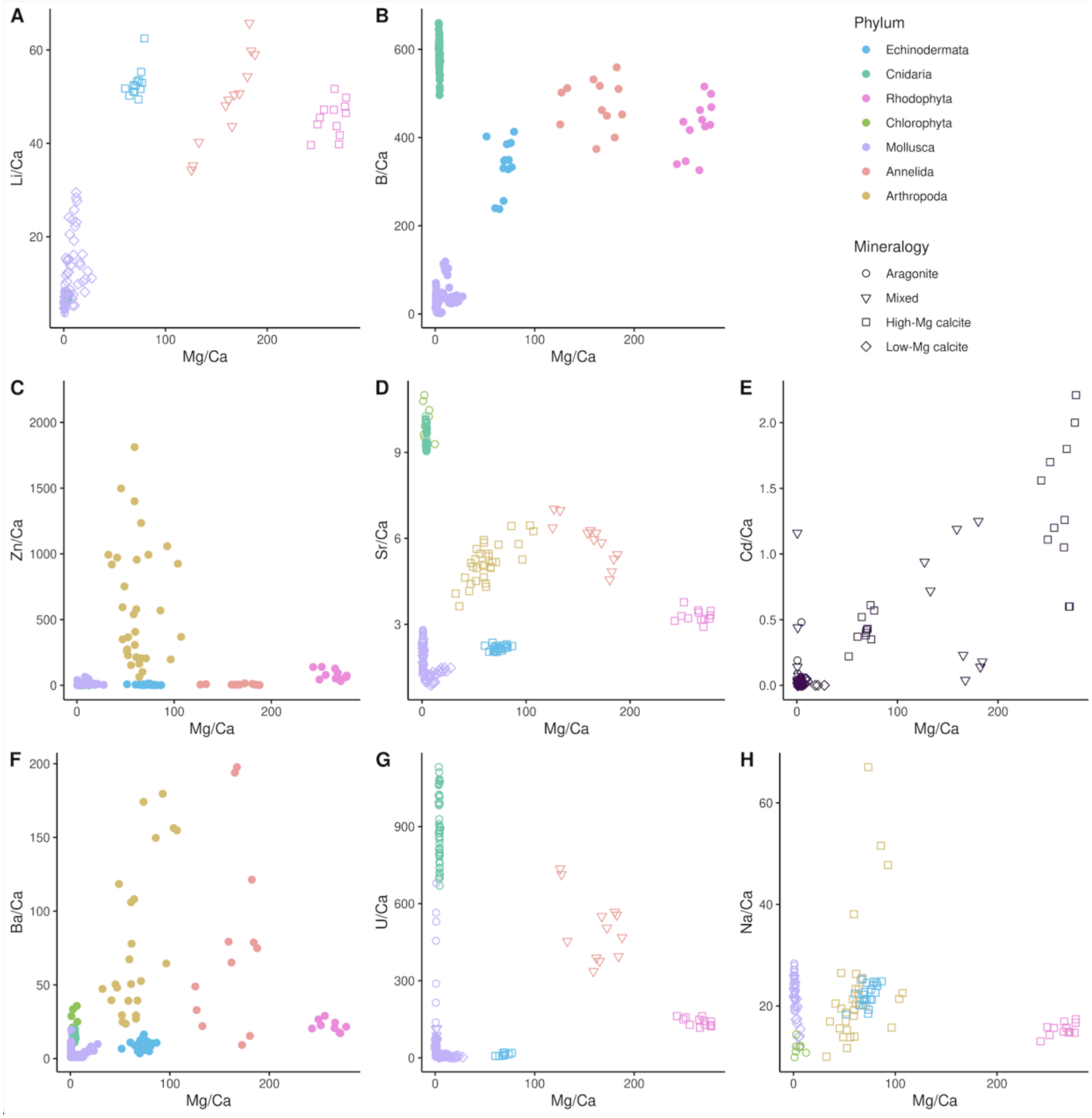

Figure S9. Array of scatterplots displaying the model components of the final GAMs (See Table 2 in the main text). These scatterplots are similar to Figure 2 in that they plot eight of the elemental ratios against measured Mg/Ca for each of the samples.

IV. Description of inorganic partition coefficient selection
Inorganic partition coefficients for aragonite and calcite were selected based on conditions akin to those used for the Ries *et al.* (2009) culturing experiments: filtered seawater, 25°C and pH conditions in the range of 7.2 - 8.5, respectively. If the criteria were not met, a value that satisfied at least one criterion was used, as the inorganic partition coefficients present in the literature can differ considerably. The tabulated values, mineral growth rates, experimental conditions, and references can be found in Table S5.

In aragonite, for Sr/Ca and U/Ca, values of 1.13 and 0.248 were chosen, respectively because they were obtained from a precipitation experiment using filtered seawater at a temperature of 25.5 °C and within our pH range (DeCarlo *et al.*, 2015). The experiments from Gabitov *et al.* (2011) used artificial seawater and were conducted at 25 °C, and thus were sufficient for determining our Mg/Ca partition coefficient in calcite as 4.72 x 10^{-4}. The experiments of Mavromatis *et al.* (2018) were conducted at 25 °C using electrolyte solutions (250 mM NaCl and 250 mM $MgCl_2$), providing us with a partition coefficient for Ba/Ca of 0.22. However, these experiments were conducted at a pH below our target range (6.3), so an additional value of 2.11 was selected from an experiment at an equivalent temperature and used similar apparatus, but a higher pH, up to 9.65 (Gaetani and Cohen, 2006). For B/Ca in aragonite, two inorganic partition coefficient values were chosen (defined here as B/Ca/[B(OH)4-]/[CO32-]): 2.48 and 0.02 (Holcomb, 2016; Mavromatis *et al.*, 2015). The first value was because it was obtained from an experiment that used seawater, with a salinity range of 32 - 37 psu, a temperature of 25 °C, and an average pH of 8.05. For Li/Ca in aragonite, we chose an inorganic partition coefficient value of 3.21 x 10^{-2} because it was obtained from an experiment using filtered seawater at 25 °C (Gabitov *et al.*, 2011). For Zn/Ca in aragonite, we chose an inorganic partition coefficient value of 5.7 because it was obtained from an experiment using stock solutions at 25 °C (Crocket *et al.*, 1966).

In calcite, for Sr/Ca, Mg/Ca, Ba/Ca, B/Ca, inorganic partition coefficient values were 2.60 x 10^{-1}, 2.66 x 10^{-2}, 9.37 x 10^{-1}, and 4.14 x 10^{-3}, respectively, because they were obtained from an experiment using artificial seawater (Gabitiov *et al.*, 2019). Caveats with these values are that the salinity of the artificial seawater is 25 psu, the pH was higher than 8.5, and the experiment was conducted at room temperature. Since these experiments were conducted outside our specified conditions, additional values were selected. For the Ba/Ca partition coefficient values of 4.0 x 10^{-3} - 9.0 x 10^{-3} from Mavromatis *et al.* (2018) were chosen. Likewise, for B/Ca the value from Mavromatis *et al*. (2015) of 1.4 x 10^{-6}, derived from experiments using the specified conditions described previously, was included. For Li/Ca in calcite, the inorganic partition coefficient values of 0.00014 (Füger *et al.*, 2019) was added to the Gabitov *et al*. (2019) value, it was obtained from an experiment similar to the one conducted in Mavromatis *et al.* (2018), using an NaCl solution at 25 °C. For Cd/Ca in calcite, a range of inorganic partition coefficient of 20 - 100 were chosen, these values were reported from a crystallization experiment that used a silica gel column (Katsikopoulos *et al.*, 2008); however, the pH was low at 5.5 and the experimental temperature was not reported. For U/Ca in calcite, we chose an inorganic partition coefficient range of 0.02 - 0.06 because it was obtained from calcite growth experiments conducted within our pH range (Weremeichik *et al.*, 2017). For Zn/Ca in calcite, we chose an inorganic partition coefficient range of 9 - 158, with an average value of 54 because it was obtained from calcite growth experiments conducted at 25 °C (Mavromatis *et al.*, 2018). We calculated and obtained the value of 54 by excluding data retrieved from experiments outside of our experimental pH range.

Table S6. The inorganic partition coefficients (K_x) selected for each of the elemental ratios. Adjacent columns display relevant information, if provided, from the original studies these values were extracted from, including precipitation rate experimental conditions.

X/Ca	K_x	Precipitation Rate	Experimental Conditions	Source
**Aragonite**				
Sr/Ca	1.13	3.03×10^3 µmol/m^{2}hr*	Filtered seawater; 25.5 °C; pH = 7.2-8.5	DeCarlo *et al.*, 2015
Mg/Ca	4.72×10^{-4}	N/A	Filtered seawater; 25.5 °C	Gabitov *et al.*, 2011
Ba/Ca	0.22	$-9.0 \leq \log(r_p) \leq -7.8$ mol/m^2/s*	Artificial solution with electrolytes: 250 *m*M NaCl and 25 *m*M $MgCl_2$; 25 °C; pH = 6.3	Mavromatis *et al.*, 2018
	2.11	$R = (\exp(10.4 – 2038/T))(\Omega – 1)^{0.063T=17.0}$*	Filtered seawater; 25 °C; high pH	Gaetini and Cohen, 2006
B/Ca	2.0×10^{-2}	2.03×10^{-7} mol/m^2/s*	Estimations from DeCarlo *et al.*, 2015	Mavromatis *et al.*, 2015
	2.48	$1.18 \times 10^3 – 4.94 \times 10^4$ µmol/m^{2}hr*	Filtered seawater; 25 °C; Salinity = 32 – 37 psu; average pH = 8.05	Holcomb *et al.*, 2016
U/Ca	2.48×10^{-1}	3.03×10^3 µmol/m^{2}hr	Filtered seawater; 25.5 °C; pH = 7.2-8.5	DeCarlo *et al.*, 2015
Li/Ca	3.21×10^{-2}	N/A	Filtered seawater; 25 °C	Gabitov *et al.*, 2011
Zn/Ca	5.7	N/A	Stock solutions; 25 °C	Crocket *et al.*, 1966
**Calcite**				
Sr/Ca	2.59×10^{-1}	N/A	Artificial seawater; Room temperature; Salinity = 25 psu; pH > 8.5	Gabitov *et al.*, 2019
Mg/Ca	2.66×10^{-2}	N/A	Artificial seawater; Room temperature; Salinity = 25 psu; pH > 8.5	Gabitov *et al.*, 2019
Ba/Ca	$4.0 – 9.0 \times 10^{-3}$	Average $\log(r_p) = -7.73$ mol/m^2/s*	Artificial solution with electrolytes: 250 *m*M NaCl and 25 *m*M $MgCl_2$; 25 °C; pH = 6.3	Mavromatis *et al.*, 2018
	9.63×10^{-1}	N/A	Artificial seawater; Room temperature; Salinity = 25 psu; pH > 8.5	Gabitov *et al.*, 2019

B/Ca	1.40 x 10^{-6}	1.77 x 10^{-6} mol/m²/s*	25 °C; pH = 7.4 - 8.55	Mavromatis *et al.*, 2015
	4.14 x 10^{-3}	N/A	Artificial seawater; Room temperature; Salinity = 25 psu; pH > 8.5	Gabitov *et al.*, 2019
U/Ca	2.0 – 6.0 x 10^{-2}	0.01 - 0.14 nm/s*	Growth rate; Stock solutions; pH = 7.86 – 8.17	Weremeichik *et al.*, 2017
Li/Ca	1.4 x 10^{-4}	$-8.1 \leq \log(R) \leq -7.1$ mol/m²/s*	NaCl solution; 25 °C; Salinity = 18 psu	Fuger *et al.*, 2019
	4.9 x 10^{-3}	N/A	Artificial seawater; Room temperature; Salinity = 25; pH > 8.5	Gabitov *et al.*, 2019
Zn/Ca	5.40 x 10^{1}	$-8.1 \leq \log(R) \leq -7.6$ mol/m²/s*	Growth rate; 25 °C; pH = 7.2 – 8.5	Mavromatis *et al.*, 2018
Cd/Ca	1.85 x 10^{1}	$\log(\lambda) = -0.194\log(R\ \mathrm{mol/m^2/s}) + 1.46$***	Growth rate; Stock solutions; 25 °C; pH = 7.3 – 7.5	Lorens *et al.*, 1981
**Amorphous calcium carbonate**				
Li	1.68 x 10^{-3}	0.5 mL/min****	Artificial seawater; Room temperature; pH = 8.95 – 9.22	Evans *et al.*, 2020
B	3.5 x 10^{-2}	0.5 mL/min****	Artificial seawater; Room temperature; pH = 8.95 – 9.22	Evans *et al.*, 2020
Na	2.6 x 10^{-3}	0.5 mL/min****	Artificial seawater; Room temperature; pH = 8.95 – 9.22	Evans *et al.*, 2020
Mg	1.02 x 10^{-1}	0.5 mL/min****	Artificial seawater; Room temperature; pH = 8.95 – 9.22	Evans *et al.*, 2020
Sr	7.6 x 10^{-1}	0.5 mL/min****	Artificial seawater; Room temperature; pH = 8.95 – 9.22	Evans *et al.*, 2020
Ba	1.94 x 10^{1}	0.5 mL/min****	Artificial seawater; Room temperature; pH = 8.95 – 9.22	Evans *et al.*, 2020
U	1.2 x 10^{-1}	0.5 mL/min****	Artificial seawater; Room temperature; pH = 8.95 – 9.22	Evans *et al.*, 2020

*Growth rate
**Aragonite precipitation model calculations
***Distribution coefficient as a function of precipitation rate equation
****Titration rate

V. Scatterplot arrays of element-to-calcium ratios versus carbonate chemistry and other measured parameters

In this section, we present arrays of scatterplots for each of the organisms displaying all of the element-to-calcium ratio measurements plotted against carbonate chemistry and other measured parameters, including net calcification rates measured from Ries *et al.* (2009), seawater carbonate ion concentration, seawater pH, and boron-derived calcifying fluid pH from Liu *et al.* (2020). For each set of organismal data, two arrays are presented: 1) using linear regressions to fit the data and 2) using quadratic regressions to fit the data. Scatterplots with significant ($p<0.05$) relationships are highlighted with a red box. When relationships were significant, Akaike Information Criterion (AIC) values were compared to determine whether a linear or quadratic regression fit the data better. The summary of results of AIC comparisons can be found in the main text in Tables 2 - 4.

American Lobster – Linear Models

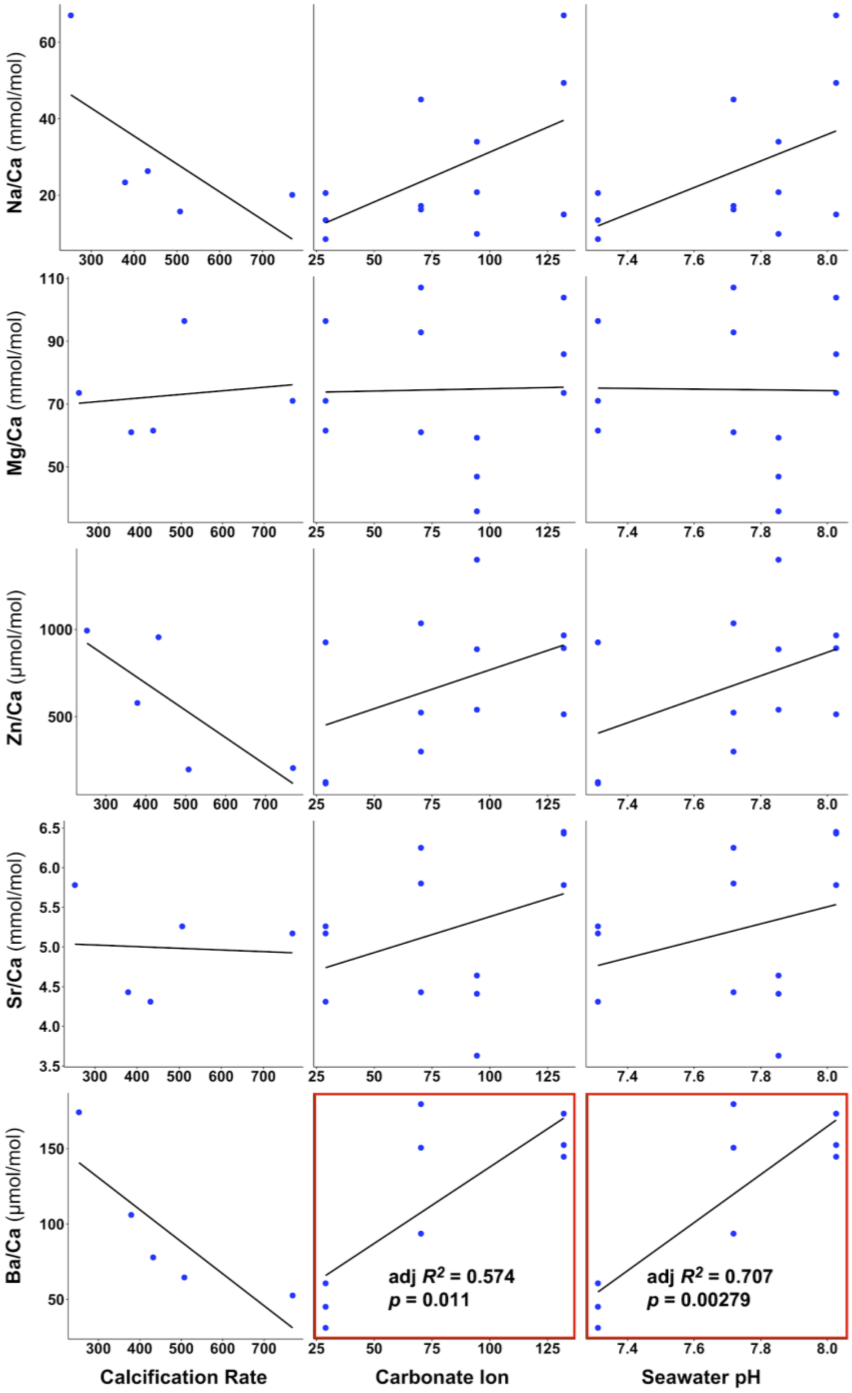

Figure S10. Array of scatterplots fitted with linear regressions displaying trace element-to-calcium ratios of American lobster samples plotted against carbonate chemistry and other measured parameters. Bottom labels and labels going down the left side of the array signify x-axis parameters and y-axis parameters, respectively, for a given plot. Scatterplots with regressions that possess a p-value < 0.05 are outlined in red. Significant relationships include Ba/Ca against seawater carbonate ion concentration and seawater pH.

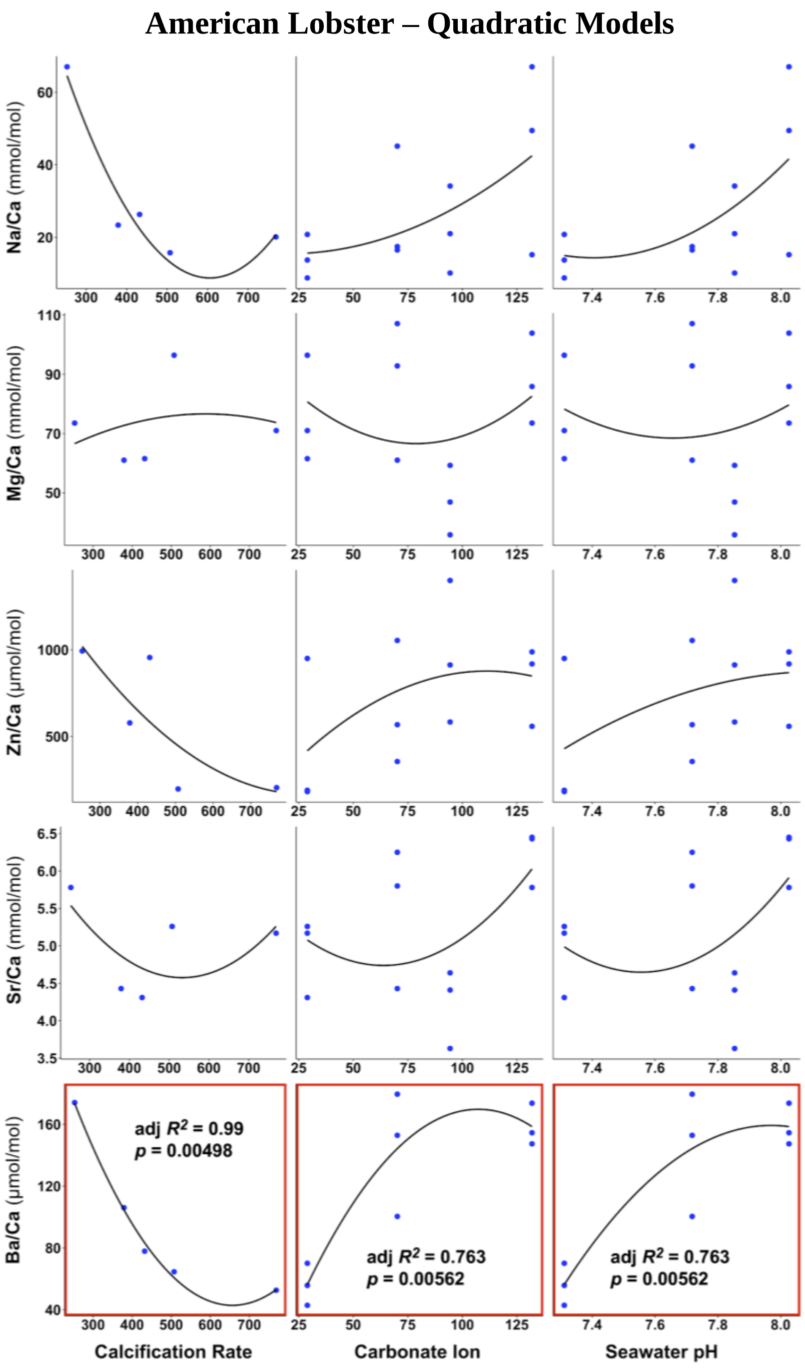

Figure S11. Array of scatterplots fitted with quadratic regressions displaying trace element-to-calcium ratios of American lobster samples plotted against carbonate chemistry and other measured parameters. Bottom labels and labels going down the left side of the array signify x-axis parameters and y-axis parameters, respectively, for a given plot. Scatterplots with regressions that possess a p-value < 0.05 are outlined in red. Significant relationships include Ba/Ca against net calcification rate, seawater carbonate ion concentration, and seawater pH. Significant relationships absent from the linear regression analysis are Ba/Ca against the net calcification rate.

American Oyster – Linear Models

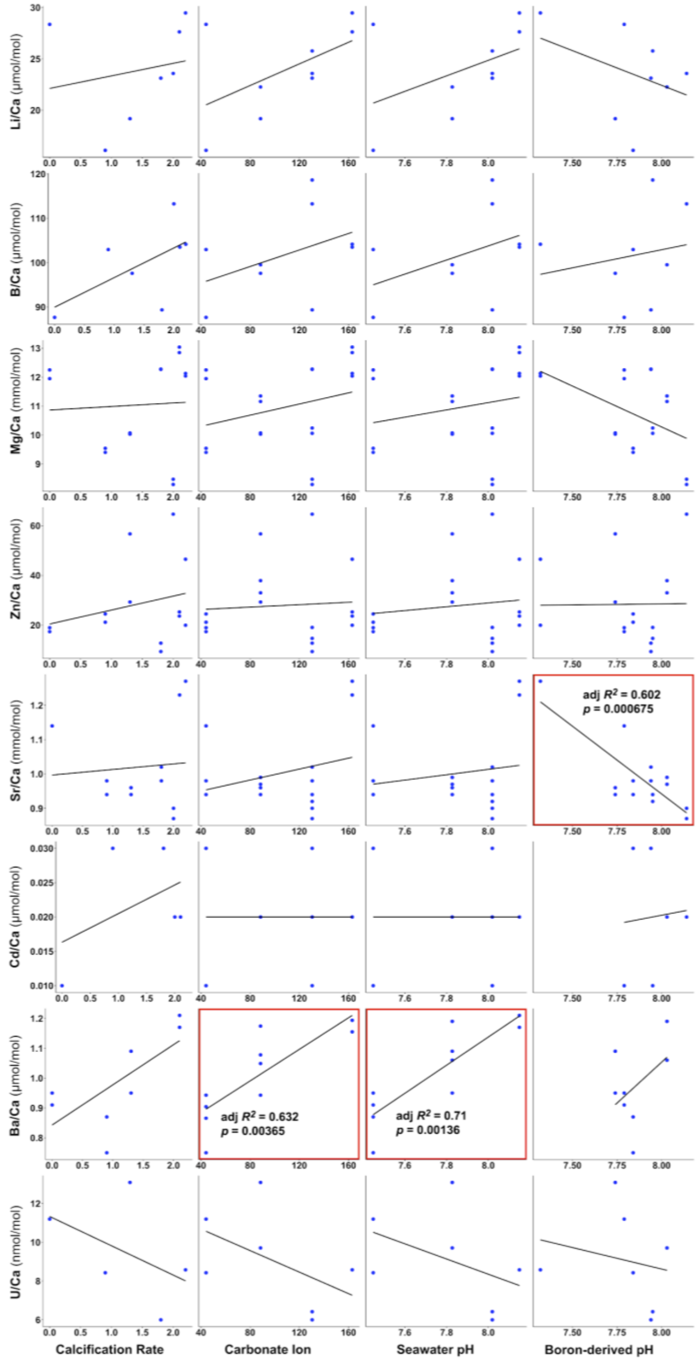

Figure S12. Array of scatterplots fitted with linear regressions displaying trace element-to-calcium ratios of the American oyster samples plotted against carbonate chemistry and other measured parameters. Bottom labels and labels going down the left side of the array signify x-axis parameters and y-axis parameters, respectively, for a given plot. Scatterplots with regressions that possess a p-value < 0.05 are outlined in red. Significant relationships include Sr/Ca against the boron-derived calcifying fluid pH and Ba/Ca against the seawater carbonate ion concentration and seawater pH.

American Oyster – Quadratic Models

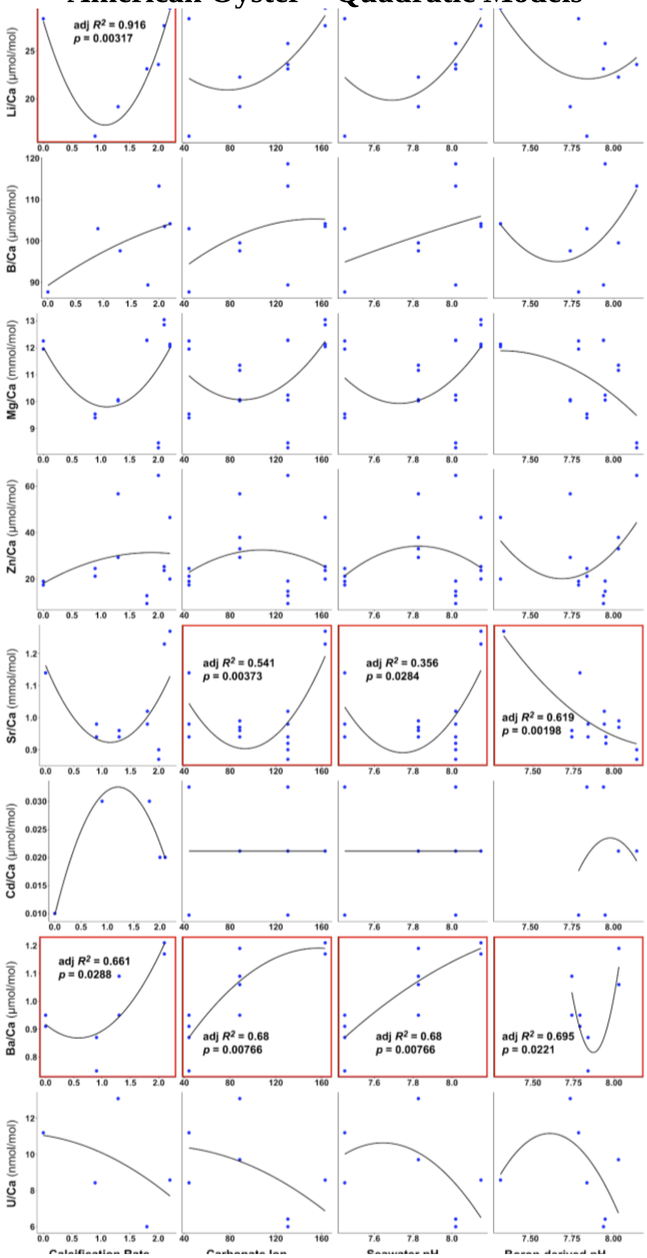

Figure S13. Array of scatterplots fitted with quadratic regressions displaying trace element-to-calcium ratios of the American oyster samples plotted against carbonate chemistry and other measured parameters. Bottom labels and labels going down the left side of the array signify x-axis parameters and y-axis parameters, respectively, for a given plot. Scatterplots with regressions that possess a p-value < 0.05 are outlined in red. Significant relationships include Li/Ca and Ba/Ca against net calcification rate and Sr/Ca and Ba/Ca against seawater carbonate ion concentration, seawater pH, and boron-derived calcifying fluid pH. Significant relationships absent from the linear regression analysis are Li/Ca and Ba/Ca again net calcification rate; Sr/Ca against seawater carbonate ion concentration and seawater pH; and Ba/Ca against boron-derived internal calcifying fluid pH.

Bay Scallop – Linear Models

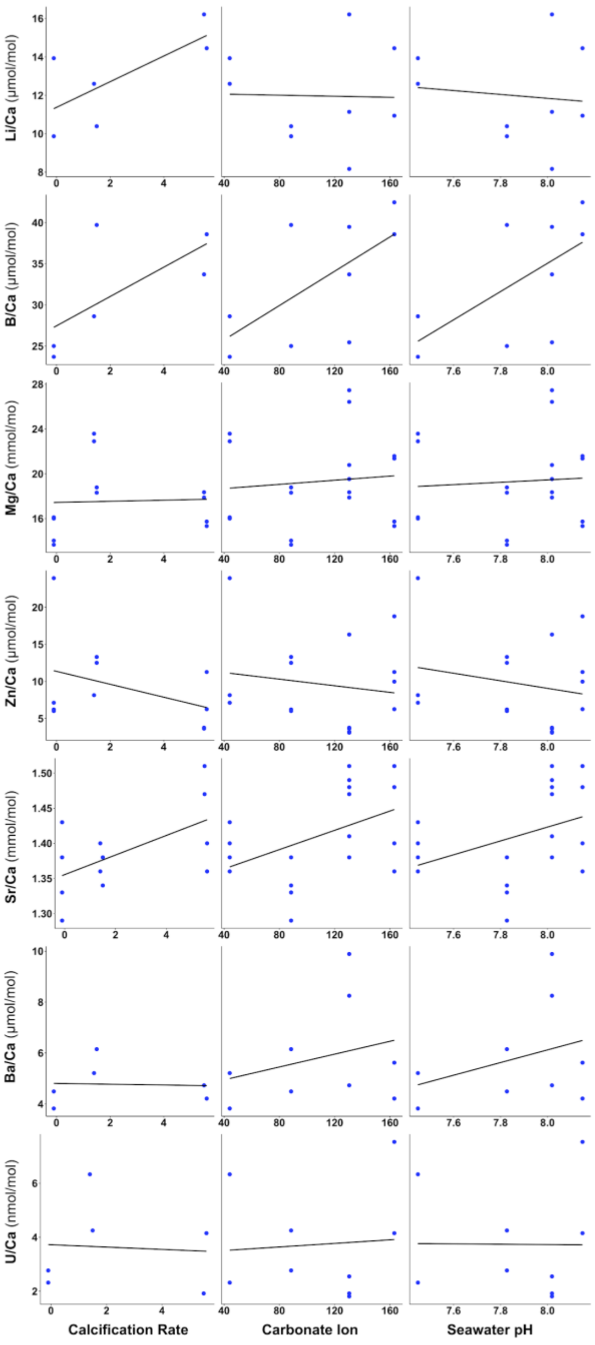

Figure S14. Array of scatterplots fitted with linear regressions displaying trace element-to-calcium ratios of the bay scallop samples plotted against carbonate chemistry and other measured parameters. Bottom labels and labels going down the left side of the array signify x-axis parameters and y-axis parameters, respectively, for a given plot. Scatterplots with regressions that possess a p-value < 0.05 are outlined in red. No significant relationships are observed, possibly due to low sample replication.

Bay Scallop – Quadratic Models

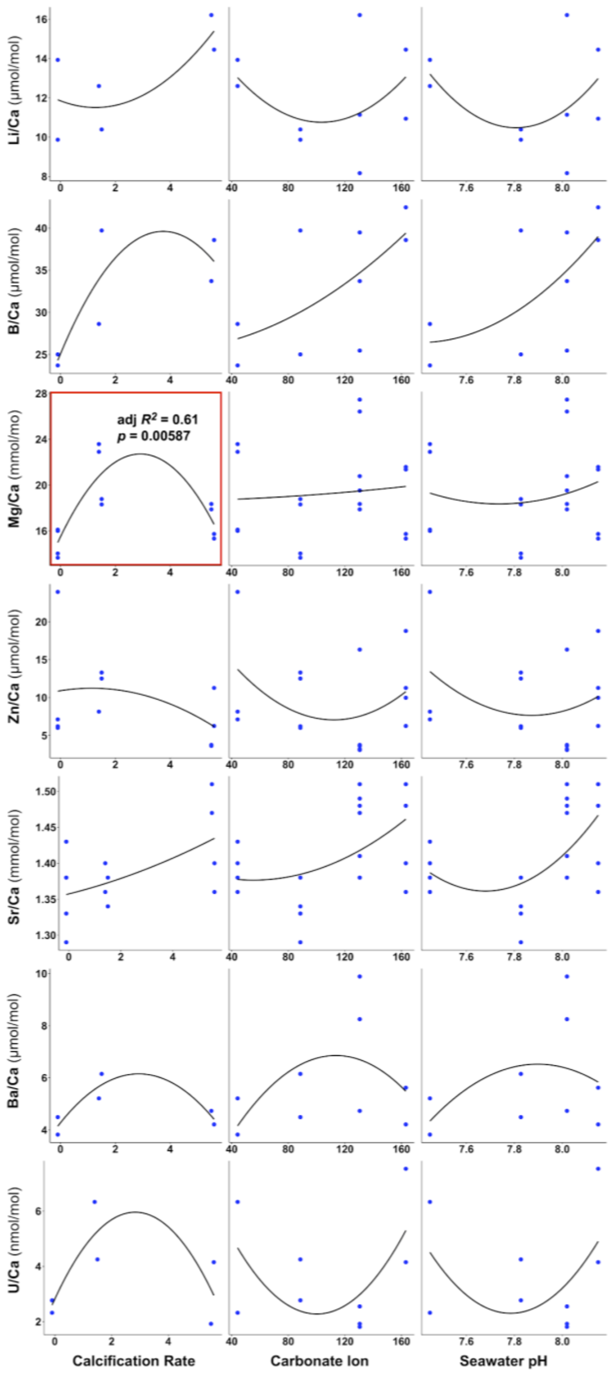

Figure S15. Array of scatterplots fitted with quadratic regressions displaying trace element-to-calcium ratios of the bay scallop samples plotted against carbonate chemistry and other measured parameters. Bottom labels and labels going down the left side of the array signify x-axis parameters and y-axis parameters, respectively, for a given plot. Scatterplots with regressions that possess a p-value < 0.05 are outlined in red. The significant relationship observed is Mg/Ca against net calcification rate, which is absent in the linear regression analysis.

Blue Crab – Linear Models

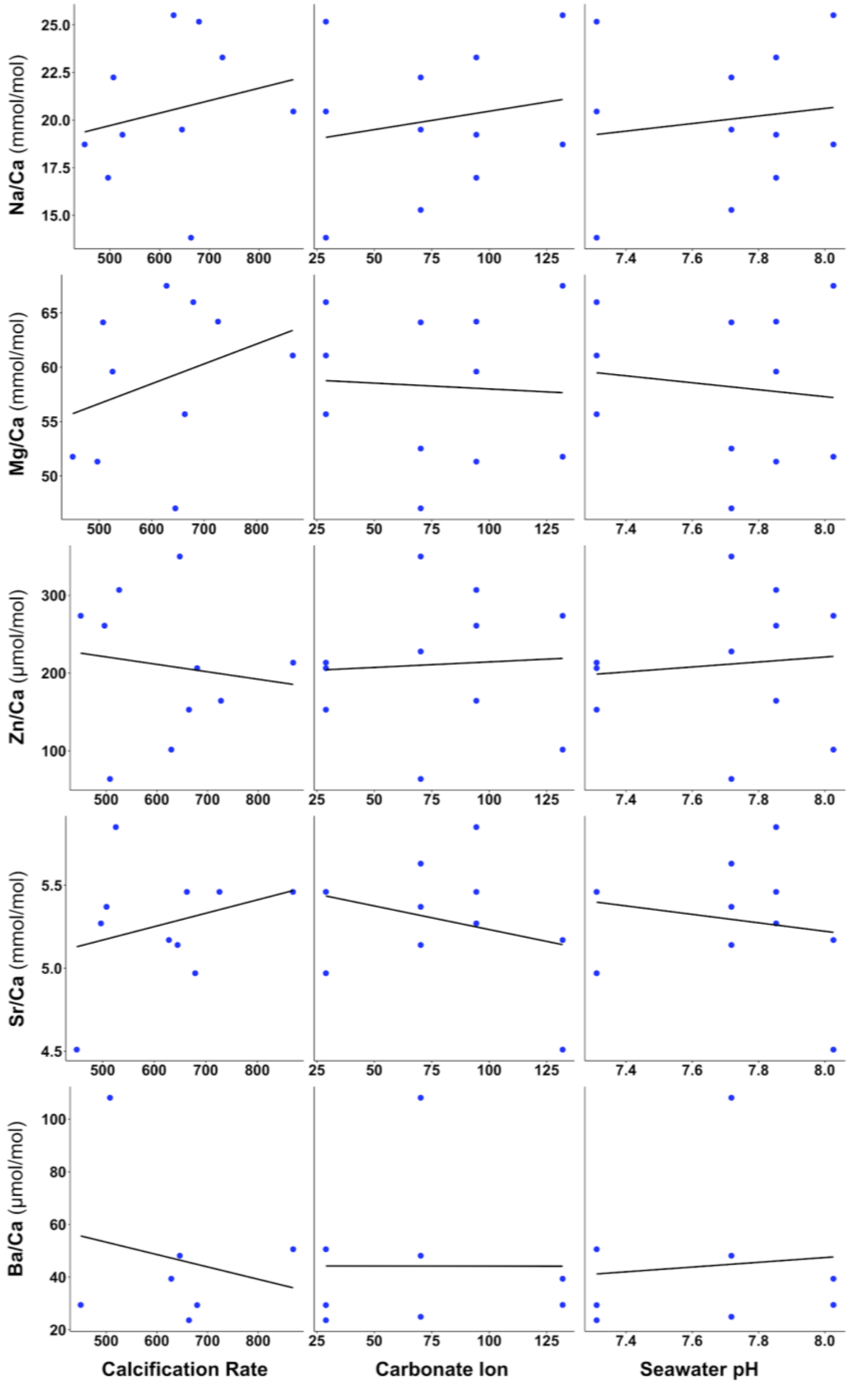

Figure S16. Array of scatterplots fitted with linear regressions displaying trace element-to-calcium ratios of the blue crab samples plotted against carbonate chemistry and other measured parameters. Bottom labels and labels going down the left side of the array signify x-axis parameters and y-axis parameters, respectively, for a given plot. Scatterplots with regressions that possess a p-value < 0.05 are outlined in red. No significant relationships are observed, potentially due to low sample replication.

Blue Crab – Quadratic Models

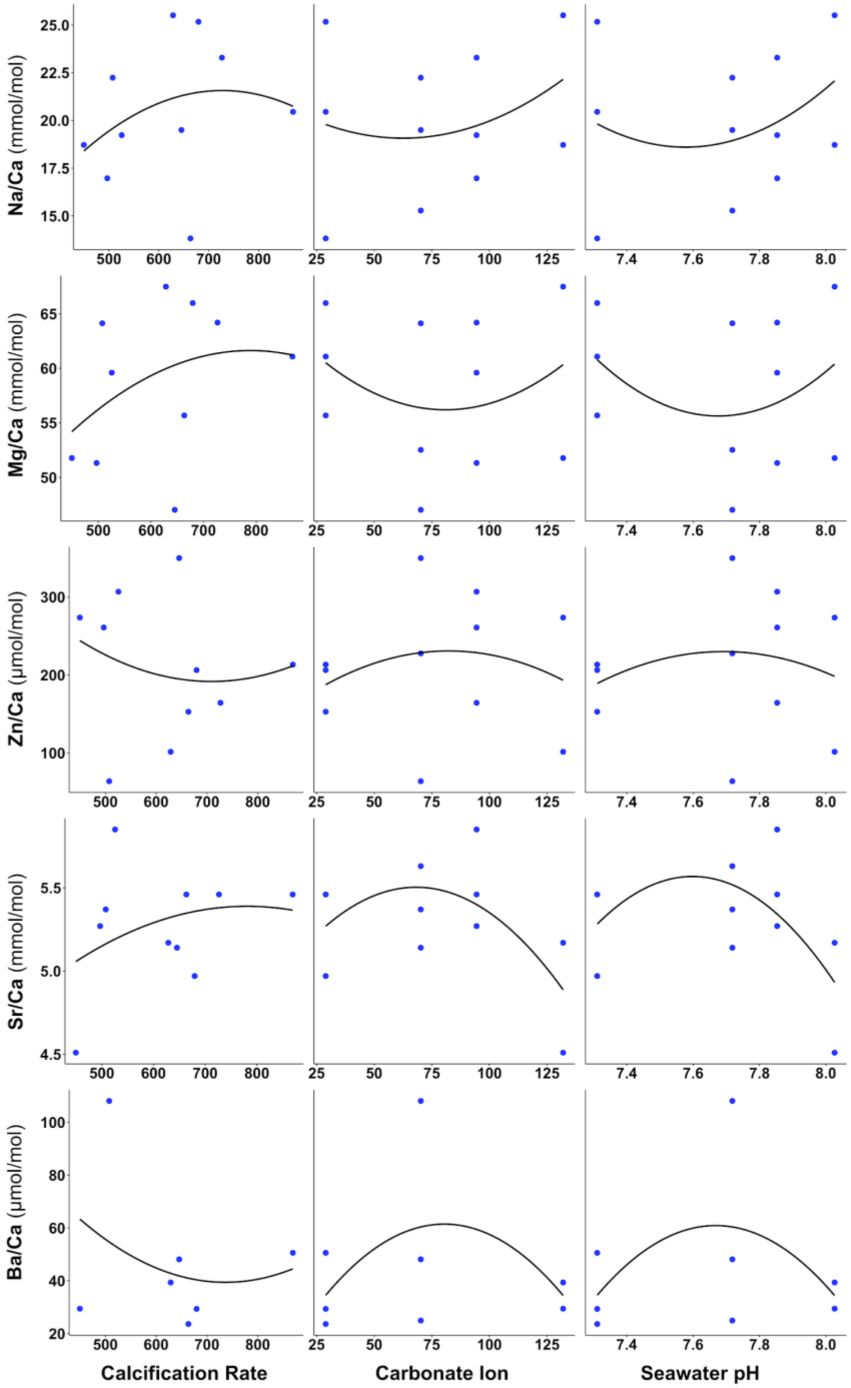

Figure S17. Array of scatterplots fitted with quadratic regressions displaying trace element-to-calcium ratios of the blue crab samples plotted against carbonate chemistry and other measured parameters. Bottom labels and labels going down the left side of the array signify x-axis parameters and y-axis parameters, respectively, for a given plot. Scatterplots with regressions that possess a p-value < 0.05 are outlined in red. No significant relationships are observed, potentially due to low sample replication.

Blue Mussel – Linear Models

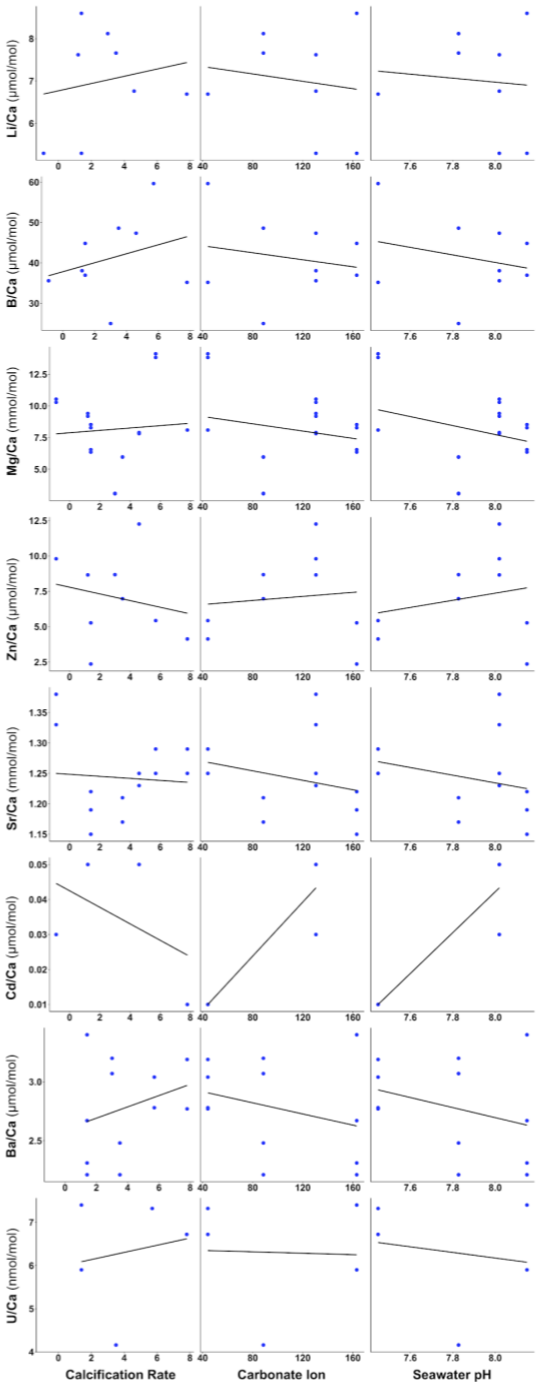

Figure S18. Array of scatterplots fitted with linear regressions displaying trace element-to-calcium ratios of the blue mussel samples plotted against carbonate chemistry and other measured parameters. Bottom labels and labels going down the left side of the array signify x-axis parameters and y-axis parameters, respectively, for a given plot. Scatterplots with regressions that possess a p-value < 0.05 are outlined in red. No significant relationships are observed, potentially due to low sample replication.

Blue Mussel – Quadratic Models

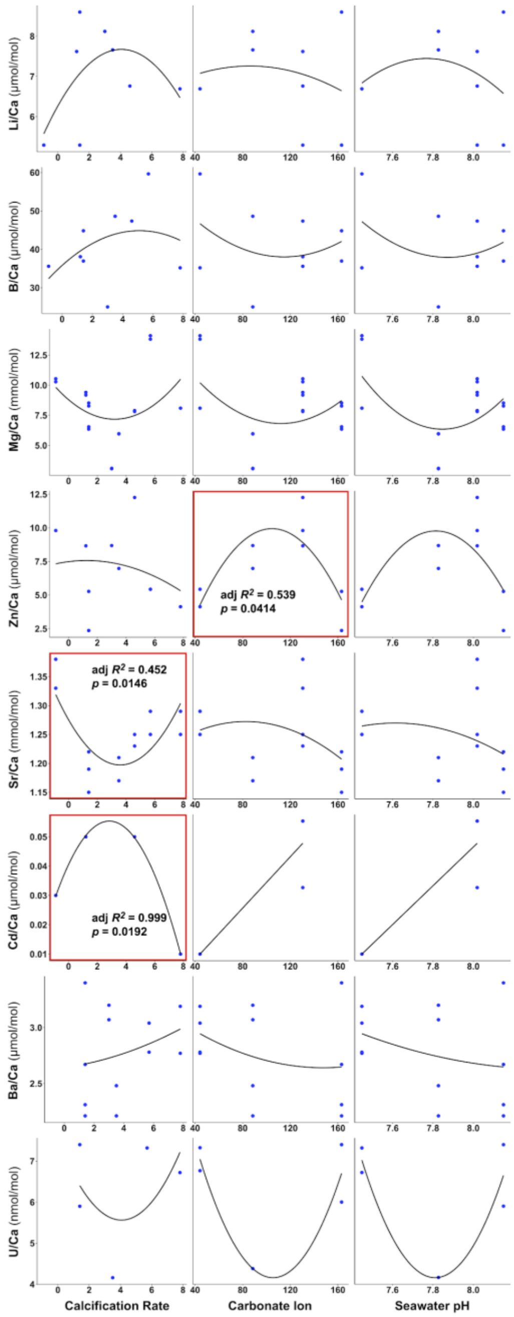

Figure S19. Array of scatterplots fitted with quadratic regressions displaying trace element-to-calcium ratios of the blue mussel samples plotted against carbonate chemistry and other measured parameters. Bottom labels and labels going down the left side of the array signify x-axis parameters and y-axis parameters, respectively, for a given plot. Scatterplots with regressions that possess a p-value < 0.05 are outlined in red. Significant relationships include Sr/Ca and Cd/Ca against net calcification rate and Zn/Ca against the seawater carbonate ion concentration. All significant relationships observed here are absent in the linear regression analysis.

Conch – Linear Models

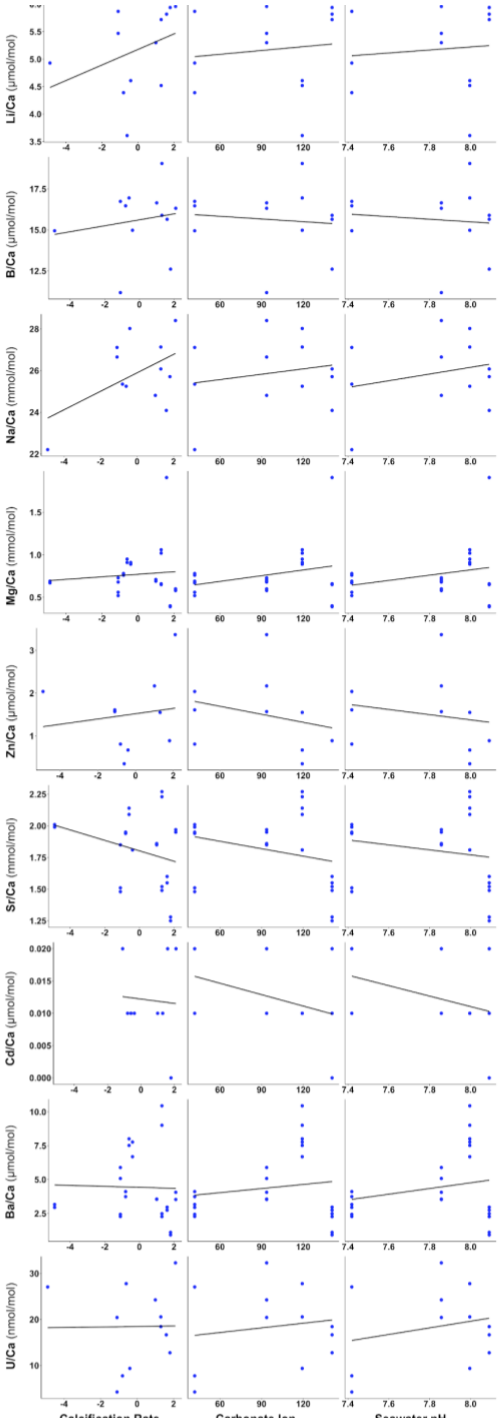

Figure S20. Array of scatterplots fitted with linear regressions displaying trace element-to-calcium ratios of the conch samples plotted against carbonate chemistry and other measured parameters. Bottom labels and labels going down the left side of the array signify x-axis parameters and y-axis parameters, respectively, for a given plot. Scatterplots with regressions that possess a p-value < 0.05 are outlined in red. No significant relationships are observed, potentially due to low sample replication.

Conch – Quadratic Models

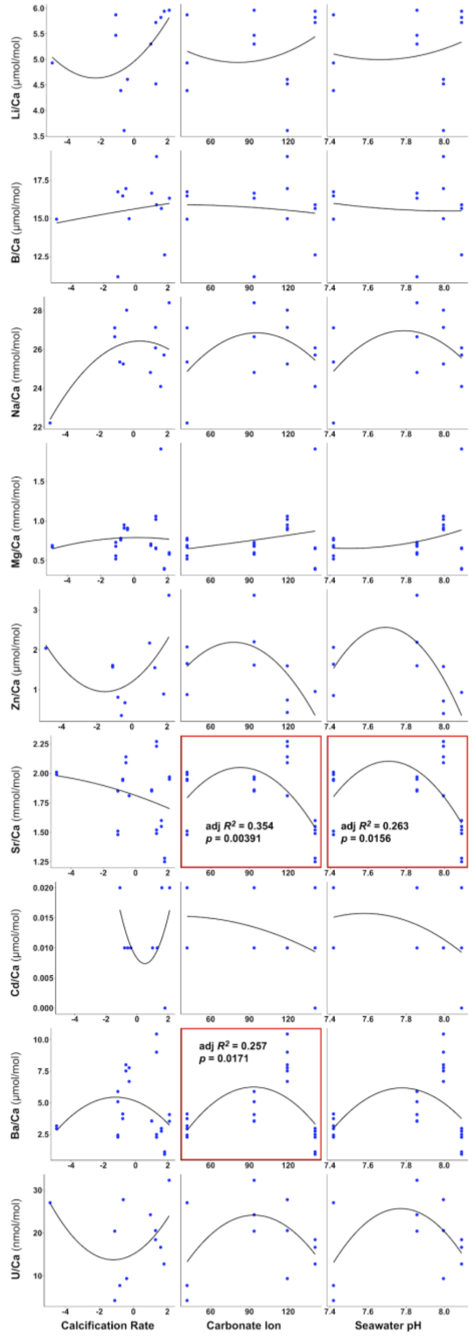

Figure S21. Array of scatterplots fitted with quadratic regressions displaying trace element-to-calcium ratios of the conch samples plotted against carbonate chemistry and other measured parameters. Bottom labels and labels going down the left side of the array signify x-axis parameters and y-axis parameters, respectively, for a given plot. Scatterplots with regressions that possess a p-value < 0.05 are outlined in red. Significant relationships observed include Sr/Ca against seawater carbonate ion concentration and seawater pH and also Ba/Ca against seawater carbonate ion concentrations. All significant patterns here are absent in the linear analysis.

Temperature Coral – Linear Models

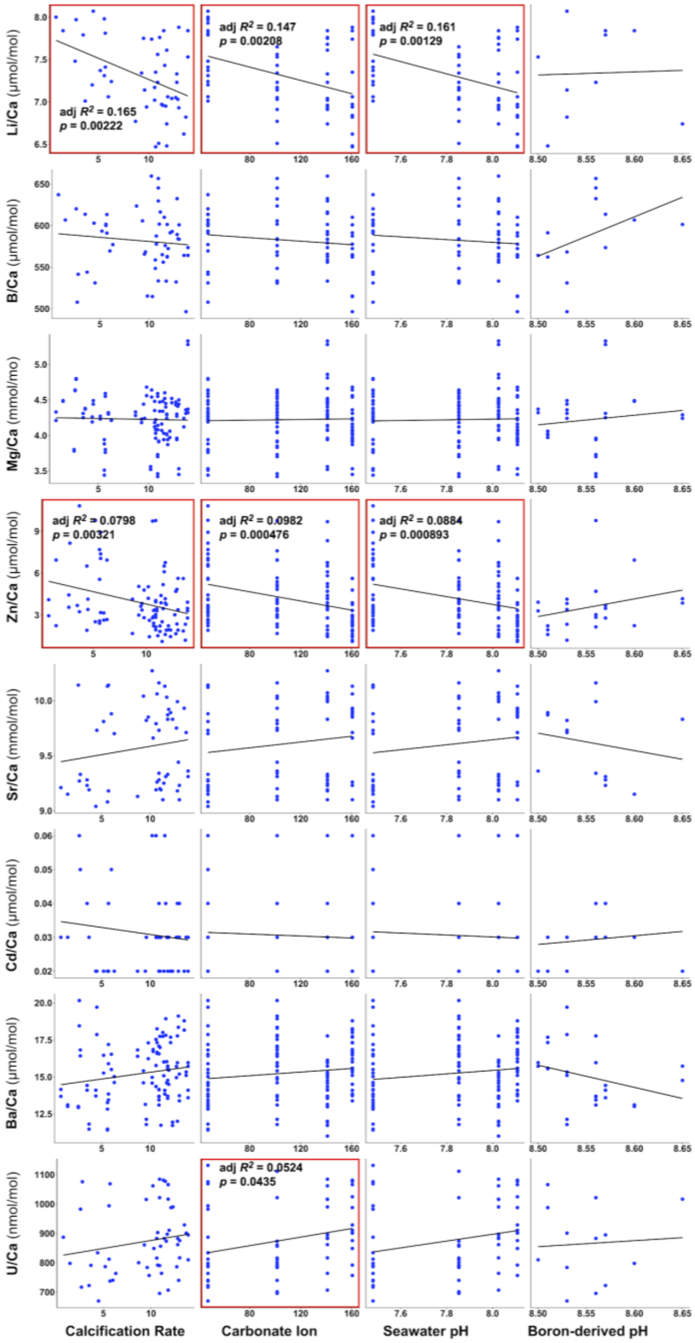

Figure S22. Array of scatterplots fitted with linear regressions displaying trace element-to-calcium ratios of temperate coral samples plotted against carbonate chemistry and other measured parameters. Bottom labels and labels going down the left side of the array signify x-axis parameters and y-axis parameters, respectively, for a given plot. Scatterplots with regressions that possess a p-value < 0.05 are outlined in red. Significant relationships include Li/Ca and Zn/Ca against net calcification rate, seawater carbonate ion concentration, and seawater pH and U/Ca against seawater carbonate ion concentration.

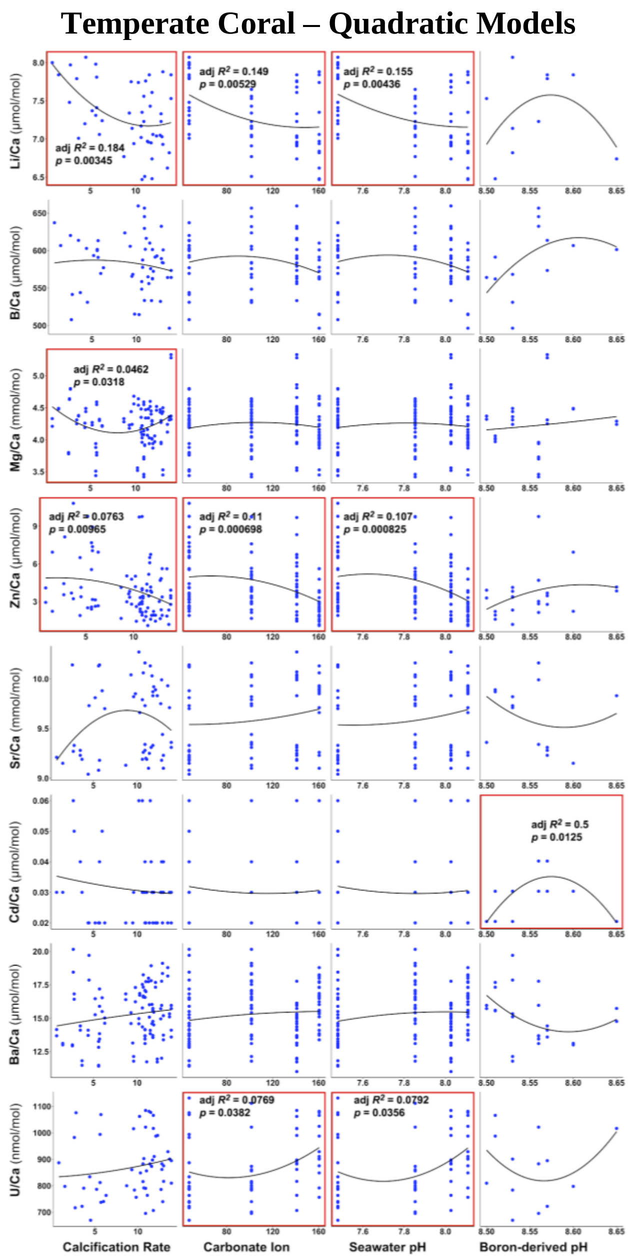

Figure S23. Array of scatterplots fitted with quadratic regressions displaying trace element-to-calcium ratios of temperate coral samples plotted against carbonate chemistry and other measured parameters. Bottom labels and labels going down the left side of the array signify x-axis parameters and y-axis parameters, respectively, for a given plot. Scatterplots with regressions that possess a p-value < 0.05 are outlined in red. Significant relationships include Li/Ca, Mg/Ca, and Zn/Ca against net calcification rate; Li/Ca, Zn/Ca, and U/Ca against seawater carbonate ion concentration and seawater pH; and Cd/Ca against boron-derived calcifying fluid pH. Significant relationships not present in the linear regression analysis include the Mg/Ca against net calcification rate; U/Ca again seawater pH; and Cd/Ca against boron-derived calcifying fluid pH.

Coralline red algae – Linear Models

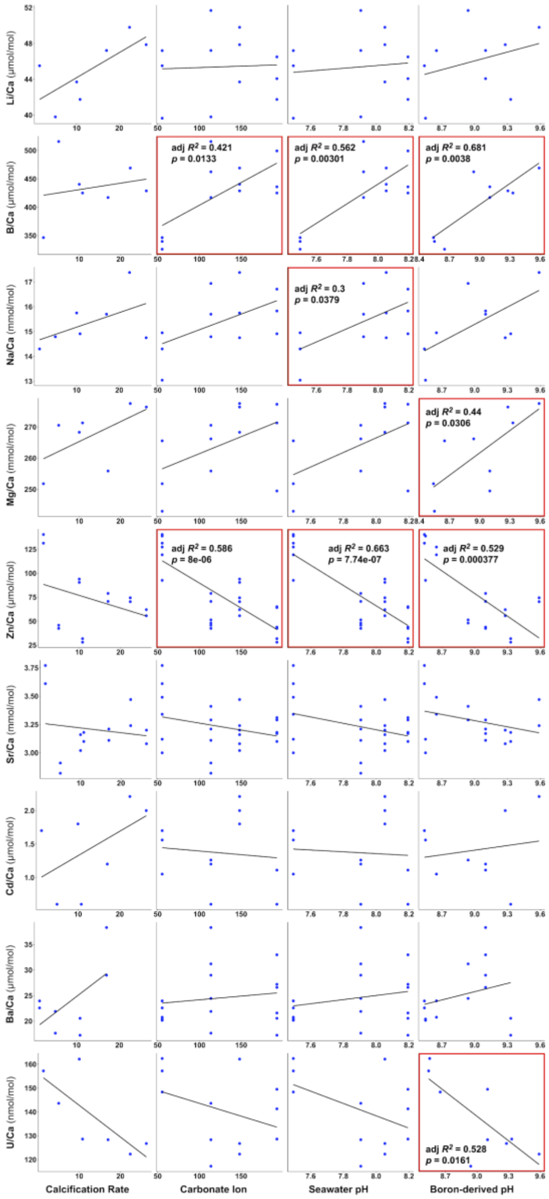

Figure S24. Array of scatterplots fitted with linear regressions displaying trace element-to-calcium ratios of coralline red algae samples plotted against carbonate chemistry and other measured parameters. Bottom labels and labels going down the left side of the array signify x-axis parameters and y-axis parameters, respectively, for a given plot. Scatterplots with regressions that possess a p-value < 0.05 are outlined in red. Significant relationships include B/Ca and Zn/Ca against Carbonate Ion; B/Ca, Mg/Ca, Zn/Ca, and Na/Ca against Alkalinity; B/Ca, Zn/Ca, and Na/Ca against Seawater pH; and B/Ca, Mg/Ca, Zn/Ca, Ba/Ca and U/Ca against Boron-derived pH. Significant relationships absent from the quadratic regression analysis include Na/Ca against Seawater pH; and Mg/Ca and Ba/Ca against Boron-derived pH.

Coralline Red Algae – Quadratic Models

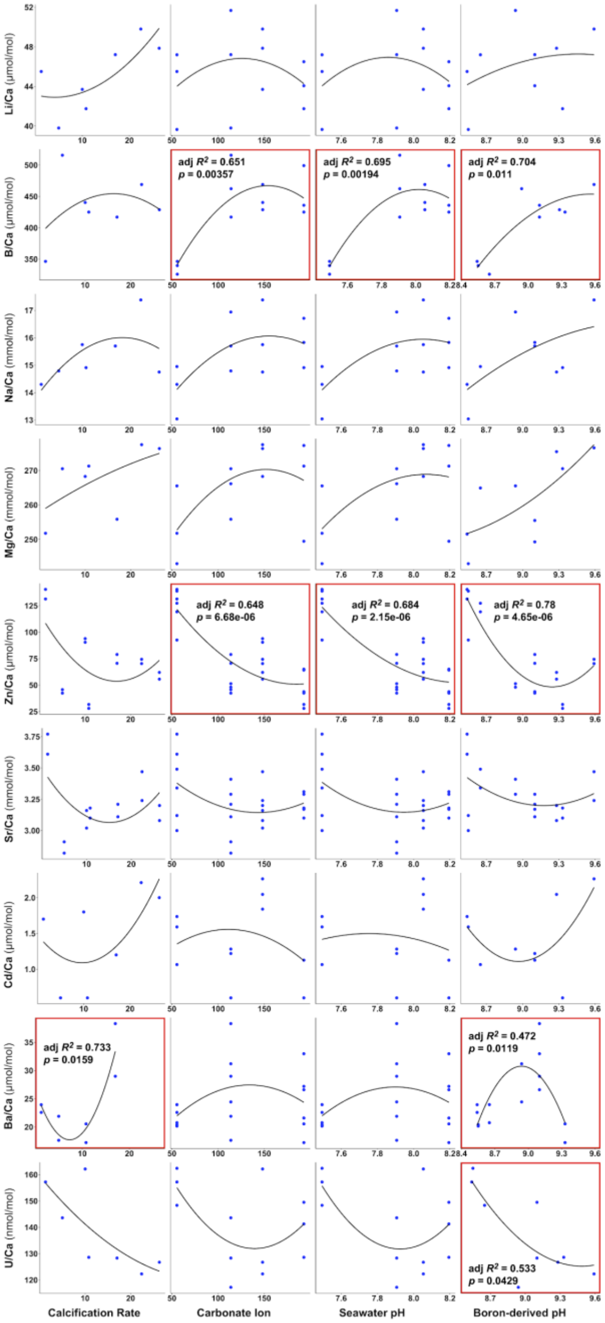

Figure S25. Array of scatterplots fitted with quadratic regressions displaying trace element-to-calcium ratios of coralline red algae samples plotted against carbonate chemistry and other measured parameters. Bottom labels and labels going down the left side of the array signify x-axis parameters and y-axis parameters, respectively, for a given plot. Scatterplots with regressions that possess a p-value < 0.05 are outlined in red. Significant relationships include Ba/Ca against net calcification rate; B/Ca and Zn/Ca against seawater carbonate ion concentration and seawater pH; and B/Ca, Zn/Ca, Ba/Ca, and U/Ca against boron-derived calcifying fluid pH.

Gulf shrimp – Linear Models

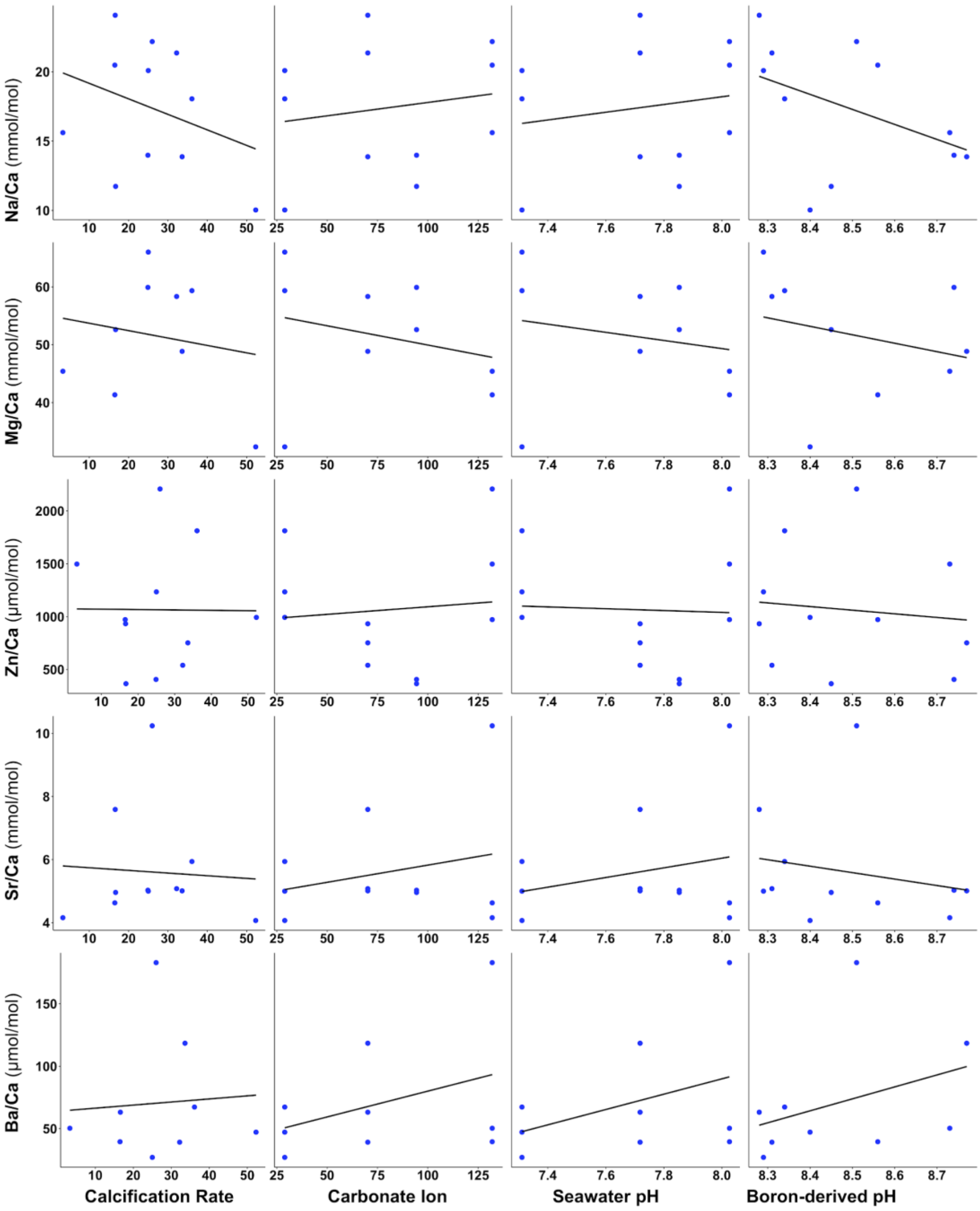

Figure S26. Array of scatterplots fitted with linear regressions displaying trace element-to-calcium ratios of the gulf shrimp samples plotted against carbonate chemistry and other measured parameters. Bottom labels and labels going down the left side of the array signify x-axis parameters and y-axis parameters, respectively, for a given plot. Scatterplots with regressions that possess a p-value < 0.05 are outlined in red. No significant relationships are observed.

Gulf Shrimp – Quadratic Models

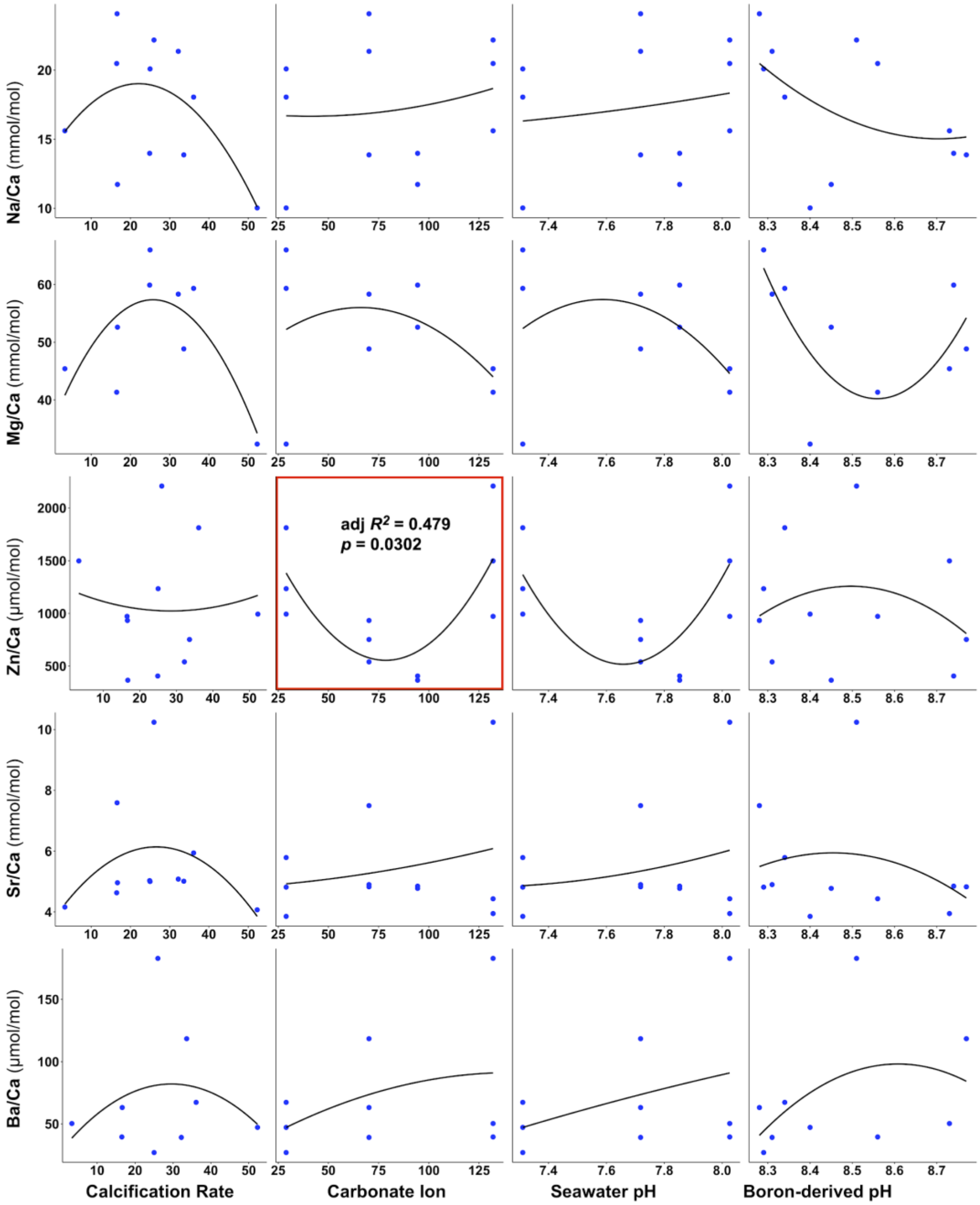

Figure S27. Array of scatterplots fitted with quadratic regressions displaying trace element-to-calcium ratios of the gulf shrimp samples plotted against carbonate chemistry and other measured parameters. Bottom labels and labels going down the left side of the array signify x-axis parameters and y-axis parameters, respectively, for a given plot. Scatterplots with regressions that possess a p-value < 0.05 are outlined in red. A significant relationship is observed for Zn/Ca against the seawater carbonate ion concentration, which is not observed in the linear regression analysis.

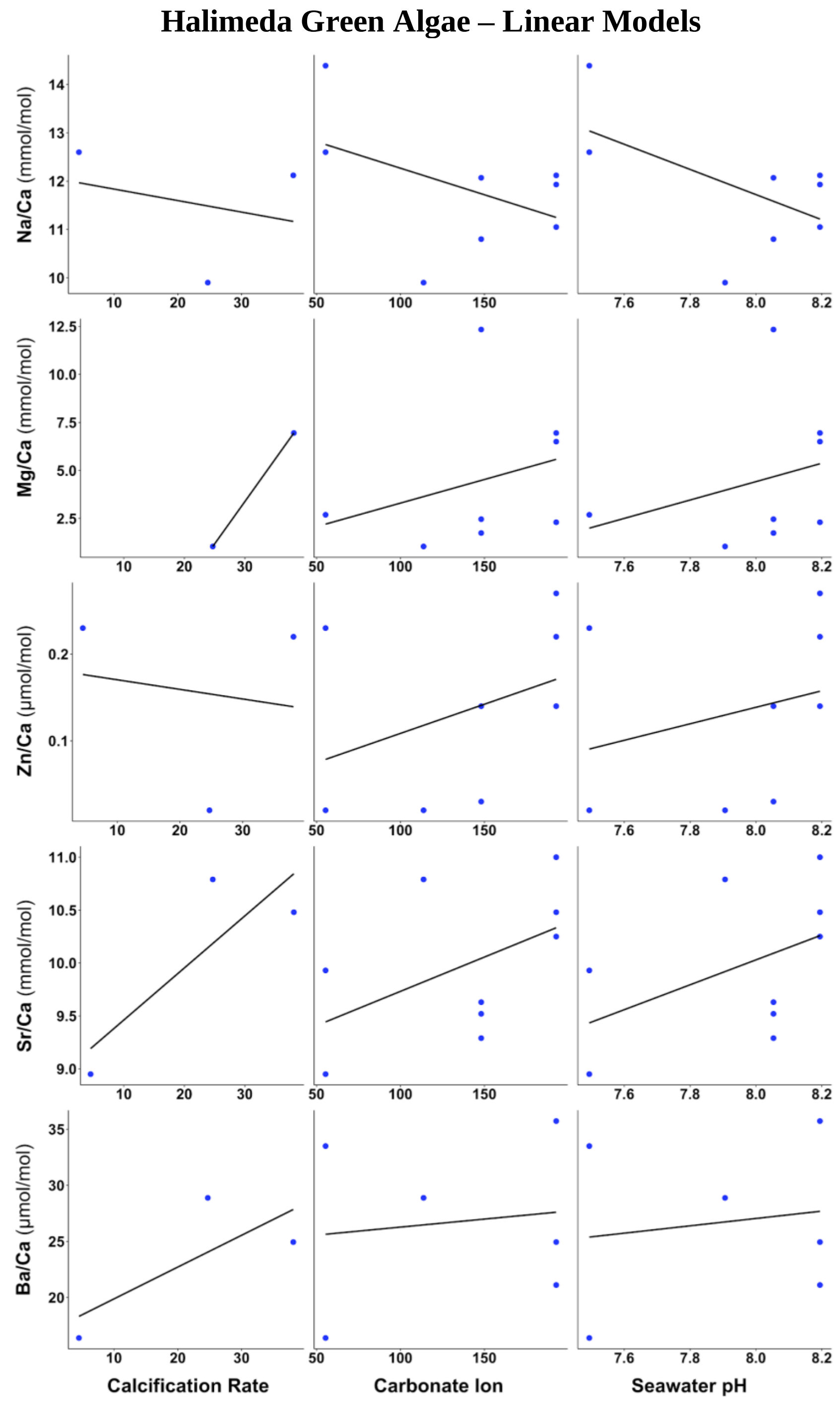

Figure S28. Array of scatterplots fitted with linear regressions displaying trace element-to-calcium ratios of the halimeda green algae samples plotted against carbonate chemistry and other measured parameters. Bottom labels and labels going down the left side of the array signify x-axis parameters and y-axis parameters, respectively, for a given plot. Scatterplots with regressions that possess a p-value < 0.05 are outlined in red. No significant relationships are observed, potentially due to low sample replication.

Halimeda Green Algae – Quadratic Models

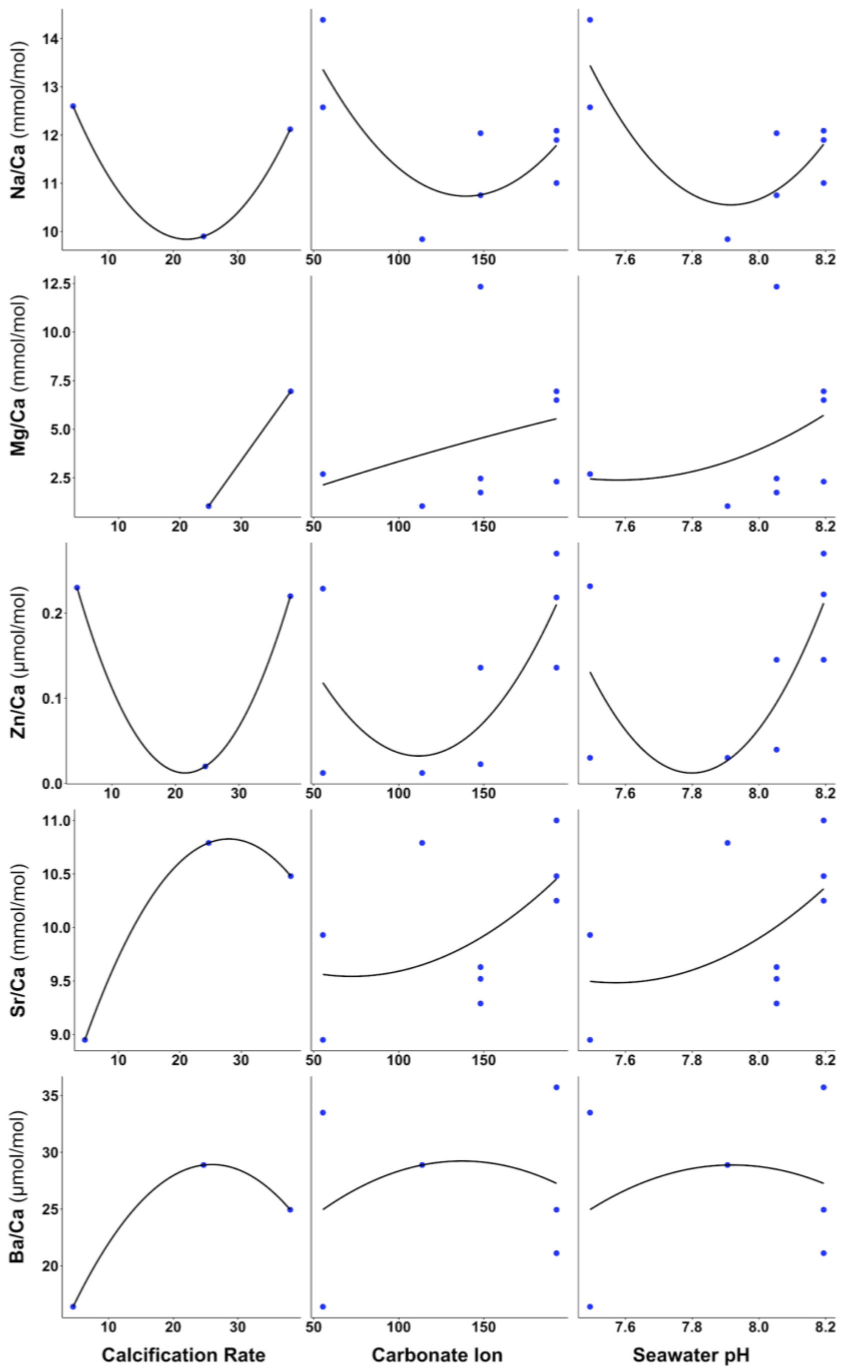

Figure 29. Array of scatterplots fitted with quadratic regressions displaying trace element-to-calcium ratios of the halimeda green algae samples plotted against carbonate chemistry and other measured parameters. Bottom labels and labels going down the left side of the array signify x-axis parameters and y-axis parameters, respectively, for a given plot. Scatterplots with regressions that possess a p-value < 0.05 are outlined in red. No significant relationships are observed, potentially due to low sample replication.

Hard Clam – Linear Models

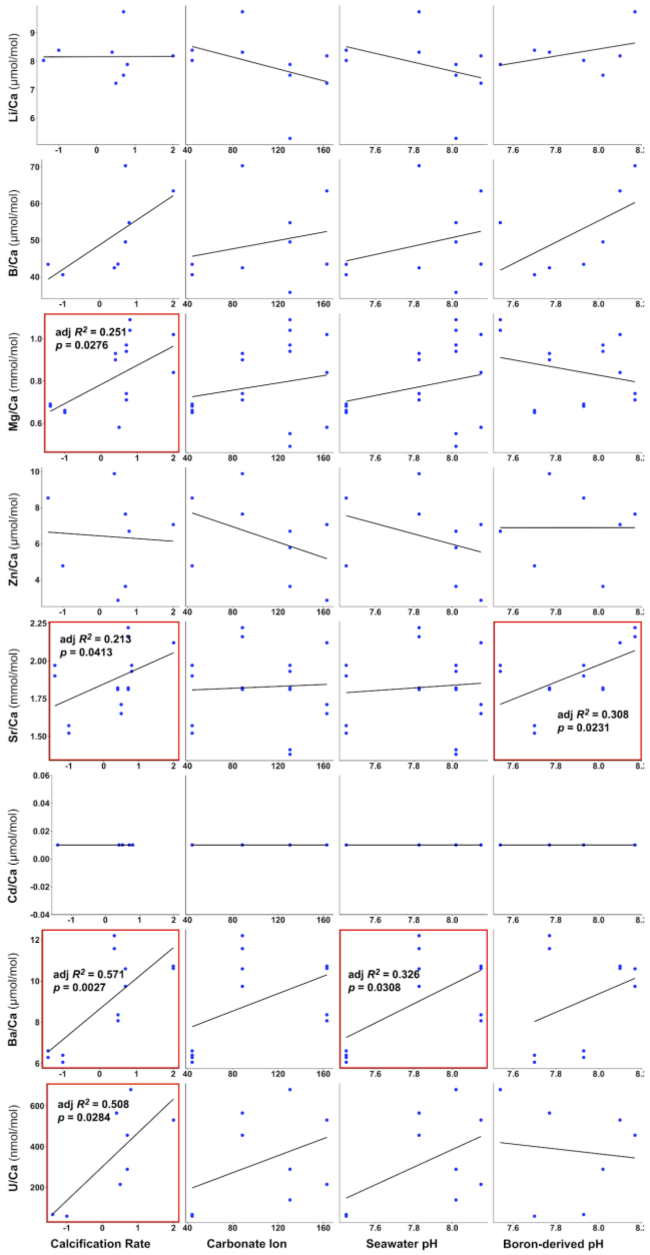

Figure S30. Array of scatterplots fitted with linear regressions displaying trace element-to-calcium ratios of the hard clam samples plotted against carbonate chemistry and other measured parameters. Bottom labels and labels going down the left side of the array signify x-axis parameters and y-axis parameters, respectively, for a given plot. Scatterplots with regressions that possess a p-value < 0.05 are outlined in red. Significant relationships include Mg/Ca, Sr/Ca, Ba/Ca, and U/Ca with net calcification rate; Ba/Ca with seawater pH; and Sr/Ca with boron-derived calcifying fluid pH. The relationships observed here that are absent from the quadratic analysis include Ba/Ca against the seawater carbonate ion concentration and Mg/Ca, Sr/Ca, and U/Ca against the net calcification rate.

Hard Clam – Quadratic Models

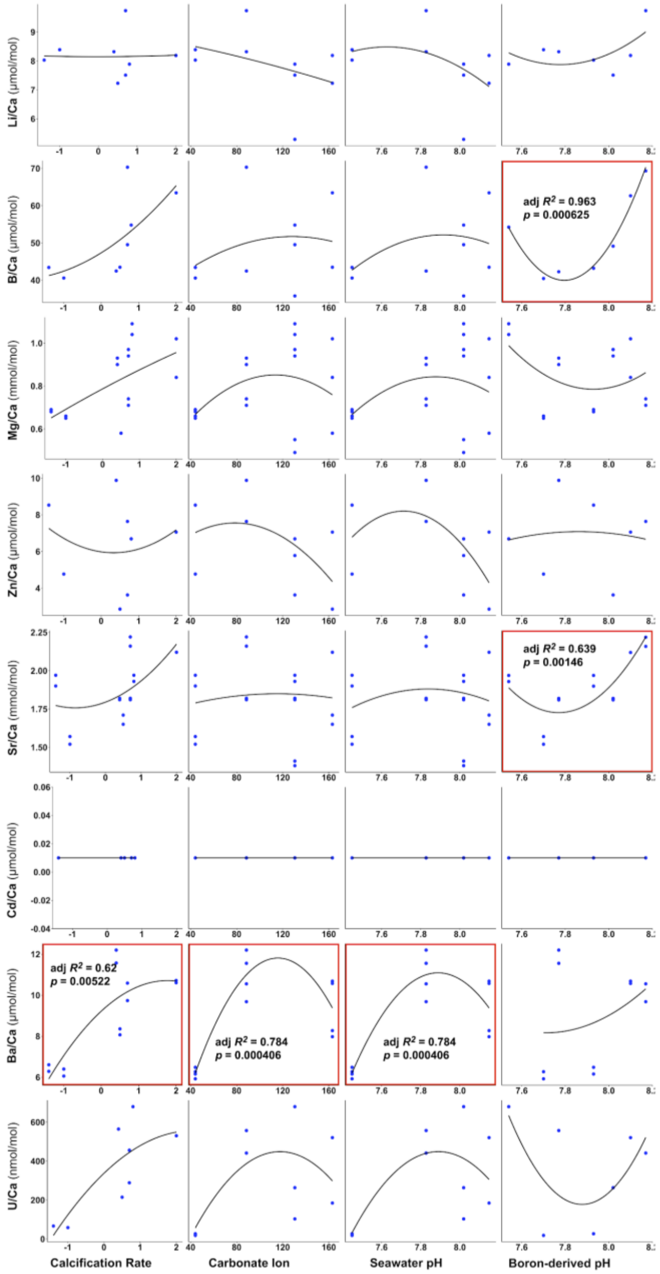

Figure S31. Array of scatterplots fitted with quadratic regressions displaying trace element-to-calcium ratios of the hard clam samples plotted against carbonate chemistry and other measured parameters. Bottom labels and labels going down the left side of the array signify x-axis parameters and y-axis parameters, respectively, for a given plot. Scatterplots with regressions that possess a p-value < 0.05 are outlined in red. Significant relationships include Ba/Ca with net calcification rate, seawater carbonate ion concentration, and seawater pH as well as B/Ca and Sr/Ca with boron-derived calcifying fluid pH. The relationships absent in the linear regression analysis include the Ba/Ca against seawater carbonate ion concentrations and B/Ca again the boron-derived calcifying fluid pH.

Limpet – Linear Models

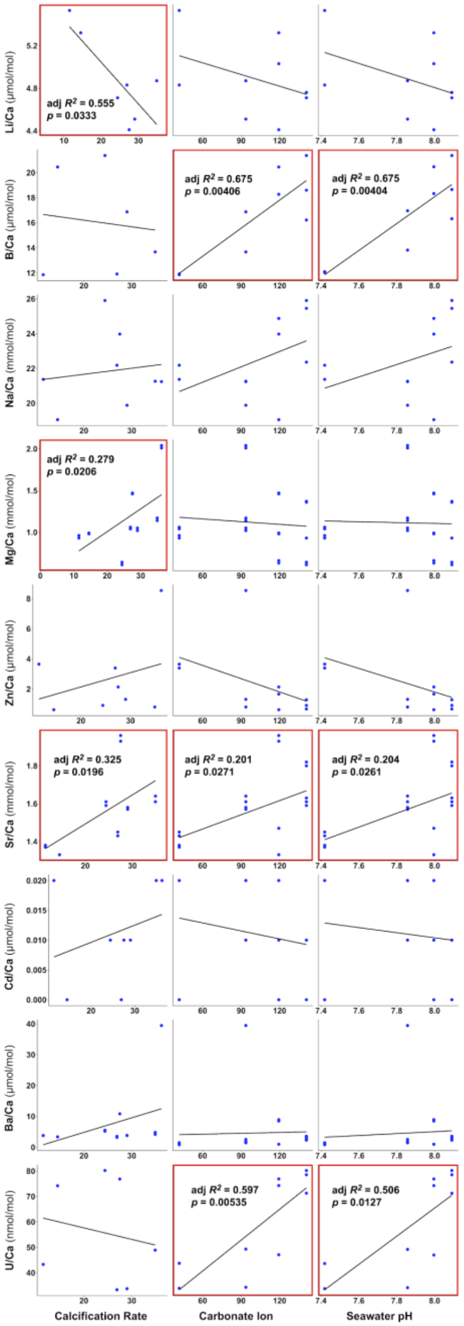

Figure S32. Array of scatterplots fitted with linear regressions displaying trace element-to-calcium ratios of the limpet samples plotted against carbonate chemistry and other measured parameters. Bottom labels and labels going down the left side of the array signify x-axis parameters and y-axis parameters, respectively, for a given plot. Scatterplots with regressions that possess a p-value < 0.05 are outlined in red. Significant relationships include Li/Ca, Mg/Ca, and Sr/Ca against net calcification rate and B/Ca, Sr/Ca, and U/Ca against seawater carbonate ion concentration and seawater pH. The significant relationships between Sr/Ca and seawater carbonate ion concentration and pH are not present in the quadratic analysis.

Limpet – Quadratic Models

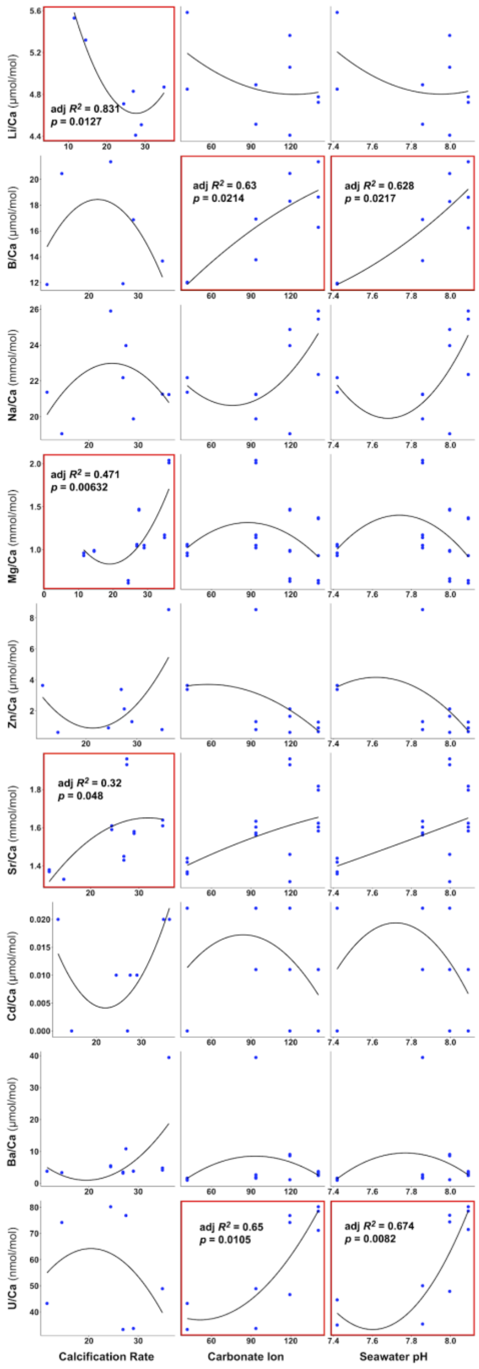

Figure S33. Array of scatterplots fitted with quadratic regressions displaying trace element-to-calcium ratios of the limpet samples plotted against carbonate chemistry and other measured parameters. Bottom labels and labels going down the left side of the array signify x-axis parameters and y-axis parameters, respectively, for a given plot. Scatterplots with regressions that possess a p-value < 0.05 are outlined in red. Significant relationships include Li/Ca, Mg/Ca, and Sr/Ca against net calcification rate and B/Ca and U/Ca against seawater carbonate ion concentration and seawater pH.

Pencil Urchin – Linear Models

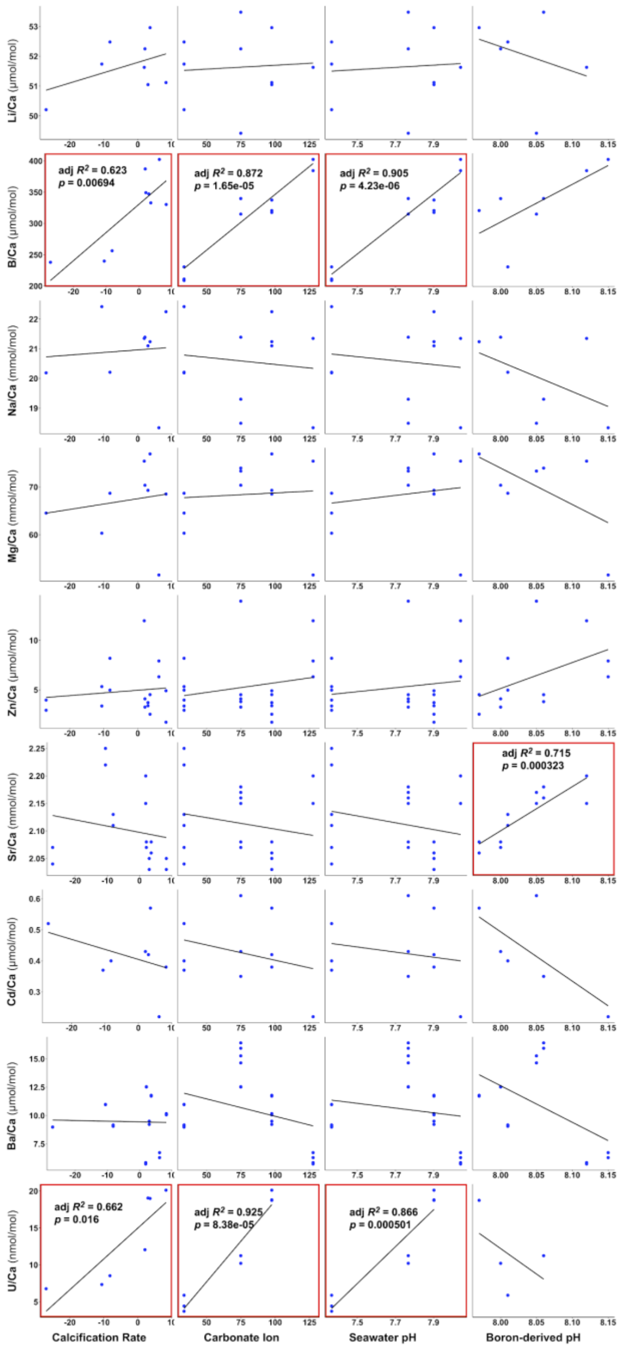

Figure S34. Array of scatterplots fitted with linear regressions displaying trace element-to-calcium ratios of pencil urchin samples plotted against carbonate chemistry and other measured parameters. Bottom labels and labels going down the left side of the array signify x-axis parameters and y-axis parameters, respectively, for a given plot. Scatterplots with regressions that possess a p-value < 0.05 are outlined in red. Significant relationships include B/Ca and U/Ca against net calcification rate, seawater carbonate ion concentration, and seawater pH and Sr/Ca against boron-derived calcifying fluid pH.

Pencil Urchin – Quadratic Models

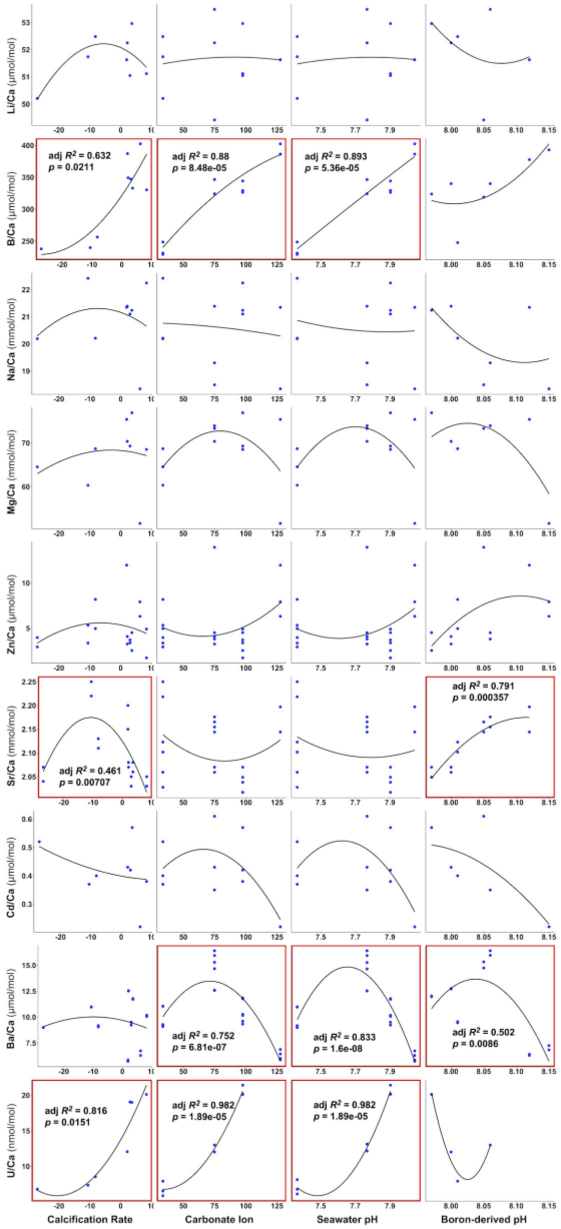

Figure S35. Array of scatterplots fitted with linear regressions displaying trace element-to-calcium ratios of pencil urchin samples plotted against carbonate chemistry and other measured parameters. Bottom labels and labels going down the left side of the array signify x-axis parameters and y-axis parameters, respectively, for a given plot. Scatterplots with regressions that possess a p-value < 0.05 are outlined in red. Significant relationships include B/Ca, Sr/Ca, and U/Ca against net calcification rate; B/Ca, Ba/Ca, and U/Ca against seawater carbonate ion concentration and seawater pH; and Sr/Ca and Ba/Ca against boron-derived calcifying fluid pH. Significant relationships absent from the linear regression analysis include Sr/Ca against net calcification rate and Ba/Ca against seawater carbonate ion concentration, seawater pH, and boron-derived calcifying fluid pH.

Periwinkle – Linear Models

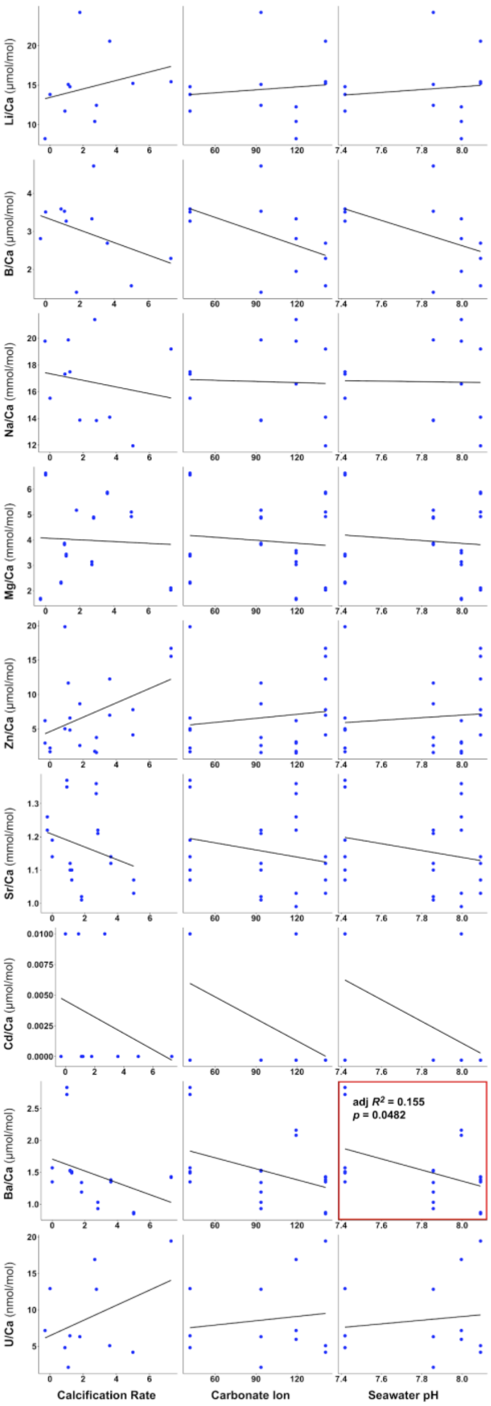

Figure S36. Array of scatterplots fitted with linear regressions displaying trace element-to-calcium ratios of periwinkle samples plotted against carbonate chemistry and other measured parameters. Bottom labels and labels going down the left side of the array signify x-axis parameters and y-axis parameters, respectively, for a given plot. Scatterplots with regressions that possess a p-value < 0.05 are outlined in red. The significant relationship of Ba/Ca against seawater pH is observed here, but not in the quadratic analysis.

Periwinkle – Quadratic Models

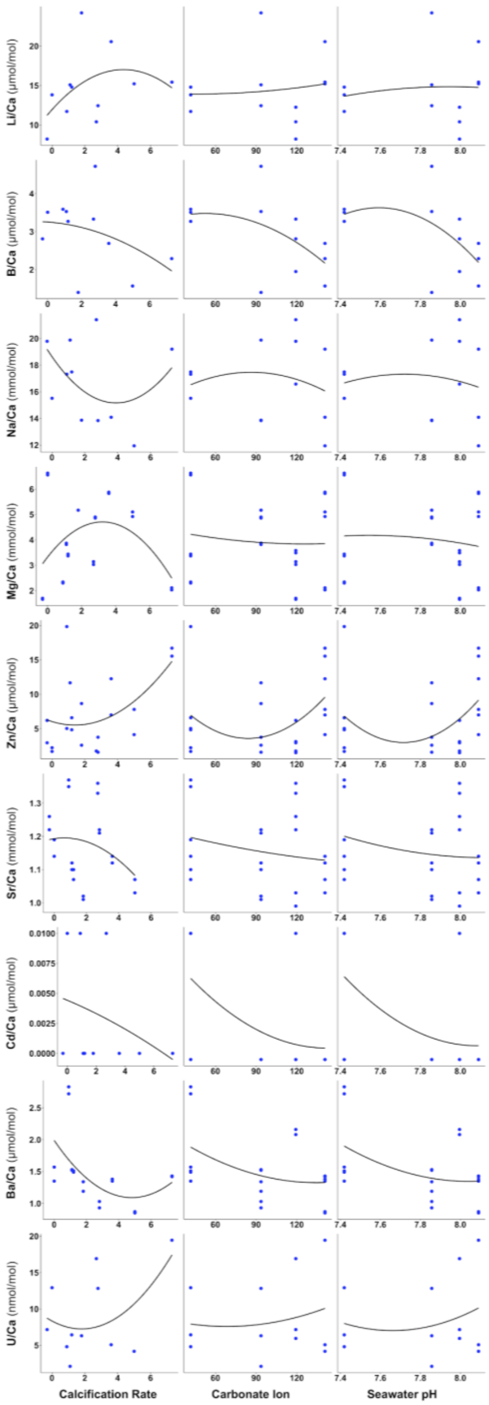

Figure S37. Array of scatterplots fitted with quadratic regressions displaying trace element-to-calcium ratios of periwinkle samples plotted against carbonate chemistry and other measured parameters. Bottom labels and labels going down the left side of the array signify x-axis parameters and y-axis parameters, respectively, for a given plot. Scatterplots with regressions that possess a p-value < 0.05 are outlined in red. No significant relationships are observed.

Purple Urchin – Linear Models

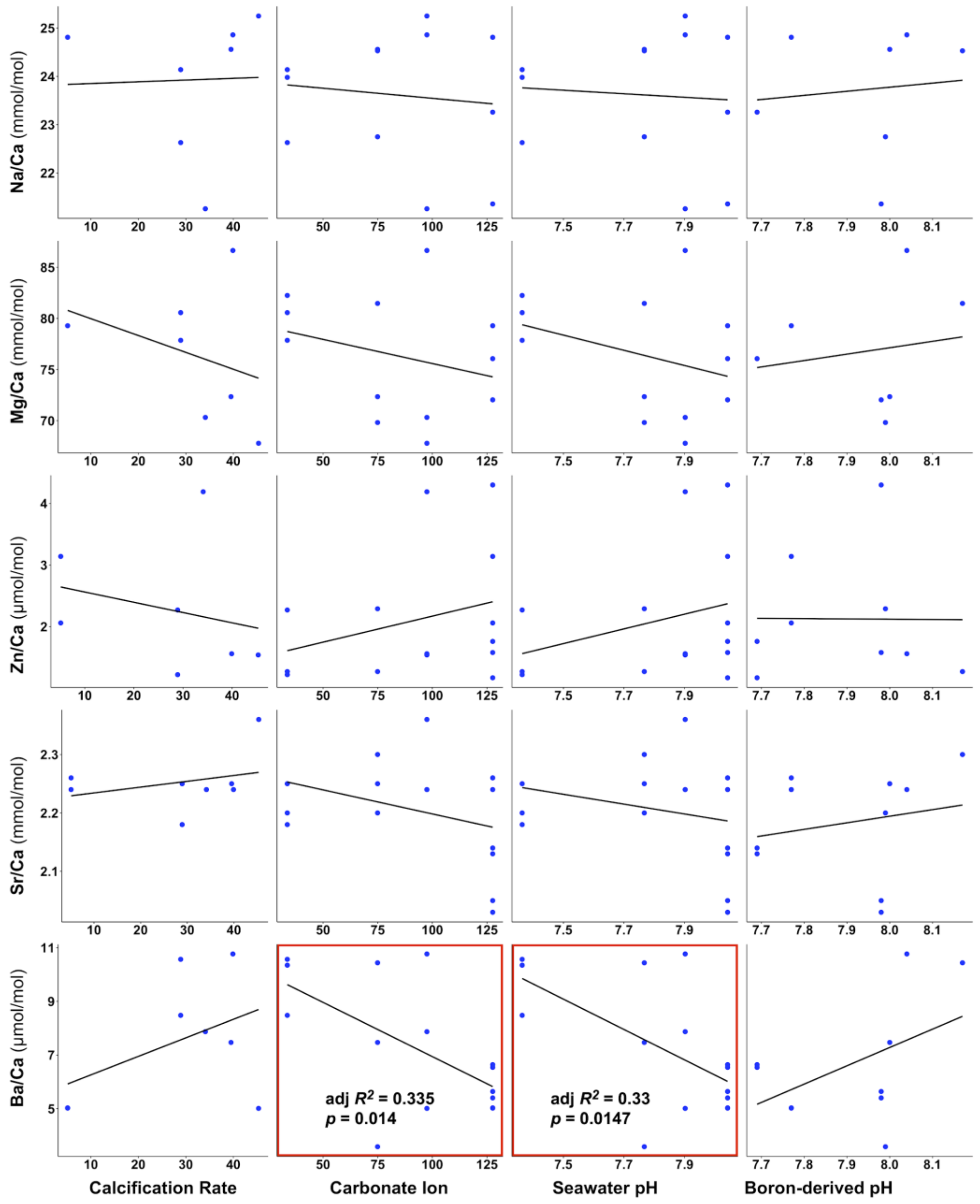

Figure S38. Array of scatterplots fitted with linear regressions displaying trace element-to-calcium ratios of the temperate purple urchin samples plotted against carbonate chemistry and other measured parameters. Bottom labels and labels going down the left side of the array signify x-axis parameters and y-axis parameters, respectively, for a given plot. Scatterplots with regressions that possess a p-value < 0.05 are outlined in red. Significant relationships are observed for Ba/Ca against seawater carbonate ion concentration and seawater pH; neither are observed in the quadratic analysis.

Purple Urchin – Quadratic Models

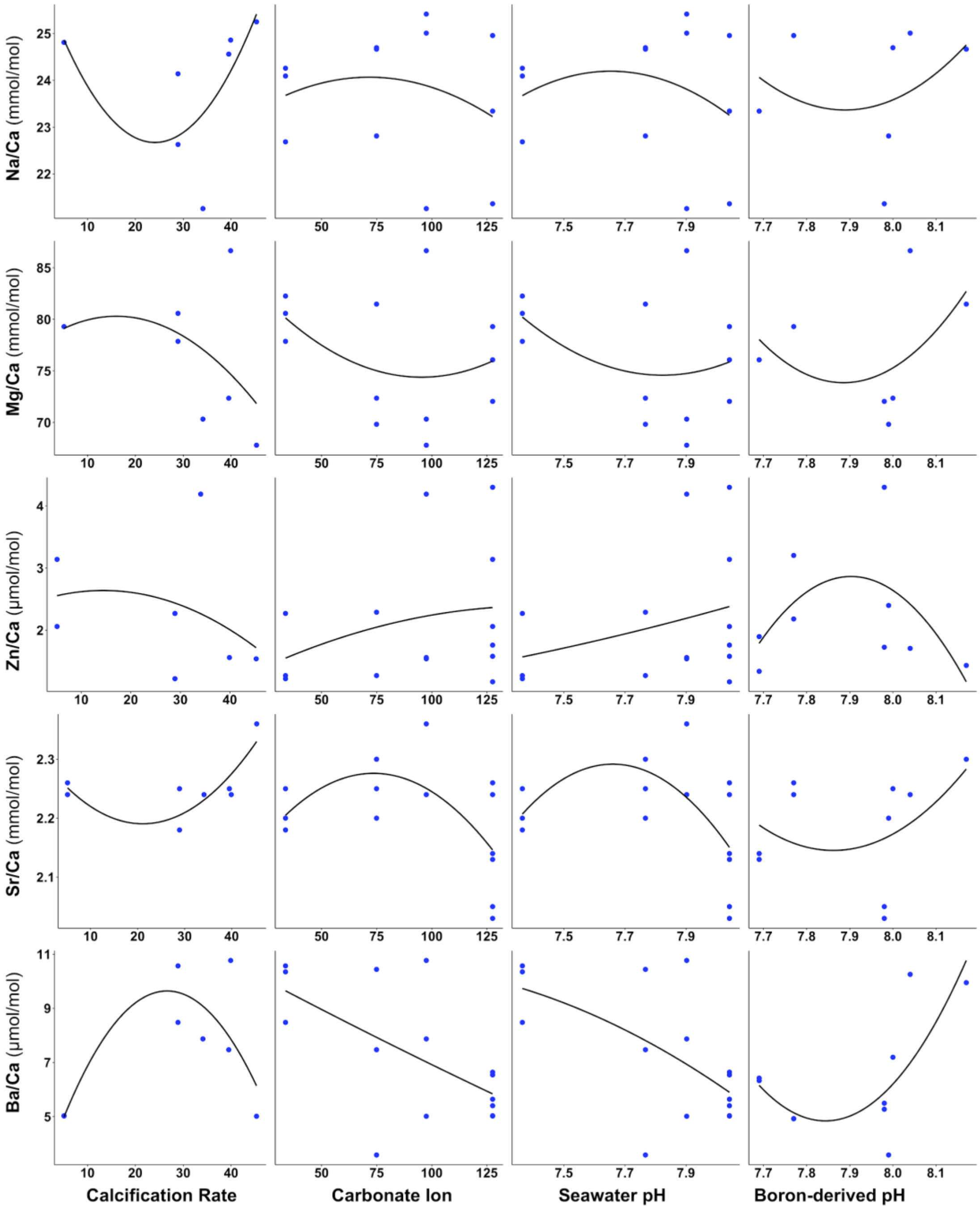

Figure S39. Array of scatterplots fitted with quadratic regressions displaying trace element-to-calcium ratios of the temperature purple urchin samples plotted against carbonate chemistry and other measured parameters. Bottom labels and labels going down the left side of the array signify x-axis parameters and y-axis parameters, respectively, for a given plot. Scatterplots with regressions that possess a p-value < 0.05 are outlined in red. No significant relationships are observed.

Serpulid worm – Linear Models

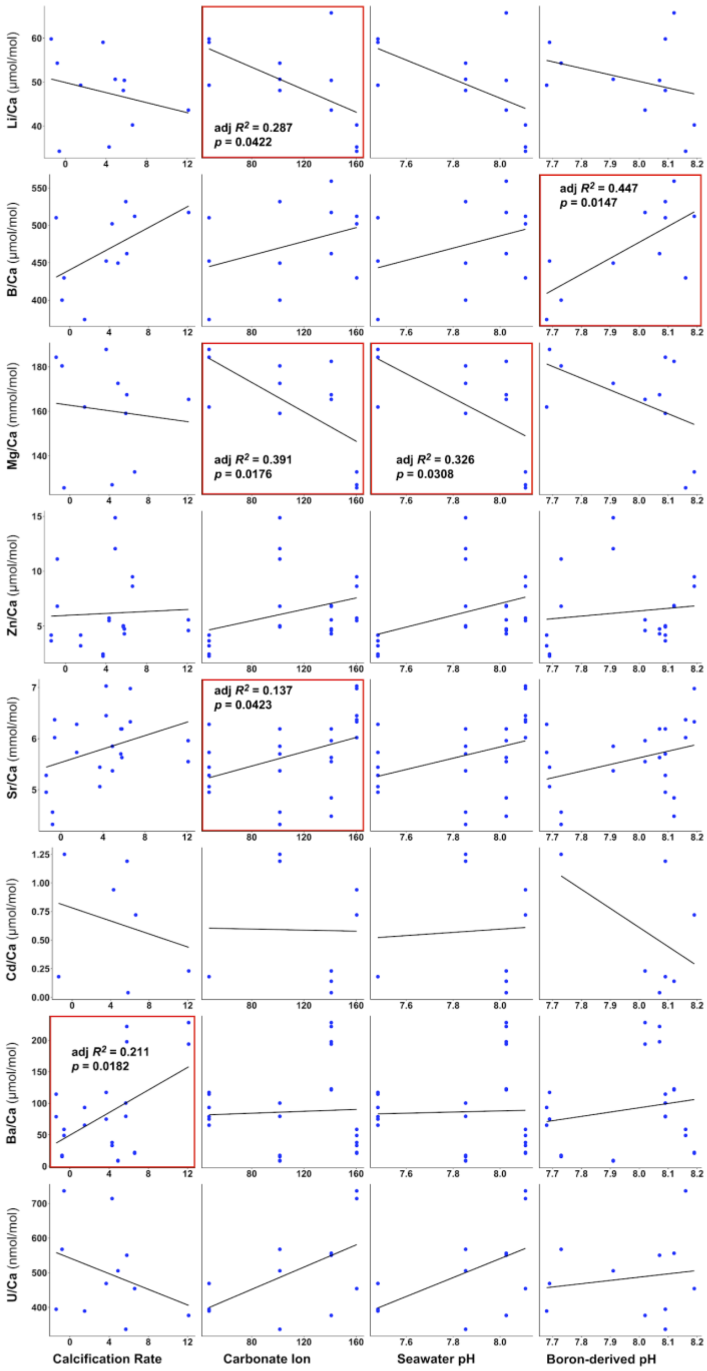

Figure S40. Array of scatterplots fitted with linear regressions displaying trace element-to-calcium ratios of serpulid worm samples plotted against carbonate chemistry and other measured parameters. Bottom labels and labels going down the left side of the array signify x-axis parameters and y-axis parameters, respectively, for a given plot. Scatterplots with regressions that possess a p-value < 0.05 are outlined in red. Significant relationships include Ba/Ca against net calcification rate; Li/Ca, Mg/Ca, and Sr/Ca against seawater carbonate ion concentration; Mg/Ca against seawater pH; and B/Ca against boron-derived calcifying fluid pH.

Serpulid Worm – Quadratic Models

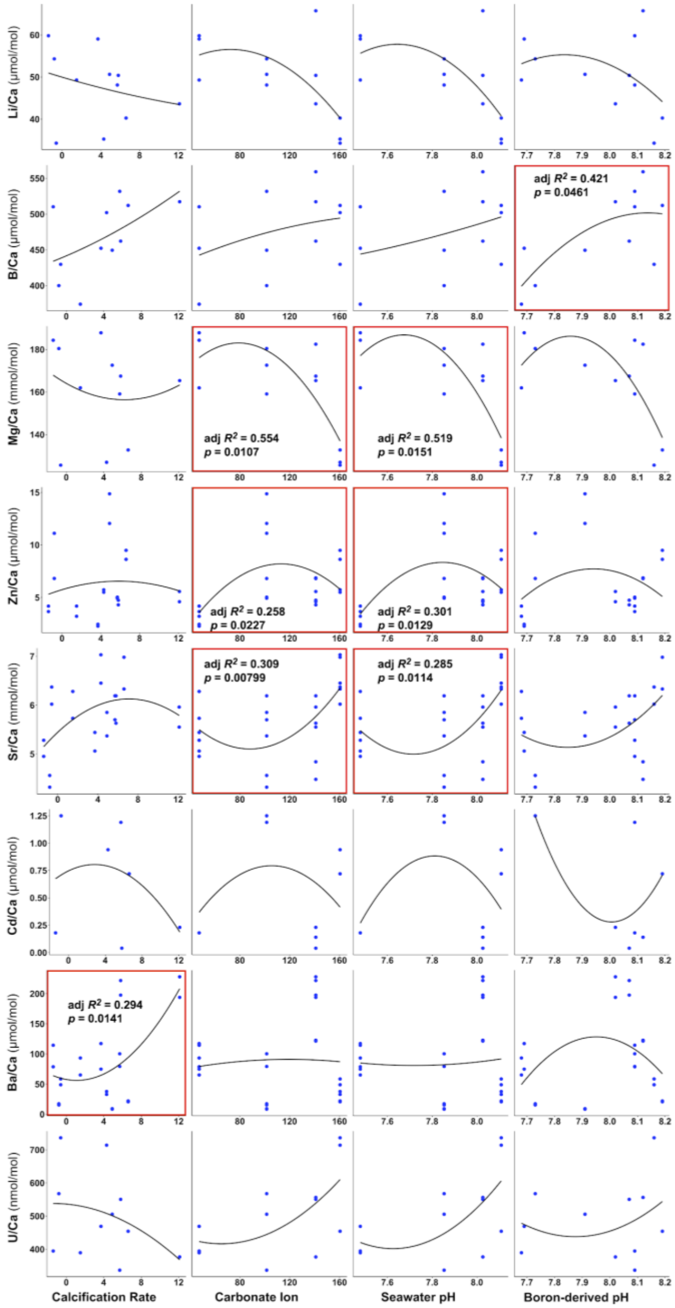

Figure S41. Array of scatterplots fitted with quadratic regressions displaying trace element-to-calcium ratios of serpulid worm samples plotted against carbonate chemistry and other measured parameters. Bottom labels and labels going down the left side of the array signify x-axis parameters and y-axis parameters, respectively, for a given plot. Scatterplots with regressions that possess a p-value < 0.05 are outlined in red. Significant relationships include Ba/Ca against net calcification rate, Mg/Ca, Zn/Ca, and Sr/Ca again seawater carbonate ion concentration and pH; and B/Ca against boron-derived calcifying fluid pH.

Soft Clam – Linear Models

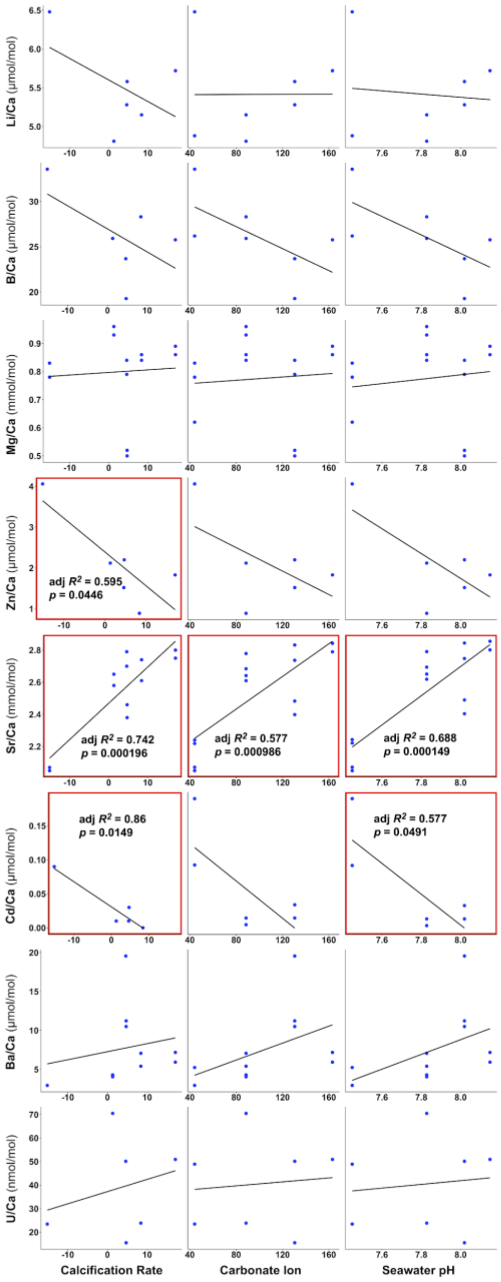

Figure S42. Array of scatterplots fitted with linear regressions displaying trace element-to-calcium ratios of soft clam samples plotted against carbonate chemistry and other measured parameters. Bottom labels and labels going down the left side of the array signify x-axis parameters and y-axis parameters, respectively, for a given plot. Scatterplots with regressions that possess a p-value < 0.05 are outlined in red. Significant relationships include Zn/Ca, Sr/Ca and Cd/Ca against net calcification rate; Sr/Ca against seawater carbonate ion concentration; and Sr/Ca and Cd/Ca against seawater pH. The relationships of Zn/Ca and Cd/Ca against net calcification rate as well as Cd/Ca against seawater pH are not observed in the quadratic regression analysis.

Soft Clam – Quadratic Models

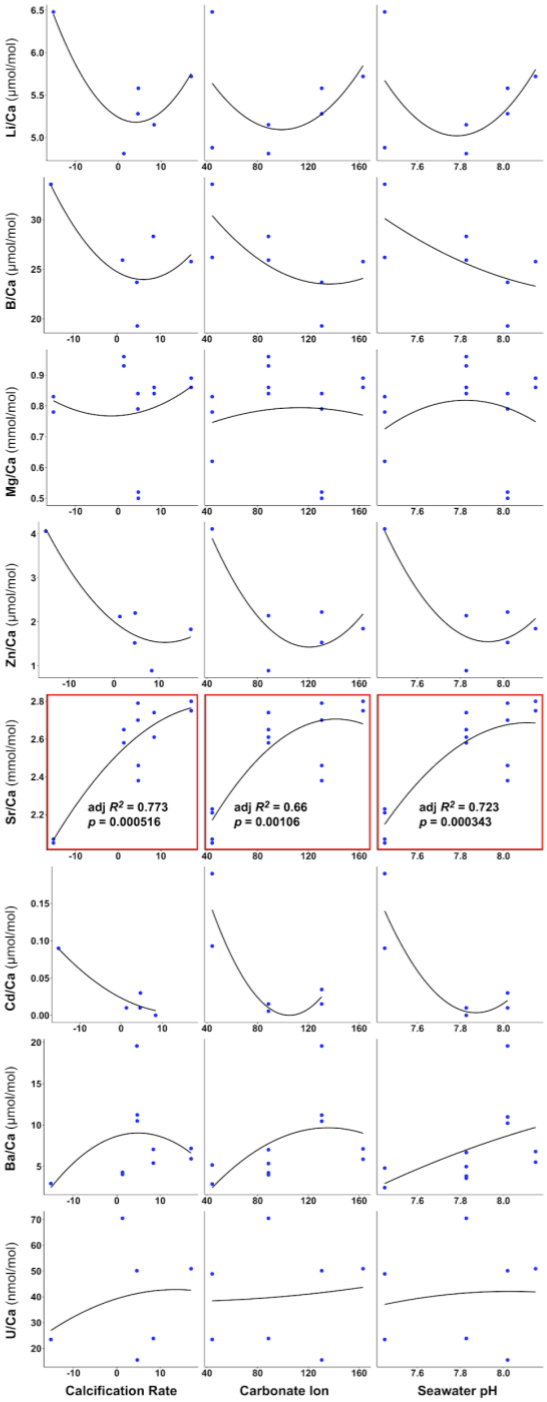

Figure S43. Array of scatterplots fitted with quadratic regressions displaying trace element-to-calcium ratios of soft clam samples plotted against carbonate chemistry and other measured parameters. Bottom labels and labels going down the left side of the array signify x-axis parameters and y-axis parameters, respectively, for a given plot. Scatterplots with regressions that possess a p-value < 0.05 are outlined in red. Significant relationships include Sr/Ca against net calcification rate, seawater carbonate ion concentration, and seawater pH.

Whelk – Linear Models

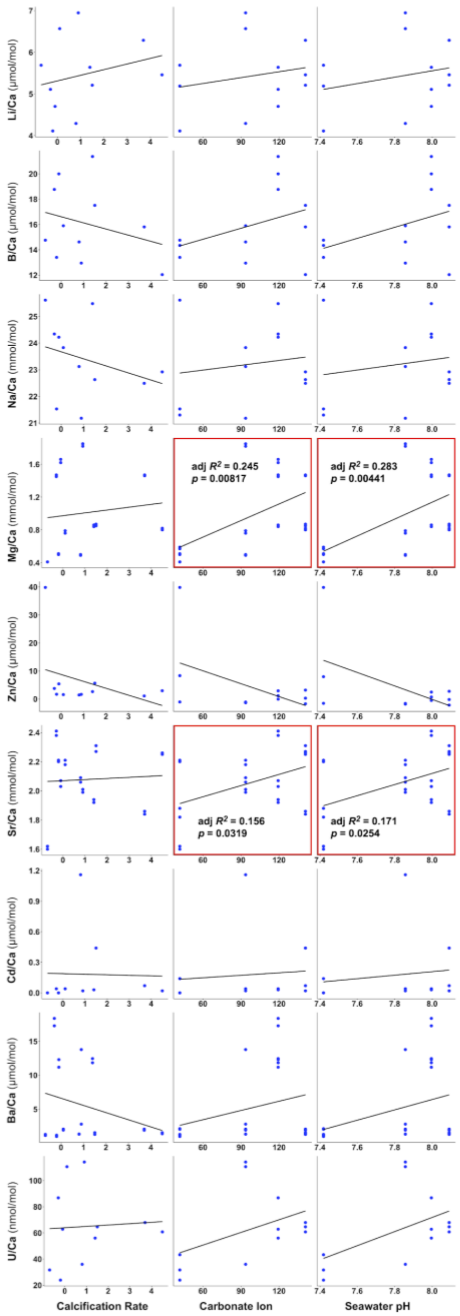

Figure S44. Array of scatterplots fitted with linear regressions displaying trace element-to-calcium ratios of whelk samples plotted against carbonate chemistry and other measured parameters. Bottom labels and labels going down the left side of the array signify x-axis parameters and y-axis parameters, respectively, for a given plot. Scatterplots with regressions that possess a p-value < 0.05 are outlined in red. Significant relationships include Mg/Ca and Sr/Ca against seawater carbonate ion concentration and seawater pH. The relationships of Sr/Ca against carbonate ion concentration and seawater pH are absent from the quadratic regression analysis.

Whelk – Quadratic Models

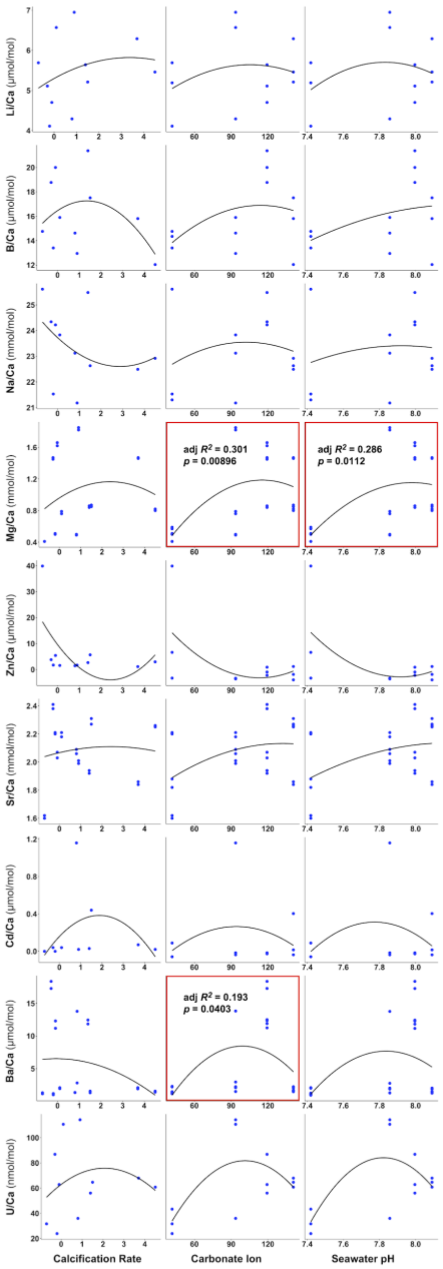

Figure S45. Array of scatterplots fitted with quadratic regressions displaying trace element-to-calcium ratios of whelk samples plotted against carbonate chemistry and other measured parameters. Bottom labels and labels going down the left side of the array signify x-axis parameters and y-axis parameters, respectively, for a given plot. Scatterplots with a regression that possess a p-value < 0.05 are outlined in red. Significant relationships include Mg/Ca and Ba/Ca against seawater carbonate ion concentration and Mg/Ca against seawater pH. Only the Ba/Ca against seawater carbonate ion chemistry is not observed in the linear regression analysis.

VI. Links to GitHub repositories containing the code used to generate the plots

A. Element-to-calcium ratio boxplots (Figure 1)
B. Scatterplots of element-to-calcium ratios vs. carbonate chemistry and other measured parameters with linear fits (Figures S9 – S44, odd numbers)
C. Scatterplots of element-to-calcium ratios vs. carbonate chemistry and other measured parameters with quadratic fits (Figures S9 – S44, even numbers)
D. Akaike Information Criterion (AIC) analysis: comparisons between linear and quadratic fits (Tables 5 – 7)
E. Non-metric multidimensional scaling (NMDS) plots (Figures 6 – 7)

VII. Phylogenetic analysis

A. Methods and results of phylogenetic trees

Methods. We inferred the phylogeny among the 18 species in the dataset using BEAST 1.10 (Suchard et al. 2018). For each species, we compiled from genetic information for nuclear (18S) and mitochondrial loci (COI and Cytb). We selected from genbank (Benson, 2003) a single sequence per gene per species (Table 1). We performed sequence alignment within loci using Muscle 3.8 (Edgar 2004) under default parameters. Next, we removed highly ambiguous regions in alignments using Gblocks v. 0.91b (Castresana, 2000) under the less restrictive parameters. Finally, the three curated alignment files were concatenated using the SuperMatrix function implemented in the evobiR R package version 1.1 (Blackmon and Adams, 2015). The resulting concatenated alignment is provided in Data S1. The gene-based partition alignment was used as input to BEAST. We used a GTR+G model of molecular evolution for each gene partition. Molecular clocks (uncorrelated log-normal) also reflected the number of gene partitions. Nevertheless, we inferred a single tree across partitions. We used an ultrametric tree for the target taxa retrieved from Time Tree of Life (Hedges et al., 2006) to seed our topology search in BEAST. Note that while our taxonomic sampling is sparse (i.e. including species from different phyla), our molecular sampling is largely biased towards fast-evolving genes (e.g. mitochondrial COI and Cytb). At this deep taxonomic scale, fast-evolving gene regions are unlikely to resolve the higher-level relationships among the species in our dataset. Furthermore, the only nuclear region (18S) is not available for all the target species (83% of 18 species). Using the Time Tree of Life database, we retrieved a phylogeny for all 18 species in our database. However, given that in nine cases, the target species were not excluded in the database, we used alternative taxa within the same target families that were sampled in the Time Tree of Life. Note that all 18 taxa belong to different families. Therefore, any species sampled in the Time Tree of Life will recover the stem group for the target family. Specifically, we sampled (1) *Metagoniolithon chara* instead of *Neogoniolithon spectabile*, (2) *Arbacia lixula* instead of *Arbacia punctulata*, (3) *Prionocidaris bispinosa* instead of *Eucidaris tribuloides*, (4) *Metapenaeus ensis* instead of *Penaeus plebejus*, (5) *Phragmatopoma californica* instead of *Hydroides crucigera*, (6) *Rapana venosa* instead of *Urosalpinx cinerea*, (7) *Oculina patagonica* instead of *Oculina arbuscula*, and (8) *Strombus gigas* instead of *Strombus alatus*. The resulting tree is provided in Data S2. Finally, we used the following priors in BEAST. We used a Yule model as the speciation prior. We constrained the age of multiple nodes across the phylogeny based on dates estimates listed in Time Tree of Life: (1) tree age (normal distribution, mean=1669 Ma, sigma=20), (2) *Argopecten irradians* + *Mercenaria mercenaria* (mean=492, sigma=40), and *Crepidula fornicata* + *Urosalpinx cinerea* (mean=296, sigma=30), *Oculina arbuscula* + *Argopecten irradians* (mean=796, sigma=20). We selected the same type of prior distribution for all nodes (a normal distribution) given all these age ranges reflect secondary calibrations (Heads 2005). We analyzed 3 independent BEAST runs, each consisting of a total 30 million generation, selecting a burnin of 10% and sampling each 3,000 generations. We examined MCMC convergence using Tracer 1.7.1 (Rambaut et al. 2018; ESS on all parameters was >200; Data S4), and summarized the post-burnin posterior distribution of BEAST trees using treeAnnotator v. 1.10 (Suchard et al. 2018). The resulting XML file used to run all BEAST analyses is provided in Section VII B of the SI.

Results. We inferred phylogenetic relationships among 18 species from seven phyla (Data S5). Phylogenetic relationships were congruent with previous studies (e.g. Scholl and Wiens, 2019). For instance, our tree recovers phylum-level monophylies for phyla with more than two sampled

species (Arthropoda, Mollusca, and Echinodermata). Land plants and algae are recovered as paraphyletic (Wodniok et al. 2011). Age estimates for different nodes were also similar to those in previous studies. For example, crown ages for Malacostraca (321–400 Ma), Mollusca (544–723 Ma), and Echinodermata (234–312 Ma) overlapped with date estimates in Bracken-Grissom et al. (2013), Zapata et al. (2014), and Nowak et al. (2013), respectively. The age of the root, representing the most recent common ancestor of red algae and land plants, was also similar to that in previous studies (1627–1705 Ma; Blair et al. 2005; Yang et al. 2016; Parfrey et al. 2011).

Table S7. Tabulated ascension numbers used to access available gene sequences and build the time-calibrated phylogenetic tree.

Common name	*Species*	Taxonomy	COI	Cytb	18S
American Lobster	*Homarus americanus*	Arthropoda	KU564525		KF578397
Blue Crab	*Callinectes sapidus*	Arthropoda	MG515527	AY465916	AY743951
Gulf Shrimp	*Penaeus plebejus*	Arthropoda	AF279848		
Blue Mussel	*Mytilus edulis*	Mollusca	HM386487	EU332487	AY527062
Oyster	*Crassostrea virginica*	Mollusca	KF644323		XM_022486485
Hard Clam	*Mercenaria mercenaria*	Mollusca	MK091906		JN996711
Soft Clam	*Mya arenaria*	Mollusca	MG423079	GQ166619	AH001707
Bay Scallop	*Argopecten irradians*	Mollusca	GU120025	GQ166596	L11265
Conch	*Strombus alatus*	Mollusca	DQ525208		
Periwinkle	*Littorina littorea*	Mollusca	MG935071	EU875963	MK919696
Whelk	*Urosalpinx cinerea*	Mollusca	KF644187		
Limpet	*Crepidula fornicata*	Mollusca	MG934931		AY377660
Temperate (Purple) Urchin	*Arbacia punctulata*	Echinodermata	MN683883		AH001568
Tropical (Pencil) Urchin	*Eucidaris tribuloides*	Echinodermata	MN683935		AH001638
Coralline Red Algae	*Neogoniolithon spectabile*	Rhodophyta			AY234238
Halimeda Algae	*Halimeda incrassata*	Chlorophyta			AF525573
Serpulid worm	*Hydroides crucigera*	Annelida		KP178715	KP178701
Temperate Coral	*Oculina arbuscula*	Cnidaria			JX983594

B. Link to Nexus file for time-calibrated phylogeny for the target species

C. Additional phylogenetic signal scenarios and results

Table S8. Estimates of phylogenetic signal for X/Ca ratios from additional scenarios. An analysis for all X/Ca ratios was conducted with all X/Ca ratios together, as based on a phylogenetic principal component analysis in phytools (Revell, 2012; row="All"). Here, the goal was to explore the potential biases in the phylogenetic signal introduced by using values estimated by imputation. Cd/Ca and Na/Ca and the arthropods possess the most imputed values as elemental ratios and a

phylum of organisms, respectively. We do not observe any biases in phylogenetic signal introduced by the use of imputed values.

**Multivariate**	**Scenario**	**Lambda**
	All except Cd/Ca and Na/Ca	0.938
	All except Arthropods	0.968
	Mollusks only; B/Ca, Mg/Ca, Sr/Ca	0.9999
	Calcite only; B/Ca, Mg/Ca, Sr/Ca	0.957
	Aragonite only; B/Ca, Mg/Ca, Sr/Ca	0.988

D. Tabulated results of relative contribution comparison

Table S9. Comparison of the relative contributions of evolutionary history and mineralogy on differences in element-to-calcium ratios among species. Here, we used two alternative mineralogy categories. Mineralogy category 1 categorizes the organisms that produce either calcite, aragonite, or a mixture of the two. Mineralogy category 2 categorizes the organisms that produce either low-Mg calcite, high-Mg calcite, aragonite, or a mixture of calcite and aragonite. Below, we present the R^2 of the full phylogenetic regression model (R^2_{full}) along with the partial contributions (partial R^2s) of phylogeny ($R^2_{phylogeny}$) and mineralogy ($R^2_{mineralogy}$).

Mineralogy Category	Elemental Ratio	R^2_{full}	$R^2_{phylogeny}$	$R^2_{mineralogy}$	# Imputed Values
1	Li/Ca	0.337	9.99E-16	0.337	0
	B/Ca	0.118	0.079	0.042	4
	Mg/Ca	0.438	0.290	0.209	0
	Zn/Ca	0.135	0.000	0.135	0
	Sr/Ca	0.601	0.565	0.084	0
	Cd/Ca	0.638	0.455	0.335	5
	Ba/Ca	0.105	2.22E-16	0.105	4
	U/Ca	0.128	0.133	0.230	3
	Na/Ca	0.119	0.117	0.002	7
2	Li/Ca	0.337	-0.924	0.656	0
	B/Ca	0.118	0.070	0.247	4

	Mg/Ca	0.438	0.550	0.436	0
	Zn/Ca	0.135	0.429	0.320	0
	Sr/Ca	0.601	0.576	0.221	0
	Cd/Ca	0.638	0.791	0.560	5
	Ba/Ca	0.105	0.462	0.333	4
	U/Ca	0.128	0.253	0.234	3
	Na/Ca	0.119	0.157	0.010	7

VIII. List of Supplementary Files

A. Supplementary File 1 - A complete data sheet with tabs for the full dataset as well as the dataset with outliers removed.

IX. References

Benson, D.A., 2003. GenBank. *Nucleic Acids Research*, 31(1), pp.23–27.

Blair, J.E., Shah, P. & Hedges, S.B., 2005. *BMC Bioinformatics*, 6(1), p.53.

Blackmon, H., and Adams, R. H. evobiR. Available from: https://cran.r-project.org/web/packages/evobiR/index.html

Bracken-Grissom, H.D. et al., 2013. A comprehensive and integrative reconstruction of evolutionary history for Anomura (Crustacea: Decapoda). *BMC Evolutionary Biology*, 13(1), p.128.

Castresana, J., 2000. Selection of Conserved Blocks from Multiple Alignments for Their Use in Phylogenetic Analysis. *Molecular Biology and Evolution*, 17(4), pp.540–552.

Crocket, J. H., & Winchester, J. W. (1966). Coprecipitation of zinc with calcium carbonate. *Geochimica et Cosmochimica Acta*, *30*(10), 1093–1109. https://doi.org/10.1016/0016-7037(66)90119-0

DeCarlo, T. M., Gaetani, G. A., Holcomb, M., & Cohen, A. L. (2015). Experimental determination of factors controlling U/Ca of aragonite precipitated from seawater: Implications for interpreting coral skeleton. *Geochimica et Cosmochimica Acta*, *162*, 151–165. https://doi.org/10.1016/j.gca.2015.04.016

Dinapoli, A. & Klussmann-Kolb, A., 2010. The long way to diversity – Phylogeny and evolution of the Heterobranchia (Mollusca: Gastropoda). *Molecular Phylogenetics and Evolution*, 55(1), pp.60–76.

Edgar, R.C., 2004. MUSCLE: multiple sequence alignment with high accuracy and high throughput. *Nucleic Acids Research*, 32(5), pp.1792–1797.

Füger, A., Konrad, F., Leis, A., Dietzel, M., & Mavromatis, V. (2019). Effect of growth rate and pH on lithium incorporation in calcite. *Geochimica et Cosmochimica Acta*, (248), 14–24. https://doi.org/10.1016/j.gca.2018.12.040

Gabitov, R. I., Sadekov, A., & Leinweber, A. (2014). Crystal growth rate effect on Mg/Ca and Sr/Ca partitioning between calcite and fluid: An in situ approach. *Chemical Geology*, *367*, 70–82. https://doi.org/10.1016/j.chemgeo.2013.12.019

Gabitov, R. I., Schmitt, A. K., Rosner, M., McKeegan, K. D., Gaetani, G. A., Cohen, A. L., … Harrison, T. M. (2011). In situ δ7Li, Li/Ca, and Mg/Ca analyses of synthetic aragonites. *Geochemistry, Geophysics, Geosystems*, *12*(3). https://doi.org/10.1029/2010GC003322

Gabitov, R., Sadekov, A., Yapaskurt, V., Borrelli, C., Bychkov, A., Sabourin, K., & Perez-Huerta, A. (2019). Elemental uptake by calcite slowly grown from seawater solution: An In-situ study via depth profiling. *Frontiers in Earth Science*, *7*. https://doi.org/10.3389/feart.2019.00051

Heads, M., 2005. Dating nodes on molecular phylogenies: a critique of molecular biogeography. *Cladistics*, 21(1), pp.62–78.

Hedges, S.B., Dudley, J. & Kumar, S., 2006. TimeTree: a public knowledge-base of divergence times among organisms. *Bioinformatics*, 22(23), pp.2971–2972.

Holcomb, M., DeCarlo, T. M., Gaetani, G. A., & McCulloch, M. (2016). Factors affecting B/Ca ratios in synthetic aragonite. *Chemical Geology*, *437*, 67–76. https://doi.org/10.1016/j.chemgeo.2016.05.007

Katsikopoulos, D., Fernandez-Gonzalez, Á., & Prieto, M. (2008). Crystallization of the (Cd,Ca)CO 3 solid solution in double diffusion systems: the partitioning behaviour of Cd 2+ in calcite at different supersaturation rates. *Mineralogical Magazine*, *72*(1), 433–436. https://doi.org/10.1180/minmag.2008.072.1.433

Liu, Y.-W. W., Sutton, J. N., Ries, J. B., & Eagle, R. A. (2020). Regulation of calcification site pH is a polyphyletic but not always governing response to ocean acidification. *Science Advances*, *6*(5), 7–8. https://doi.org/10.1126/sciadv.aax1314

Mavromatis, V., Goetschl, K. E., Grengg, C., Konrad, F., Purgstaller, B., & Dietzel, M. (2018). Barium partitioning in calcite and aragonite as a function of growth rate. *Geochimica et Cosmochimica Acta*, *237*, 65–78. https://doi.org/10.1016/j.gca.2018.06.018

Mavromatis, V., Montouillout, V., Noireaux, J., Gaillardet, J., & Schott, J. (2015). Characterization of boron incorporation and speciation in calcite and aragonite from co-precipitation experiments under controlled pH, temperature and precipitation rate. *Geochimica et Cosmochimica Acta*, *150*, 299–313. https://doi.org/10.1016/j.gca.2014.10.024

Nowak, M.D. et al., 2013. A Simple Method for Estimating Informative Node Age Priors for the Fossil Calibration of Molecular Divergence Time Analyses. *PLoS ONE*, 8(6).

Parfrey, L.W. et al., 2011. Estimating the timing of early eukaryotic diversification with multigene molecular clocks. *Proceedings of the National Academy of Sciences*, 108(33), pp.13624–13629.

Rambaut, A. et al., 2018. Posterior Summarization in Bayesian Phylogenetics Using Tracer 1.7. *Systematic Biology*, 67(5), pp.901–904.

Ries, J. B., Cohen, A. L., & McCorkle, D. C. (2009). Marine calcifiers exhibit mixed responses to CO2-induced ocean acidification. *Geology*, *37*(12), 1131–1134. https://doi.org/10.1130/G30210A.1

Suchard, M.A. et al., 2018. Bayesian phylogenetic and phylodynamic data integration using BEAST 1.10. *Virus Evolution*, 4(1).

Weremeichik, J. M., Gabitov, R. I., Thien, B. M. J., & Sadekov, A. (2017). The effect of growth rate on uranium partitioning between individual calcite crystals and fluid. *Chemical Geology*, *450*, 145–153. https://doi.org/10.1016/j.chemgeo.2016.12.026

Wiens, J.J. & Scholl, J.P., 2019. Diversification rates, clade ages, and macroevolutionary methods. *Proceedings of the National Academy of Sciences*, 116(49), pp.24400–24400.

Wodniok, S. et al., 2011. Origin of land plants: Do conjugating green algae hold the key? *BMC Evolutionary Biology*, 11(1).

Yang, E.C. et al., 2016. Divergence time estimates and the evolution of major lineages in the florideophyte red algae. *Scientific Reports*, 6(1).

Zapata, F. et al., 2014. Phylogenomic analyses of deep gastropod relationships reject Orthogastropoda. *Proceedings of the Royal Society B: Biological Sciences*, 281(1794), p.20141739.
